# Oral hydrogel systems in lower gastrointestinal disorders: From disease-based therapy to microbiota-guided design

**DOI:** 10.1016/j.mtbio.2026.103357

**Published:** 2026-06-16

**Authors:** Haoming Wu, Jingjing Tian, Wuzheng Luo, Jiayu Liu, Yingying Chen, Kaiwen Yang, Jixin Zhou, Jingwen Li, Shuhao Yang, Yao Zhang, Kainan Li, Gaohui Zhu, Shuai Tan, Xulin Hu

**Affiliations:** a School of Preclinical Medicine of Chengdu University, Chengdu University, Chengdu, 610106, China; b Clinical Medical College and Affiliated Hospital of Chengdu University, Chengdu University, Chengdu, 610081, China; c Department of Biotherapy, Cancer Center and State Key Laboratory of Biotherapy, West China Hospital, Sichuan University, Chengdu, 610041, China; d Department of Endocrinology Children's Hospital of Chongqing Medical University, National Clinical Research Center for Child Health and Disorders, Ministry of Education Key Laboratory of Child Development and Disorders, China International Science and Technology Cooperation base of Child Development and Critical Disorders, Chongqing Key Laboratory of Child Rare Diseases in Infection and Immunity, Chongqing, 400014, China; e Yu-Yue Pathology Scientific Research Center, Jinfeng Laboratory, Chongqing, 401329, China; f Department of Traditional Chinese Medicine Children's Hospital of Chongqing Medical University, National Clinical Research Center for Child Health and Disorders, Ministry of Education Key Laboratory of Child Development and Disorders, China International Science and Technology Cooperation base of Child Development and Critical Disorders, Chongqing Key Laboratory of Child Rare Diseases in Infection and Immunity, Chongqing, 400014, China; g Department of Orthopaedics, The First Affiliated Hospital of Chongqing Medical University, No.1, Youyi Road, Yuanjiagang, Yuzhong District, Chongqing, 400016, China

**Keywords:** Oral hydrogels, Delivery systems, Lower gastrointestinal disorders, Nanomedicine, Gut microbiota

## Abstract

The lower gastrointestinal tract is a complex ecological system involved in epithelial barrier maintenance, immune regulation, microbial metabolism, and host homeostasis. Disruption of this environment is closely associated with lower gastrointestinal disorders, including inflammatory bowel disease (IBD), irritable bowel syndrome (IBS), and colorectal cancer (CRC). Conventional therapies often face limitations such as poor drug stability, low solubility, insufficient intestinal retention, limited targeting efficiency, and systemic adverse effects. Oral hydrogel systems have emerged as promising platforms for lower gastrointestinal therapy because of their biocompatibility, tunable physicochemical properties, controlled-release behavior, and responsiveness to intestinal microenvironmental cues. However, most existing studies have been developed primarily from a disease-based therapeutic perspective, focusing on inflammation suppression, oxidative stress regulation, epithelial barrier repair, tumor inhibition, or symptom relief, while gut microbiota alterations are often evaluated as accompanying outcomes. In this review, we discuss oral hydrogel systems for lower gastrointestinal disorders from the perspective of the transition from disease-based therapy to microbiota-guided design. We summarize the physiological barriers and design basis of oral hydrogel systems, disease-oriented applications in IBD, CRC, gut–brain axis-related disorders, and microbiota-associated metabolic disorders, and emerging microbiota-oriented hydrogel strategies. We further discuss hydrogel–microbiota–host interactions, microbiota-related design principles, and translational challenges. This review aims to clarify how oral hydrogel systems can evolve from conventional drug delivery platforms toward microbiota-guided therapeutic systems for lower gastrointestinal disorders.

## Graphical abstract

Schematic overview of oral hydrogel systems for lower gastrointestinal disorders.

**Figure:**
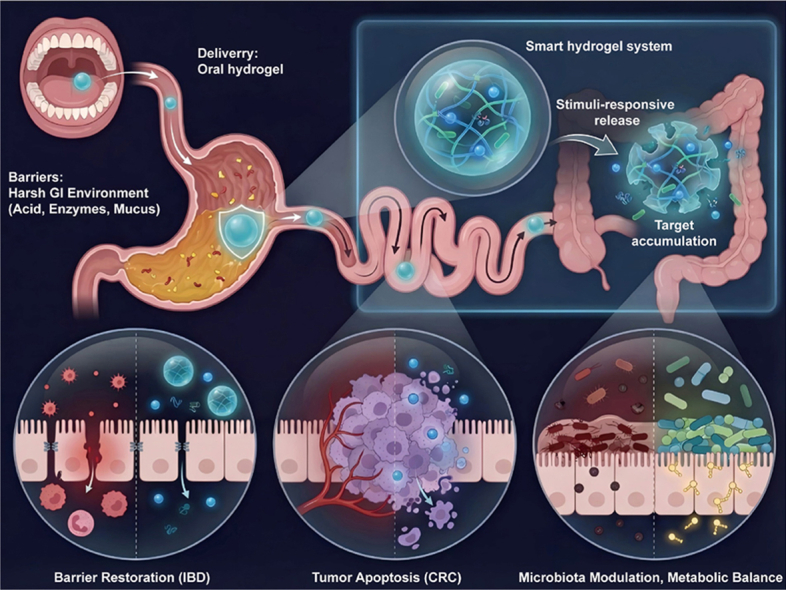

## 1 Introduction

The intestine is a vital organ for digestion, absorption, immune defense, and metabolic homeostasis. Its physiological function depends not only on epithelial integrity and immune regulation, but also on the dynamic balance of the intestinal microenvironment, which is composed of mucus layers, epithelial cells, immune components, gut microbiota, and microbial metabolites [1,2]. Among different intestinal regions, the lower gastrointestinal (GI) tract, including the distal small intestine, cecum, colon, and rectum, is particularly important. It represents a major site of microbial colonization and is frequently affected by chronic intestinal diseases [3,4]. Disruption of lower-GI homeostasis by environmental factors, dietary patterns, host genetics, infections, or immune dysregulation is closely associated with lower-GI disorders, including inflammatory bowel disease (IBD), irritable bowel syndrome (IBS), and colorectal cancer (CRC), which have become major global health concerns [[5], [6], [7], [8], [9]].

Current therapeutic strategies for lower-GI disorders continue to evolve, but effective disease management remains challenging. Oral administration is clinically attractive because it is non-invasive and generally associated with high patient compliance. However, many therapeutic agents still suffer from poor stability, low solubility, limited bioavailability, premature degradation, and insufficient accumulation at diseased intestinal sites [10,11]. For example, in IBD treatment, conventional drugs such as mesalamine can alleviate inflammation, but their degradation in the gastrointestinal tract and systemic absorption may lead to adverse effects, including gastrointestinal discomfort and hepatotoxicity [12,13]. Orally administered small-molecule drugs often show poor targeting efficiency and may require high doses to achieve therapeutic concentrations, thereby increasing the risk of off-target toxicity [14]. To address these limitations, advanced drug delivery systems have been developed to improve drug protection, site-specific release, local retention, and therapeutic precision [15,16]. Hydrogels have emerged as promising oral delivery platforms because of their excellent biocompatibility, structural tunability, high water content, and controllable physicochemical properties [17,18]. Their three-dimensional polymeric networks can encapsulate small molecules, proteins, antibodies, nanoparticles, probiotics, natural compounds, and other bioactive agents. In addition, hydrogels can be engineered to respond to intestinal microenvironmental cues, such as pH gradients, enzymes, reactive oxygen species, bacterial enzymes, and inflammatory signals [19,20]. For instance, Lai et al. [21] constructed an inflammation microenvironment-responsive edible probiotic hydrogel to achieve “antibiotic-free” clearance of *Helicobacter pylori*. These properties enable oral hydrogels to protect therapeutic cargos during upper gastrointestinal transit, prolong intestinal residence, and promote localized release in diseased regions.

Nevertheless, the therapeutic performance of oral hydrogel systems cannot be fully understood by focusing only on drug delivery efficiency or local disease suppression. The lower-GI tract is a complex ecological niche consisting of mucus layers, epithelial barriers, immune cells, dense microbial communities, and microbial metabolites. The gut microbiota plays an essential role in host defense, immune modulation, pathogen resistance, epithelial barrier maintenance, and metabolic regulation [22]. Microbial imbalance, characterized by reduced beneficial bacteria, expansion of pathogenic taxa, and altered microbial metabolic activity, is closely associated with chronic inflammatory disorders, metabolic diseases, immune dysfunction, and tumor progression [23]. In IBD, gut microbiota dysbiosis is a major contributor to disease onset and progression. Patients with IBD often exhibit reduced microbial diversity and increased abundance of specific pathogens, such as *Escherichia coli*, which correlate with disease severity and clinical symptoms [24]. In IBS, microbial disturbances may affect gut motility, visceral sensitivity, and pain perception [25]. In CRC, alterations in microbial composition and metabolites can contribute to tumor initiation, immune remodeling, and therapeutic response [26]. These findings indicate that gut microbiota modulation provides not only a new interventional dimension for lower-GI diseases, but also a theoretical basis for transforming oral hydrogels from conventional drug delivery systems into microecology-regulating platforms.

Most existing oral hydrogel systems for lower-GI disorders have been designed primarily according to disease-associated pathological features, such as inflammation, oxidative stress, epithelial barrier disruption, tumor progression, or metabolic dysfunction [27]. In many studies, gut microbiota changes are measured after treatment and interpreted as microbiota-associated therapeutic outcomes. However, these changes are not always predefined as the original design targets [28,29]. This situation raises an important question: are microbiota alterations merely consequences of disease remission, active contributors to therapeutic efficacy, or actionable targets for next-generation hydrogel design? Addressing this question requires a shift from conventional disease-based hydrogel therapy toward microbiota-guided hydrogel design.

In this review, we reorganize current oral hydrogel studies under the framework of “from disease-based therapy to microbiota-guided design.” Compared with previous reviews that mainly focused on hydrogel-based targeted delivery across different GI regions, natural polysaccharide-based colon-targeted hydrogel systems, or smart oral drug delivery systems for IBD [27,30,31], this review places greater emphasis on distinguishing disease-centered therapeutic effects, treatment-associated microbiota alterations, and microbiota-oriented hydrogel design strategies for lower-GI disorders. First, we summarize the physiological barriers, ecological features, and material design basis of oral hydrogel systems for lower-GI delivery. Second, we discuss disease-based hydrogel applications in IBD, CRC, gut–brain axis-related disorders, and gut microbiota-associated metabolic disorders, with particular attention to associated microbiota alterations and microbiota–metabolite pathways. Third, we highlight emerging microbiota-oriented hydrogel strategies, including probiotic-protective hydrogel systems and hydrogel systems that regulate the gut microbiota from a metabolite-oriented perspective. Finally, we discuss hydrogel–microbiota–host interactions, functional targets for microbiota-guided hydrogel design, and translational challenges. By distinguishing disease-centered therapeutic effects from microbiota-associated outcomes and microbiota-oriented design strategies, this review aims to provide a clearer framework for developing next-generation oral hydrogel systems for lower-GI disorders.

## 2 Design basis of oral hydrogel systems for lower-GI delivery

**Fig. 1:**
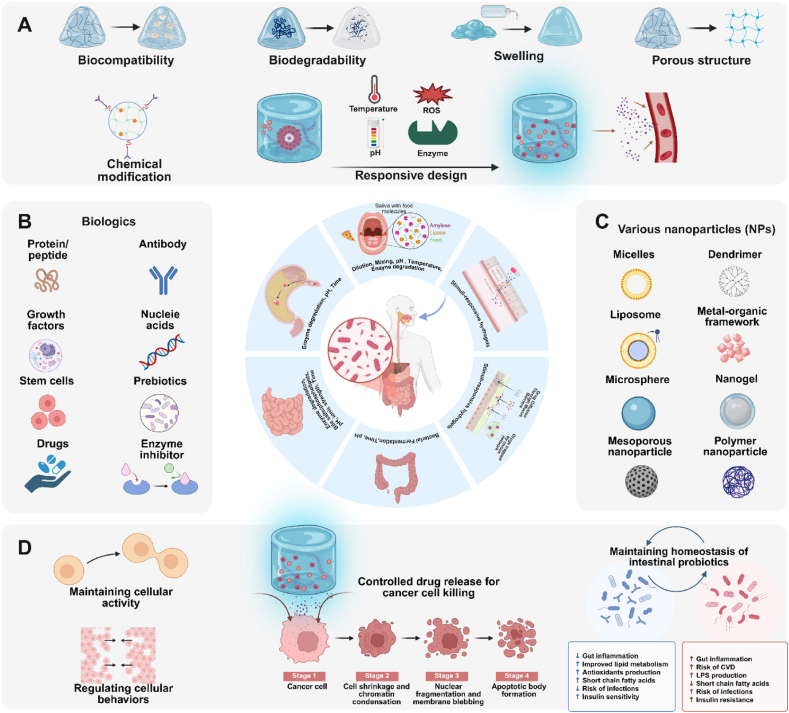
Composition and application challenges of oral hydrogel systems for lower-GI delivery. The center of the figure shows the physicochemical barriers and physiological barriers encountered by lower-GI hydrogel systems. (A). Properties of hydrogels suitable for lower-GI tract delivery, including biocompatibility and GI-responsive behavior. (B). Selection of bioactive agents for oral hydrogel systems, such as proteins, antibodies, and stem cells. (C). Selection of nanoparticles for oral hydrogel systems, including liposomes and microspheres. (D). Applications of oral hydrogel systems in lower-GI disorders, including promotion of cell proliferation, modulation of the inflammatory microenvironment, and inhibition of cancer progression. Created with BioRender.

Oral hydrogel systems for lower-GI delivery should be designed according to both the barriers encountered during gastrointestinal transit and the functional components that determine hydrogel performance. After oral administration, hydrogels pass through multiple GI segments before reaching the lower intestine, and their delivery performance is affected by region-specific physicochemical, biological, and physiological conditions, including pH variation, digestive enzymes, bile salts, mucus and epithelial barriers, microbial enzyme activity, intestinal motility, and variable residence times. These barriers determine whether hydrogels can maintain structural stability, adhere to intestinal surfaces, and achieve sustained or stimuli-responsive release at target sites. In parallel, therapeutic efficacy also depends on the rational selection of hydrogel matrices, bioactive agents, and nanoparticle-based functional components. As summarized in Fig. 1, this chapter first discusses the major barriers affecting oral hydrogel delivery in the GI tract and then introduces the key components of oral hydrogel systems, including hydrogel types and design-relevant properties, bioactive agents for disease therapy and microbiota modulation, and nanoparticle-integrated hydrogel systems.

### 2.1 Barriers to oral hydrogel systems in the lower-GI tract

After being taken orally, intestinal hydrogels traverse the GI tract and encounter sequential stages: swallowing in the oral cavity, enzymatic digestion in the stomach, further degradation and absorption in the small intestine, and eventual excretion through the colon [32]. During this journey, the functional performance of hydrogels is impeded by two primary categories of barriers: (1) physicochemical and biological barriers and (2) physiological barriers. These include enzymatic degradation, pH fluctuations, and variations in ion concentrations across different regions of the GI tract [33,34]. To achieve optimal therapeutic efficacy, hydrogels need to exhibit strong adhesion to intestinal surfaces and maintain sustained, stable functionality throughout their passage in the GI tract.

#### 2.1.1 Physicochemical barriers

Upon oral administration, intestinal hydrogels first encounter the oral cavity, which is generally neutral, with a pH around 7.4. Salivary fluids contain various ions, which may alter the charge properties of hydrogels and influence electrostatic interactions between hydrogel and encapsulated compounds, potentially affecting the hydrogel's structural integrity and functionality [35]. Saliva also contains biologically active enzymes, such as amylase and lysozyme, which may interact with the hydrogel network and influence the stability or release of embedded therapeutic agents. Notably, the physicochemical properties of hydrogels significantly affect their interaction with oral surfaces, such as the tongue and teeth. For instance, Janus hydrogels exhibit distinct surface-dependent behaviors in the oral environment [36,37]. Variations in these properties can alter the residence time of hydrogels in the oral cavity, which in turn may influence drug absorption and therapeutic efficacy. Therefore, when designing oral hydrogel-based drug delivery systems, it is essential to consider key variables in the oral cavity, including temperature, salivary composition, pH, enzymatic activity, and mechanical factors such as friction [38,39].

After passing through the oral cavity and esophagus, hydrogels reside in the stomach. Under fasting conditions, gastric acid maintains a pH of approximately 1.5, which helps inactivate harmful bacteria. During food intake, the pH rises to a range of 3.0 to 5.0 [40]. Gastric fluid also contains various ions, such as calcium, potassium, and sodium, as well as digestive enzymes including proteases and lipases, which alter the physicochemical properties of hydrogel systems and may subsequently affect the stability and activity of encapsulated bioactive compounds [41]. Additionally, the stomach generates dynamic mechanical forces via peristalsis, which may compromise the structural integrity of hydrogels. These mechanical and physiological factors, along with the nature of the ingested material, contribute to variations in gastric residence time, ranging from a few minutes to several hours [42]. During this digestive process, most solid food particles are progressively broken down by mechanical forces, chemical reactions, and enzymatic activity [43]. When designing oral hydrogel systems, it is essential to consider the harsh physicochemical and physiological environment of the stomach. First, the selection of bioactive agents plays a crucial role. Certain compounds exhibit inherent resistance to acidic pH and enzymatic degradation, allowing for effective gastric release without compromising bioactivity. In contrast, some agents are highly sensitive to gastric conditions and may be inactivated upon exposure [44]. Second, the gastric retention time of hydrogels must be taken into account. For therapies requiring sustained release, longer retention is desirable. Conversely, for acid-sensitive agents, rapid release through the stomach may enhance therapeutic effects [44].

Following gastric transit, hydrogels enter the small intestine, where the pH is nearly neutral and the environment is rich in digestive enzymes, bile salts, and bicarbonate ions. The small intestine is the primary site for nutrient digestion and absorption [45]. In this region, hydrogels are often exposed to enzymatic activity and ionic interactions, which may lead to partial drug release. Moreover, the surface-active bile salts present in intestinal fluid can destabilize hydrogel structures [46]. For instance, a sudden pH shift from the acidic stomach to the near-neutral or mildly alkaline environment of the small intestine can trigger the disintegration of peptide-loaded nanoparticles (NPs), leading to premature drug leakage from the delivery system. This risk is particularly relevant because intestinal pH gradually increases from approximately 5.5–6.5 in the duodenum to approximately 7.0–8.0 in the ileum [47]. Therefore, for small intestine-targeted hydrogel systems, it is crucial to maintain drug integrity during transit through the oral cavity and stomach, ensuring that release occurs only upon reaching the intestinal environment [48,49]. To achieve this goal, various polysaccharides, particularly dietary fibers, can be employed in hydrogel construction due to their resistance to enzymatic degradation in the upper gastrointestinal tract. These polysaccharide-based polymers can undergo selective degradation in the small intestine, driven by changes in their intermolecular interactions. In addition, pH-responsive hydrogel-based drug delivery systems can be designed by leveraging the charge properties of both the hydrogel matrix and the encapsulated bioactive agents. This approach enables the hydrogel to remain stable under acidic gastric conditions while undergoing degradation and releasing its payload in the more neutral environment of the small intestine [50,51].

In the GI tract, the colon harbors the highest concentration of microbiota, with microbial density far exceeding that of other regions of the digestive system [52]. For colon-targeted drug delivery systems, it is essential to ensure that the bioactive compounds carried by hydrogels remain stable and active throughout their transit in the upper GI tract [53]. Therefore, a key consideration is the identification of digestion-resistant hydrogel materials. Most conventional nutrients, such as starch and proteins, are readily degraded in the upper GI tract, making them generally unsuitable for constructing delivery systems. On the other hand, natural polysaccharides such as alginate (Alg) and cellulose-based hydrogels exhibit strong resistance to enzymatic degradation in the stomach and small intestine, making them well-suited for drug delivery applications targeting the colon [54,55].

In summary, the design of oral hydrogel systems for lower-GI delivery must address the challenge of overcoming physiological barriers in the gastrointestinal tract. Furthermore, the high variability in drug absorption within the GI tract must be considered. Due to the dynamic nature of intestinal motility, the onset of action of orally administered hydrogels can be influenced by factors such as food intake, drug size, composition, and timing. Therefore, it is necessary to design disease-specific targeted delivery strategies based on the physical and chemical properties of hydrogels to achieve site-specific retention of bioactive agents.

#### 2.1.2 Physiological barriers

The development of insulin delivery strategies reflects the efforts and challenges of researchers to address physiological barriers in drug delivery. The first oral administration of insulin was reported in 1923. Since then, numerous strategies have been developed, including the use of permeability enhancers. By the end of 2023, insulin delivery had evolved into several advanced forms, such as oral peptide-based insulin, oral glucose-responsive insulin, and sustained-release insulin microneedles [[56], [57], [58]]. However, low bioavailability remains a major challenge for the clinical application of oral insulin. In addition to the physicochemical barriers discussed earlier, physiological barriers, such as the mucus layer and the tightly packed intestinal epithelium, also hinder effective drug delivery [59]. Overcoming these physiological barriers is crucial for enhancing drug delivery efficiency and improving the therapeutic efficacy of hydrogel drug delivery systems.

The gastrointestinal mucus barrier is primarily secreted by goblet cells and is essentially a complex hydrogel composed of water, glycoproteins (mucins, ∼2–5% w/v), salts, lipids, and other components. It is characterized by viscoelasticity and hydrophobicity [60,61]. The thickness of the mucus barrier varies throughout the GI tract. The colon features the thickest mucus layer, consisting of two distinct layers with an average thickness of approximately 830 μm, whereas the small intestine has a single, relatively thin (100–500 μm) and loosely organized layer that facilitates efficient nutrient absorption [62,63]. Due to the presence of oligosaccharide side chains on mucins, particularly terminal carboxyl and sulfate groups, the mucus barrier carries a net negative charge [64]. The mucin concentration influences both the thickness and structural organization of the mucus layer, ultimately determining its barrier function and the permeability of drugs across it [65,66]. Successful adhesion of nanoparticles to specific mucin sites is often a prerequisite for effective cellular uptake [67]. Moreover, the mucus layer limits the absorption of macromolecular or lipophilic drugs. Negatively charged drugs frequently exhibit poor permeability through the mucus barrier due to electrostatic interactions with the negatively charged mucins [68].

Beneath the mucus barrier lies the epithelial barrier, a biological interface composed of epithelial cells that regulates the exchange of nutrients and circulating substances within the GI tract while playing a critical role in the initiation and maintenance of innate immunity [69,70]. The epithelial layer consists of various specialized cell types, including goblet cells, Paneth cells, enteroendocrine cells, tuft cells, enterocytes, and microfold (M) cells. These cells are tightly packed and interconnected by tight junction proteins, which are essential for maintaining the structural and functional integrity of the epithelial barrier [71,72]. For instance, peptide-based drugs can only be absorbed into the bloodstream after traversing the epithelial cell layer. However, the epithelial membrane acts as a selective barrier to prevent the entry of foreign particulate drugs, particularly those with high molecular weight or hydrophilic characteristics, which face significant challenges in crossing the intestinal epithelium [73].

In summary, the gastrointestinal physiological barriers, including the mucus layer and the epithelial barrier, play crucial roles in maintaining intestinal homeostasis but also pose significant challenges for oral drug delivery [74,75]. The negatively charged, viscoelastic mucus limits the penetration of large or hydrophilic drug carriers, while the tightly connected epithelial cells further restrict systemic absorption of therapeutic agents [76,77]. A comprehensive understanding of these barriers is essential for the rational design of hydrogel systems, which can enhance the therapeutic efficacy of hydrogel-mediated drug delivery.

#### 2.1.3 Regional heterogeneity of the lower-GI tract and hydrogel design implications

Although the lower-GI tract is often discussed as a single target region, the distal small intestine, cecum, and colon represent distinct physiological and ecological microenvironments. In healthy individuals, intestinal pH changes regionally, shifting from approximately 5.5–6.5 in the duodenum to approximately 7.0–8.0 in the ileum. In contrast, the cecal pH often decreases to around or below 6.0 because of microbial fermentation and short-chain fatty acid (SCFA) production [78]. The proximal colon shows a broad and highly variable pH range of approximately 5.0–7.5, whereas the distal colon generally shifts toward approximately 6.0–8.0, partly due to SCFA absorption and bicarbonate secretion [79]. Microbial density is relatively low in the proximal small intestine but increases markedly toward the distal ileum, where it may reach approximately 10^7^–10^8^ CFU/mL. By contrast, the colon contains the highest microbial biomass, commonly reaching approximately 10^11^–10^12^ CFU/mL, and is dominated by anaerobic taxa such as *Bacteroidaceae* and *Lachnospiraceae* [80]. Moreover, small intestinal transit is relatively short, generally around 3–4 h, whereas colonic transit can last much longer and varies substantially among individuals.

Taken together, these regional differences indicate that hydrogels for lower-GI delivery can be designed with pH-triggered release properties, with the option of incorporating additional microenvironment-adapted functional features, such as delayed swelling, bacterial enzyme-mediated degradation, time-dependent erosion, and mucus-adapted surface properties. For the distal small intestine, hydrogel networks should avoid premature swelling and burst release under neutral-to-alkaline conditions. For the cecum, increased microbial activity and fermentation-associated pH reduction may be exploited as pre-activation cues. For the colon, high bacterial density, prolonged residence time, and a relatively thick, two-layered mucus barrier favor microbiota-responsive degradation, sustained local release, and optimized mucoadhesion or mucus penetration. Beyond regional physiological differences, disease-specific pathological heterogeneity also shapes hydrogel design. For example, IBD requires stronger emphasis on inflamed-site retention, barrier repair, and immune modulation, whereas CRC may require tumor-localized accumulation, tissue penetration, and antitumor drug release. In IBS and microbiota-associated metabolic disorders, microbiota modulation, metabolite delivery, and long-term ecological regulation may be more relevant than aggressive lesion targeting. These disease-specific design priorities are summarized in Table 1.

**Table 1:** Disease-specific design priorities of oral hydrogel systems for lower-GI disorders.

Disease	Main pathological features	Hydrogel design priorities	Microbiota-related considerations	References
IBD	Chronic inflammation, epithelial barrier injury, mucus disruption, oxidative stress, immune-cell infiltration	Inflamed-site retention, balanced mucoadhesion/mucus penetration, ROS- or enzyme-responsive release, epithelial repair, anti-inflammatory delivery, immune modulation	Dysbiosis is both a disease-associated feature and a potential therapeutic target; microbiota changes should be linked to barrier repair, SCFA recovery, and immune rebalancing	[81,82]
CRC	Tumor growth, abnormal epithelial proliferation, tumor-associated inflammation, altered tumor microbiota, dense extracellular matrix	Tumor-localized retention, improved tissue penetration, controlled chemotherapeutic release, immunomodulation, tumor microenvironment remodeling	Tumor-associated bacteria and microbial metabolites may influence tumor progression and therapeutic response; microbiota modulation should be evaluated together with antitumor efficacy	[83,84]
IBS/gut–brain axis-related disorders	Motility disturbance, visceral hypersensitivity, neuroimmune signaling, altered microbial metabolites	Sustained local release, probiotic/prebiotic protection, metabolite delivery, mild microbiota modulation, regulation of gut–brain axis-related signaling	Focus on microbial metabolites, gut–brain signaling molecules, serotonin-related pathways, and functional outcomes rather than lesion accumulation	[85,86]
Microbiota-associated metabolic disorders	Low-grade inflammation, impaired glucose/lipid metabolism, altered bile acid and SCFA metabolism, endotoxemia	Long-term release, oral stability, probiotic/prebiotic/postbiotic delivery, metabolic modulator delivery, ecological regulation rather than lesion-specific targeting	Emphasis on functional microbiota reshaping, including SCFA production, bile acid transformation, endotoxin reduction, glucose/lipid metabolic improvement, and insulin sensitivity	[22,87]

### 2.2 Composition and functional components of oral hydrogel systems

#### 2.2.1 Types of hydrogels and design-relevant properties

Different types of hydrogels provide distinct structural and functional advantages for oral intestinal delivery. Their material features largely determine drug protection, regional responsiveness, mucosal interaction, and release kinetics. For GI applications, commonly used hydrogel materials can be broadly classified into natural hydrogels, synthetic or semi-synthetic hydrogels, and hybrid systems. Natural polymer-based hydrogels, including Alg, chitosan, hyaluronic acid (HA), gelatin, pectin, inulin, guar gum, and cellulose derivatives, are widely used because of their favorable biocompatibility, biodegradability, mucoadhesive potential, and responsiveness to intestinal enzymes or microbiota [88,89]. In particular, many natural polysaccharide hydrogels are suitable for lower-GI delivery because they can resist digestion in the stomach and small intestine while undergoing microbial enzyme-mediated degradation in the colon. Synthetic or semi-synthetic hydrogels, such as poly(acrylic acid), poly(ethylene glycol), and poly(N-isopropylacrylamide)-based networks, offer more controllable mechanical strength, mesh size, swelling behavior, and degradation profiles [90,91]. Hybrid hydrogel systems further combine the advantages of natural and synthetic materials and can incorporate nanoparticles, liposomes, or other functional components to achieve multi-responsive and site-selective intestinal delivery.

For oral intestinal delivery, the key properties of different hydrogel types should be considered in relation to the dynamic GI environment rather than as isolated material characteristics. Swelling behavior regulates water uptake, network expansion, and payload diffusion, thereby determining whether drug release occurs prematurely in the small intestine or is delayed until the cecum or colon [92]. Porous structure and mesh size influence drug-loading capacity, molecular diffusion, and the protection of bioactive agents such as proteins, antibodies, probiotics, and nanoparticles. Biocompatibility is essential for minimizing mucosal irritation and maintaining epithelial integrity, especially in inflamed or ulcerated intestinal tissues. Biodegradability further affects the residence time and clearance behavior of hydrogel systems. Degradation-triggered release can be tailored by adjusting polymer composition, crosslinking density, and enzyme-sensitive linkages [93]. In addition, stimuli-responsive properties, including pH-, enzyme-, and mucus-responsive behaviors, enable hydrogels to adapt to region-specific intestinal cues and support site-selective release [94]. Therefore, rational selection of hydrogel types should balance upper-GI stability, lower-GI activation, mucosal retention, controlled degradation, and biosafety to meet the therapeutic requirements of different lower-GI disorders. To further clarify material selection for oral lower-GI delivery, Table 2 compares natural polysaccharide/protein-based hydrogels, synthetic or semi-synthetic hydrogels, and hybrid hydrogel systems. The comparison includes hydrogel type, representative materials, main advantages, main limitations, and potential clinical suitability. This comparison may help guide the choice of hydrogel platforms according to specific therapeutic requirements, such as long-term microbiota modulation, repeated administration, short-term anti-inflammatory treatment, or localized antitumor therapy.

**Table 2:** Comparison of natural, synthetic, and hybrid hydrogels for oral lower-GI delivery.

Hydrogel type	Representative materials	Main advantages	Main limitations	Potential clinical suitability	References
Natural polysaccharide/protein-based hydrogels	Alginate, chitosan, HA, gelatin, pectin, inulin, guar gum, cellulose derivatives	Good biocompatibility, biodegradability, mucoadhesion, microbial enzyme responsiveness, potential prebiotic-like effects after degradation	Relatively weak mechanical strength, batch-to-batch variability, limited control over mesh size and degradation rate, possible premature swelling	Suitable for long-term or repeated administration, microbiota modulation, probiotic delivery, and mild-to-moderate chronic intestinal disorders	[[95], [96], [97]]
Synthetic or semi-synthetic polymer hydrogels	Poly(acrylic acid), PEG-based hydrogels, PNIPAM-based networks, Eudragit-containing systems	Tunable mesh size, mechanical strength, swelling behavior, and release kinetics; good reproducibility and design flexibility	Lower intrinsic bioactivity, possible concerns about long-term biocompatibility or non-degradable residues, limited microbiota interaction	Suitable for short-term targeted therapy, precise release of potent drugs, antitumor therapy, or emergency anti-inflammatory intervention	[98,99]
Hybrid hydrogel systems	Natural–synthetic composites, hydrogel–nanoparticle systems, liposome- or MOF-integrated hydrogels, polysaccharide–PEG networks	Combine biocompatibility of natural polymers with mechanical/release controllability of synthetic systems; allow multi-responsive and hierarchical delivery	More complex formulation, higher manufacturing difficulty, possible regulatory challenges, component compatibility issues	Suitable for complex diseases requiring combined therapy, such as IBD, CRC, microbiota-associated metabolic disorders, or multi-stage release systems	[100,101]

#### 2.2.2 Bioactive agents for disease therapy and microbiota modulation

Bioactive agents are the therapeutic core of oral hydrogel systems and determine whether the system primarily functions as an anti-inflammatory, antitumor, regenerative, metabolic, or microbiota-modulating platform. Common lower-GI disorders, including IBD, IBS, CRC, and metabolic diseases, are characterized not only by local pathological changes such as inflammation, epithelial injury, abnormal proliferation, and dysmotility, but also by varying degrees of gut microbiota dysbiosis. Therefore, the selection of bioactive agents should consider both direct disease intervention and indirect regulation of the intestinal microenvironment. According to their therapeutic functions, bioactive agents used in oral hydrogel systems for lower-GI delivery can be broadly classified into pharmacological agents, regenerative bioactive factors, living biotherapeutics, and microbiota-modulating compounds.

Pharmacological agents remain the most widely used bioactive agents for lower-GI disorders. In IBD, anti-inflammatory and immunosuppressive agents such as mesalamine, azathioprine, corticosteroids, and anti-TNF-α biologics can suppress excessive immune activation and alleviate mucosal inflammation [102]. In CRC, chemotherapeutic and targeted agents, including 5-fluorouracil, oxaliplatin, camptothecin, bevacizumab, or immune checkpoint inhibitors, are used to inhibit tumor growth and remodel the tumor microenvironment [103]. For IBS and metabolic disorders, agents such as antispasmodics, metformin, insulin-related therapeutics, or lipase inhibitors may help regulate intestinal motility, glucose metabolism, or lipid absorption [104]. Encapsulation of these agents into hydrogels can improve local retention, protect drugs from premature degradation, reduce systemic exposure, and enable site-selective release in response to pH, enzymes, inflammation, or microbial activity.

Regenerative bioactive factors, including growth factors, cytokines, therapeutic proteins, nucleic acids, and stem cell-derived components, are increasingly incorporated into hydrogel systems to promote epithelial repair and mucosal reconstruction. Growth factors such as epidermal growth factor and transforming growth factor-β can stimulate epithelial proliferation, enhance barrier restoration, and attenuate inflammatory injury. Similarly, proteins, peptides, siRNA, mRNA, extracellular vesicles, and stem cells or stem cell-derived secretomes can be delivered by hydrogels to regulate immune responses, support tissue regeneration, or modulate disease-related molecular pathways [105]. In this context, hydrogels provide a protective microenvironment that preserves bioactivity, prolongs residence time, and enables sustained release or localized activation at damaged intestinal sites.

Living biotherapeutics and microbiota-modulating agents are particularly relevant for microbiota-guided hydrogel design. Probiotics, engineered bacteria, prebiotics, and microbial metabolites can be incorporated into hydrogels to restore microbial homeostasis, enhance colonization resistance, reinforce epithelial barrier integrity, and regulate host immunity. For example, probiotic-loaded hydrogels can protect bacteria from gastric acid and bile salts, improve their survival during GI transit, and promote their release in the cecum or colon. Prebiotics such as inulin, pectin, or resistant polysaccharides can serve both as hydrogel components and as fermentable substrates for beneficial bacteria, thereby increasing SCFA production. In addition, antioxidants and anti-inflammatory natural compounds, including flavonoids, selenium-based compounds, curcumin, and vitamin C, can reduce oxidative stress and inflammatory injury while indirectly reshaping the gut microbial ecosystem [106].

Beyond serving as carriers for therapeutic agents or probiotics, the hydrogel matrix itself can also influence the gut microbial community after intestinal degradation. This effect is particularly relevant for natural polysaccharide-based hydrogels, such as alginate, chitosan, pectin, inulin, cellulose derivatives, and other dietary fiber-like polymers. Many of these polysaccharides resist digestion in the upper GI tract and can reach the cecum or colon, where microbial enzymes degrade them into oligosaccharides or fermentation products that act as prebiotic-like substrates. These degradation products may support the growth or metabolic activity of beneficial bacteria and contribute to SCFA production. In this way, natural polysaccharide hydrogels can exert a dual regulatory role: the hydrogel network protects and releases bioactive agents, while the degraded matrix itself helps modulate the intestinal microbial ecosystem. For comprehensive discussion on the prebiotic effects of polysaccharide-based hydrogels, refer to previous reviews [30].

Overall, the rational selection and integration of bioactive agents, together with consideration of the hydrogel matrix itself, should align with disease-specific pathological mechanisms and lower-GI regional characteristics, thereby combining direct therapeutic effects with modulation of the gut microbial ecosystem.

#### 2.2.3 Hybrid nanoparticle–hydrogel systems

**Fig. 2:**
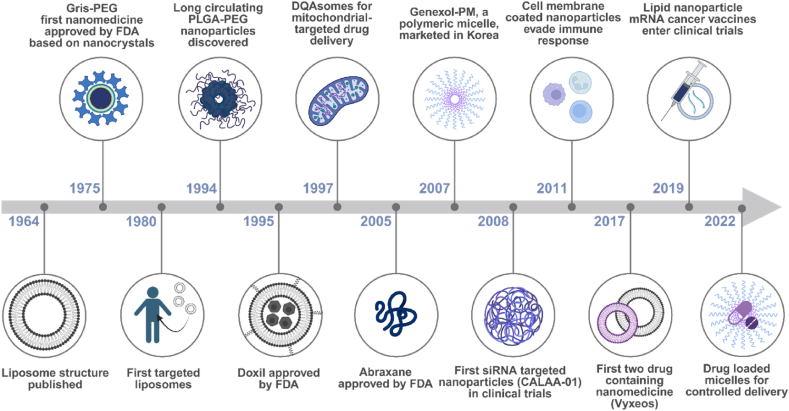
Historical timeline of key events in the field of nanomedicine. From the publication of the liposome structure in 1964 to the approval and market introduction of various nanomedicines in 2022, this timeline traces the key discoveries in nanomedicine over the past five decades. Created with BioRender.

The rapid development of nanomedicine over the past five decades has provided a broad material and technological basis for constructing nanoparticle-integrated hydrogel systems (Fig. 2). In oral intestinal delivery, nanoparticles are not only independent drug carriers but can also be incorporated into hydrogel networks to form hierarchical delivery platforms. Such systems combine the protective and adhesive properties of hydrogels with the high loading capacity, tunable surface chemistry, and targeting potential of nanoparticles [107]. This integration is particularly valuable because therapeutic agents must withstand gastric acidity, digestive enzymes, bile salts, mucus barriers, and variable transit times before reaching the lower-GI tract. Hydrogels can act as external protective networks that reduce premature nanoparticle leakage or degradation. In parallel, nanoparticles can serve as internal reservoirs to regulate payload release, improve drug solubility, enhance cellular uptake, or respond to disease-specific cues [108,109]. Therefore, nanoparticle–hydrogel systems provide a multilevel delivery strategy in which hydrogels mainly control regional retention and environmental responsiveness, whereas nanoparticles further refine drug loading, stability, and cell- or tissue-level targeting.

Various nanoparticles have been incorporated into oral hydrogel systems for lower-GI delivery, including liposomes, polymeric nanoparticles, microspheres, mesoporous silica nanoparticles, metal–organic frameworks, nanogels, and biomimetic nanoparticles. Liposomes are particularly suitable for encapsulating both hydrophilic and hydrophobic drugs and can be engineered with targeting ligands or cell-mimetic membranes to improve localization at inflamed or tumor tissues [110]. Polymeric nanoparticles and microspheres, commonly fabricated from materials such as chitosan, PLGA, or other biodegradable polymers, provide sustained release and protection for proteins, probiotics, nucleic acids, and small-molecule drugs. MOFs and mesoporous silica nanoparticles offer high surface areas, tunable pore structures, and stimuli-responsive release behavior, making them useful for pH-, ROS (reactive oxygen species)-, enzyme-, or microbiota-responsive delivery [111]. Biomimetic nanoparticles, such as cell membrane-coated nanoparticles or bacteria-derived vesicles, can further enhance immune evasion, inflammatory targeting, or microbiota interaction.

The design of nanoparticle-integrated hydrogels should match nanoparticle function with hydrogel behavior. For example, pH-responsive hydrogels can protect nanoparticles in the stomach and release them in the intestine, while enzyme- or microbiota-degradable hydrogels can enable nanoparticle exposure in the cecum or colon [112]. Mucoadhesive hydrogels can prolong local retention, whereas mucus-penetrating nanoparticles may enhance diffusion through the mucus layer and improve contact with epithelial or immune cells [113]. Such multi-level design is especially relevant for IBD, CRC, IBS, and metabolic disorders, where therapeutic efficacy depends not only on reaching the correct intestinal region but also on interacting with specific cells, inflammatory lesions, tumors, or microbial communities.

## 3 Disease-centered oral hydrogel therapies for Lower-GI disorders and associated microbiota alterations

Oral hydrogel systems have been increasingly investigated for the treatment of lower-GI disorders. Most reported systems are designed based on disease-associated pathological features. These features include intestinal inflammation, oxidative stress, epithelial barrier disruption, tumor progression, metabolic dysfunction, and gut–brain axis dysregulation. Consequently, their primary therapeutic functions include inflammation suppression, mucosal repair, localized tumor inhibition, immune remodeling, metabolic regulation, and symptom relief. At the same time, many of these studies also reported changes in gut microbiota composition or microbial metabolites after hydrogel treatment, suggesting a close relationship between disease remission and intestinal ecological restoration.

In this section, we summarize representative disease-centered oral hydrogel therapies for IBD, CRC, metabolic diseases, and gut–brain axis-related disorders, with attention to their biomaterials, loaded cargos, disease models, therapeutic mechanisms, and associated microbiota-related outcomes (Table 3). These disease-oriented studies provide the current evidence base for understanding how oral hydrogels influence the intestinal microenvironment during treatment. Building on this foundation, the following section further discusses emerging microbiota-oriented hydrogel strategies in which microbiota modulation, microbial metabolism, or ecological niche regulation becomes a more explicit design consideration.

**Table 3:** Application of oral hydrogel systems in lower-GI disorders.

	Biomaterial	Active agents	Disease model	Gut microbiota	References
IBD	Gelatin	L-arginine	DSS-induced colitis in mice	*Muribaculaceae* ↑ *Prevotellaceae-UGG-001* ↑ *Enterobacteriaceae* ↓ *Proteobacteria* ↓	[114]
	Inulin	Olsalazine	DSS-induced UC in mice; DSS-induced acute colitis in rats; Rectal injection of 2,4,6-Trinitroben -zenesulfonic acid solution inducing Crohn's disease in mice	*Bacteroidetes* ↑ *Romboutsia* ↓ *Enterococcus* ↓ *Proteobacteria* ↓ *Lachnospiraceae* ↓	[115]
	Calcium alginate (SA)	HA-modified Selenium (HA-Se)	DSS-induced colitis in mice	*Verrucomicrobiota* ↑ *Firmicutes* ↑ *Bacteroides* ↑ *Proteobacteria* ↓.	[116]
	Dopamine/thiol dual-modified hyaluronic acid (HA-SH-DA)	Basic fibroblast growth factor alanyl-glutamine	DSS-induced ulcerative colitis (UC) in mice	*Lactobacillus* ↑ *Bacteroidetes* ↓ *Helicobacter* ↓ *Proteobacteria* ↓	[117]
	SA	HA-SH	DSS-induced acute colitis in C57 BL/6 mice	*Firmicutes* ↓ *Actinomycetes* ↓ *Enterobacteriaceae* ↓ *Proteobacteria* ↑ *Lactobacillaceae* ↑ *Bifidobacteri-aceae* ↑	[118]
	Alginate-aminoethylmethylacrylate (Alg-AEMA)	Inflix-imab	DSS-induced inflammatory bowel disease in mice	*Clostridiales* ↑ *Lachnospiraceae_UCG-001* ↑ *Lachnospir- aceae_NK4A136* ↑ *Bacteroides* ↑ *Parasutterella* ↑ *Rumi-UCG-007* ↑	[119]
	Zein/SA	Bioactive glass (BG)	DSS-induced colitis in mice	*Akkermansia* ↑ *Peptostreptococcaceae*↓ *Gammaproleobacteria*↓	[120]
	Silk protein (SF) (Carrier: ethanol)	SF	DSS-induced colitis in C57BL/six mice	*Akkermansiaceae* ↑ *Dubosiella* ↑ *Ruminococcaceae* ↑ *Clostridia_UCG-014* ↑ *Oscillospiraceae* ↑ *Ileibacterium* ↑ *Proteobacteria* ↓	[121]
	SA	Manganese dioxide (MnO_2_) nanozymes Magnolol (Mag) Berberine (Ber)	DSS-induced UC in mice	*Rikenella* ↑ *Firmicutes* ↑ *Dubosiella* ↑ *Feacalibaculum* ↑ *Allobaculum* ↓	[122]
	Calcium tungstate microgel (CTM)	*Bacillus coagulans* (BC)	DSS-induced colitis in mice	*Enterobacteriaceae* ↓ *Lachnospiraceae_NK4A136_group* ↑ *Muribaculaceae* ↑	[123]
	HASH	HASH	DSS-induced acute IBD in C57BL/6 mice; DSS-induced chronic IBD in C57BL/6 mice	*Enterobacteriaceae* ↓ *Erysipelatoclostridiaceae* ↓ *Proteobacteria* ↓ *Enterobacteriaceae* ↓ *Erysipelatoclostridiaceae* ↓ *Peptostreptococcaceae*↓ *Lachnospiraceae* ↑ *Muribaculaceae* ↑ *Oscillospiraceae* ↑	[124]
	Sodium alginate microspheres (SAM) (Carrier: phosphate buffered saline, PBS)	Janus nanomotor M2 macrophage membrane(M2M)	DSS-induced colitis in mice	*Bacteroidota* ↑ *Norank_f__Muribaculaceae* ↑ *Lactobacillus* ↑ *Odoribacter* ↑ *Proteobacteria* ↓ *Escherichia-Shigella* ↓	[125]
	SA and carboxymethyl chitosan (CMCS)	*L. rhamnosus 76* (LR76)	Bacterial GI retention assays in healthy C57BL 6/J mice	*Firmicutes* ↑ *Lactobacillus* ↑	[126]
	Guar gum (GG) and low-methoxyl pectin (LMP) SA and chitosan (CS)	Curcumin (Cur)	DSS-induced UC in mice	*Proteobacteria* ↓ *Verrucomicrobia* ↓ *Bacteroidetes* ↑	[127]
	Cysteamine-grafted hyaluronic acid (HS)	Superoxide dismutase (SOD)	DSS-induced colitis in C57BL/6J mice; Rectal injection of TNBS inducing colitis in rats for 5 days	*Firmicutes* ↑ *Turicibacte* ↑ *Barnesiella* ↑ *Clostridium XIVa* ↑ *Lachnospiraceae* ↑ *Bacteroidetes* ↓ *Proteobacteria* ↓	[128]
Colon cancer	Inulin	Oxa	Orthotopic colorectal tumor-bearing mice; Subcutaneous colorectal tumor-bearing mice	*Bacteroidota* ↓ *Alistipes* ↓ *Escherichia* ↓ *Shigella* ↓ *Helicobacter* ↓ *Lactobacillus* ↑ *Lachnospiraceae* ↑ *Akkermansia* ↑	[129]
	Poly(vinyl alcohol)/CS (PVA/CS)	CD98 siRNA (siCD98), Camptothecin(CPT)	Orthotopic colon cancer-bearing mice	*/*	[130]
	Inulin	Inulin, α-PD-1 IgG	Subcutaneous colorectal tumor-bearing mice; Subcutaneous colon carcinoma-bearing mice; Subcutaneous melanoma-bearing mice; Spontaneous colorectal cancer mice	*Akkermansia* ↑ *Lactobacillus* ↑ *Roseburia* ↑ *Oscillibacter* ↓	[131]
	CS-SA	*Escherichia coli* Nissle1917(EcN)	Orthotopic colorectal tumor-bearing mice	*Lactobacillus* ↑ *Akkermansia* ↑ *Bifidobacterium* ↑ *Bacteroides* ↑ *Lactobacillus reuteri* ↑ *Helicobacter* ↓	[132]
	Sulfhydryl silane-modified bacterial cellulose nanofibers (SulBC)	cis-dichlorodiamineplatinum (CDDP)	Subcutaneous HCT116 tumor xenograft-bearing nude mice	*Prevotellaceae* ↑ *Bacteroides* ↑ *Parabacteroides* ↑ *Lachnoclostridium* ↓ *Blautia* ↓	[133]
IBS and Gut–Brain Axis-Related Disorders	Calcium alginate microspheres (Carrier: CaCl2 solution)	Quercetin ( Que)	Mouse model of high-altitude sleep disturbance	*Lactobacillus* ↑ *Lachnospira* ↑ *Bacteroidetes* ↓ *Alistipes* ↓ *Prevonella* ↓	[134]
	Hyaluronic acid and histidine (HA-His)	Luteolin (LUT)	DSS-induced colitis in mice	*Firmicutes* ↑ *Cyanobacteria* ↑ *Campilobacterota* ↑ *Faecalibaculum* ↑ *Bifidobacterium* ↑ *Helicobacter* ↓ *Desulfobacterota* ↓	[135]
	Flavonoid-amyloid fibril	EGCG	High-fat diet (HFD)-induced obesity in mice	*Desulfovibrionaceae* ↓ *Bilophila* ↓ *Oscillibacter* ↓ *Ruminiclostridium_9* ↓ *Parabacteroides* ↑ *Alistipes* ↑ *Bacteroides* ↑ *Muribaculaceae* ↑ *Akkermansia* ↑	[136]
	HA with selenocystamine (HA-Se)	Limosilactobacillus reuteri	DSS-induced colitis in mice	*Firmicutes* ↑ *Bacteroidetes* ↑ *Proteobacteria* ↓	[137]
	HA	lactic acid bacteria (LAB)	ST-induced enteritis in mice	*Salmonella* ↓ *Lactobacillus rhamnosus* ↑	[138]
	Oral superabsorbent hydrogel (OSH)	Carboxymethyl cellulose (CMC), citric acid (CA)	HFD-induced obesity and metabolic syndrome in mice	*Akkermansia muciniphila* ↑ *Turicimonas muris* ↑ *Muribaculum intestinale* ↑ *Actinobacteria* ↓	[139]
	Nitroreductase (NTR) labile peptidic	*Escherichia coli* Nissle 1917 (EcN)	DSS-induced colitis in mice	*Muribaculae* ↑ *Alistipes* ↑ *Prevotellaceae_UCG001* ↑ *Lachnospir-aceae_NK4A136* *_group* ↑ *Bacteroides* ↓ *Alloprevotella* ↓	[140]
	SA	Lutein	1.5% DSS-induced colitis in mice	*Desulfovibrionaceae* ↓ *Proteobacteria* ↓ *Lachnospiraceae* ↓ *Ruminococcaceae* ↓ *Erysipelotrichaceae* ↑ *Rikenellaceae* ↑	[141]

### 3.1 IBD: inflammation suppression, barrier repair, and microbiota-associated recovery

The two most common subtypes of IBD are Crohn's disease (CD) and ulcerative colitis (UC), both of which are characterized by chronic inflammation within the intestinal lumen accompanied by mucosal ulceration [142,143]. The etiology and pathogenesis of IBD are complex and typically involve dysbiosis of the gut microbiota, immune dysfunction, and pathogenic infections. In the pathological microenvironment of IBD, the dynamic balance between the immune system and intestinal microorganisms is often disrupted, leading to lesion formation [144,145]. Currently, the primary therapeutic goal for IBD is to achieve long-term asymptomatic remission. Traditional treatment approaches focus on immune suppression or the attenuation of acute inflammatory episodes. However, these therapies may cause adverse effects, including increased infection risk and metabolic disturbances [146,147]. Existing biologics, such as integrin inhibitors, anti-inflammatory peptides, antioxidant enzymes, and probiotics, have also shown certain levels of efficacy [148]. However, the acidic and enzyme-rich gastrointestinal environment frequently limits the bioavailability of these biologics. To address these challenges, current research is increasingly focused on the development of hydrogel systems for biologics, aiming to suppress intestinal inflammation and restore gut homeostasis.

#### 3.1.1 Anti-inflammatory and antioxidant hydrogel systems with associated microbiota recovery

Inflammation and oxidative stress are central pathological features of IBD and are closely associated with epithelial barrier disruption, immune dysregulation, and gut microbiota dysbiosis [[149], [150], [151]]. Excessive production of pro-inflammatory cytokines and ROS aggravates mucosal injury, amplifies immune activation, and delays tissue repair. Therefore, hydrogel systems capable of improving the delivery, stability, and local retention of anti-inflammatory or antioxidant agents have become important platforms for IBD treatment. In addition to enhancing local drug availability, these systems may also contribute to intestinal ecosystem restoration by linking oxidative stress relief, immune regulation, epithelial barrier repair, mucus homeostasis, and microbiota-associated metabolic recovery.

**Fig. 3:**
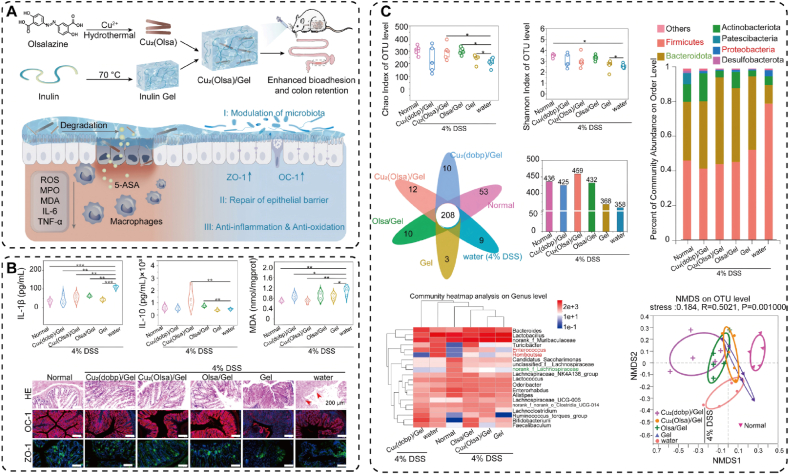
Cu_2_(Olsa)/Gel hydrogel engineered for colitis therapy. (A) Schematic illustration of the synthesis of Cu_2_(Olsa)/Gel and its proposed mechanism, showing enhanced colon retention, microbiota modulation, epithelial barrier repair, and anti-inflammatory/antioxidant effects. (B) In DSS-induced colitis mice, Cu_2_(Olsa)/Gel reduced inflammatory cytokines (IL-1β, IL-10, MDA) and improved epithelial integrity and tight junction protein expression, as shown by HE, ZO-1, and Occludin-1 staining. (C) Cu_2_(Olsa)/Gel treatment modulated gut microbiota, increasing microbial richness and diversity, restoring community structure, and reducing harmful taxa compared to control groups. Reproduced with permission [115]. Copyright 2024, Elsevier.

For small-molecule anti-inflammatory drug delivery, hydrogel-assisted nanoparticle systems can improve drug loading, reduce premature release, and enhance lesion-site accumulation. Conventional pharmacological treatments for IBD, such as corticosteroids, aminosalicylates, antibiotics, and immunosuppressants, often suffer from poor local retention and systemic side effects [152,153]. To address these limitations, Zhang et al. [115] employed olsalazine-based MOF nanoneedles embedded in an inulin hydrogel for targeted intestinal administration. The high specific surface area of the MOF nanoneedles improved drug-loading capacity and prolonged drug release, while their needle-like morphology facilitated cellular penetration and intracellular delivery. Meanwhile, the inulin hydrogel matrix enhanced colonic retention and provided a prebiotic substrate that could be degraded by colonic microbiota. In simulated GI release studies, free olsalazine sodium showed rapid release, whereas Cu_2_(Olsa)/Gel markedly reduced premature release under small-intestinal pH conditions (olsalazine sodium: 71.7% release at 4 h; Cu_2_(Olsa)/Gel: 20.3%). At 24 h after oral administration, the colon fluorescence intensity of the gel formulation was approximately 2.0-fold higher than that of inulin solution, indicating improved colonic retention. In a DSS-induced IBD mouse model, this strategy significantly reduced inflammatory responses, as shown by downregulation of NF-κB and pro-inflammatory cytokines, including TNF-α, IL-6, and IL-1β, together with increased expression of the anti-inflammatory cytokine IL-10. It also enhanced ZO-1 and Occludin-1 expression, thereby contributing to intestinal barrier preservation. Histological analysis further demonstrated reduced colonic tissue injury and decreased inflammatory cell infiltration. Moreover, microbiota analysis showed that Cu_2_(Olsa)/Gel increased microbial richness and diversity, reduced potentially harmful taxa such as *Proteobacteria*, *Romboutsia*, and *Enterococcus*, and increased *Lachnospiraceae*, which are associated with butyrate and other SCFA production. These results suggest that Cu_2_(Olsa)/Gel alleviated colitis through combined anti-inflammatory/antioxidant therapy, epithelial barrier repair, and microbiota-associated homeostasis restoration (Fig. 3).

Natural phenolic compounds have also been incorporated into hydrogel systems to combine structural stabilization with intrinsic anti-inflammatory and antioxidant bioactivity. Phenolic compounds, such as epigallocatechin gallate (EGCG), gallic acid, tyramine, and tannic acid (TA), contain abundant hydroxyl groups and can form physical interactions with polymers, through hydrogen bonding, hydrophobic interactions, or electrostatic attraction [154,155]. Based on this principle, Pan et al. [156] developed a composite hydrogel (MAG) by crosslinking EGCG with decellularized small intestinal submucosa (SIS) and HA. This design improved hydrogel mechanical strength while endowing the system with antimicrobial and anti-inflammatory properties. Compared with MA hydrogel, MAG showed slower erosion in artificial colon fluid (MA: approximately 50% degradation at 24 h; MAG: 14% erosion), stronger adhesion to colonic tissue, and prolonged retention on inflamed colonic mucosa. *In vivo* imaging showed that about 50% of MAG remained in the colitis colon at 12 h, whereas most MA was cleared by 4 h. MAG also exhibited stronger antibacterial shielding against *E. coli* than MA, as reflected by lower bacterial growth and a larger inhibition zone (OD_600_: MAG 0.31 vs. MA 0.54; inhibition zone: MAG 8.27 ± 0.15 mm vs. MA 0.90 ± 0.21 mm). In a DSS-induced colitis model, MAG suppressed pro-inflammatory cytokines, including IL-6, TNF-α, and IL-1β, restored epithelial barrier integrity by upregulating ZO-1, Occludin-1, and Claudin-5, and promoted mucosal regeneration through increased expression of Lgr5, SOX-9, and Ki67. Importantly, MAG also restored mucus secretion and functional goblet cells by increasing MUC2 expression, thereby reinforcing mucus homeostasis and reducing direct contact between gut microbiota and the damaged epithelium. Microbiota analysis further showed that MAG increased bacterial richness and diversity, shifted the microbial profile closer to that of healthy mice, and increased *Akkermansia*, a mucus-associated bacterium linked to barrier function. Therefore, MAG functioned not only as an anti-inflammatory hydrogel but also as a mucus-mimetic physical shield that coordinated antibacterial protection, mucus repair, epithelial regeneration, and microbiota-associated barrier recovery.

Protein-based antioxidant therapy is another important direction for IBD treatment, but oral delivery of proteins is limited by poor stability in the GI tract [157]. Selenoprotein is a key protein-based therapeutic involved in immune modulation and oxidative stress regulation [158], and it has attracted attention because of its ability to reduce intestinal inflammation and maintain gut barrier integrity [159]. To overcome the instability of orally delivered selenoprotein, OuYang et al. [116] developed oral calcium alginate hydrogel microbeads for the in situ synthesis of bioactive selenoprotein in the intestine. In this system, HA-modified selenium nanoparticles were encapsulated within calcium alginate hydrogel microbeads, protected from degradation in the upper GI tract, and released in the intestine. The alginate shell effectively prevented premature release in artificial gastric fluid, whereas HA-Se was rapidly released after transfer to artificial small intestinal fluid. Subsequently, selenium was converted into bioactive selenoprotein-related forms *in vivo*, with SeCys_2_ identified as the main selenium metabolite after SHSe administration. In a DSS-induced IBD mouse model, this hydrogel system downregulated pro-inflammatory cytokine secretion, reduced neutrophil and monocyte levels, enhanced Treg cell activity, and promoted repair of damaged colonic tissue. Specifically, SHSe reduced MPO-positive neutrophil infiltration, decreased epithelial apoptosis, restored ZO-1 expression, reduced TNF-α, IL-6, and IL-1β, and increased TGF-β1. Flow cytometric analysis further showed decreased pro-inflammatory monocytes and neutrophils in the colon and spleen, together with increased CD3^+^CD4^+^Foxp3^+^ regulatory T cells, indicating improved immune homeostasis. Gut microbiota analysis showed that SHSe increased bacterial richness and diversity, increased *Verrucomicrobiota*, *Firmicutes*, and *Bacteroides*, and reduced *Proteobacteria*. These findings indicate that SHSe microbeads connected hydrogel-mediated intestinal release with in situ selenoprotein synthesis, NF-κB-related inflammatory suppression, immune-cell redistribution, barrier repair, and microbiota remodeling.

Together, these anti-inflammatory and antioxidant hydrogel systems improve the local availability of small-molecule drugs, natural phenolic compounds, and protein-based therapeutics. Their design logic mainly involves sustained intestinal retention, ROS scavenging, inflammatory cytokine suppression, mucus and epithelial barrier repair, and immune regulation. When accompanied by increased SCFA-associated bacteria, *Akkermansia*, or reduced *Proteobacteria*, these systems further suggest that hydrogel-mediated anti-inflammatory therapy may contribute to broader intestinal ecosystem recovery rather than acting only as passive drug delivery.

#### 3.1.2 Barrier-repairing and mucosa-protective hydrogel systems with associated microbiota recovery

Restoration of the intestinal mucosal barrier is another central therapeutic goal in IBD treatment. The intestinal barrier is composed of epithelial cells, tight junction proteins, mucus, antimicrobial peptides, and immune components. In IBD, mucosal ulceration, mucus depletion, and tight junction disruption facilitate the translocation of luminal antigens, pathogens, and microbial products, thereby amplifying local inflammation and aggravating epithelial injury. Hydrogel systems with bioadhesive, mucosa-protective, and barrier-repairing properties have therefore been developed to promote epithelial regeneration, reinforce tight junctions, restore mucus-like protection, and reduce direct stimulation from harmful luminal factors.

Bioadhesive hydrogels are particularly suitable for mucosal repair because they can adhere to damaged intestinal tissues and prolong the residence time of therapeutic agents. These hydrogels typically bind bioactive molecules, such as growth factors, anti-inflammatory drugs, peptides, or antibodies, through chemical bonding or physical adsorption [160]. For example, negatively charged carboxyl groups (–COOH) within the hydrogel matrix can form stable complexes with positively charged anti-inflammatory peptides via electrostatic interactions, while amino groups (–NH_2_) on the hydrogel surface can form hydrogen bonds with amide groups in biologics, thereby enhancing binding affinity [161,162]. Through these interactions, bioadhesive hydrogels protect therapeutic agents from rapid degradation and support sustained release at injured mucosal sites.

Natural extracellular matrix-derived components, especially glycosaminoglycans (GAGs), such as HA, heparin, and chondroitin sulfate, have been widely used to construct mucosa-repairing hydrogels because of their favorable biocompatibility and tissue-regenerative potential [163]. For instance, HA grafted with β-cyclodextrin enabled the sustained release of insulin-like growth factor-1C (IGF-1C), thereby promoting intestinal barrier restoration after rectal administration [164].

In UC, ulcerative lesions are characterized by impaired mucosal barrier integrity, exposed epithelial surfaces, and abnormal contact between luminal microorganisms and host tissues. These pathological changes make it difficult to maintain local epithelial protection and microbial homeostasis. To reconstruct a protective interface at ulcerated sites, Hong Wen et al. [117] developed a multifunctional biomimetic hydrogel based on dopamine/thiol-modified HA, Ag^+^, fibrinogen, thrombin, basic fibroblast growth factor (bFGF), and alanyl-glutamine (Gln). The hydrogel achieved enhanced bioadhesion through thiol–silver ion coordination between antimicrobial Ag^+^ and dopamine/thiol-modified HA, and further mimicked the natural coagulation process by inducing fibrinogen activation and polymerization. During treatment, the hydrogel enabled the controlled release of bFGF and Gln, with higher cumulative release under acidic UC-like conditions than at physiological pH (Ag^+^: 85% at pH 5.5 vs. 68% at pH 7.4; bFGF: 80.62% vs. 62.79%; Gln: 75.62% vs. 60.79%). The hydrogel also showed stable intestinal adhesion, with approximately 45.15% residual hydrogel remaining after 5 days *in vivo*. At the therapeutic level, this system reduced UC severity, as reflected by decreased DAI score (DSS: 9.78; hydrogel: 4.01), restored colon length to 7.32 cm, increased body weight to 22.45 g, and reduced colonic MPO activity by 78.8% compared with the DSS group. Importantly, epithelial repair was enhanced by upregulating tight junction proteins, including occludin-1 and ZO-1, whose expression levels were 2.79-fold and 1.61-fold higher than those in the DSS group. Microbiota analysis further showed increased beneficial *Lactobacillus* and decreased harmful *Proteobacteria*, suggesting that artificial mucosal reconstruction and antibacterial regulation helped restore local microbial balance. These results indicate that the hydrogel alleviated UC by coordinating bioadhesive mucosal protection, sustained release of reparative factors, epithelial barrier repair, and microbiota recovery.

**Fig. 4:**
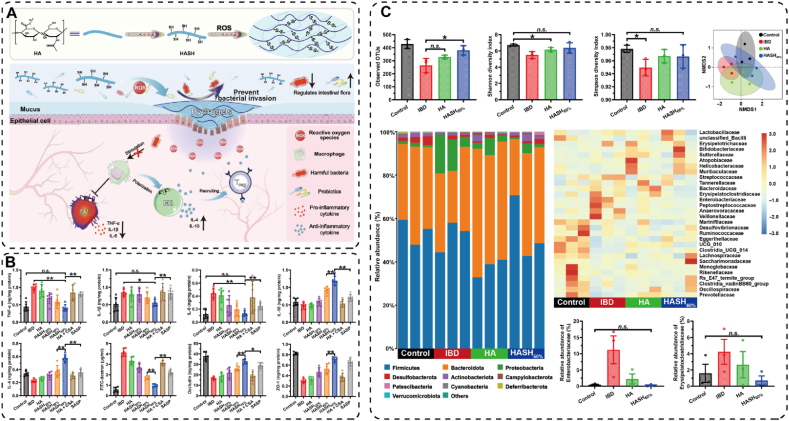
HASH hydrogel restores intestinal barrier and regulates immune responses in DSS-induced IBD. (A) Schematic illustration of ROS-responsive gelation of thiol-modified HA (HASH) forming an artificial mucus barrier, preventing microbial invasion, modulating macrophage polarization (M1 → M2), and recruiting Treg cells. (B) Colon cytokine levels (TNF-α, IL-1β, IL-6, IL-10, IL-4), FITC-dextran permeability, and tight junction proteins (occludin, ZO-1) showing improved barrier function after HASH60% treatment. (C) Gut microbiota analysis, including diversity indices, NMDS plots, relative abundance at phylum and family levels, demonstrating HASH restored microbial richness and reduced harmful taxa. Reproduced with permission [124]. Copyright 2024, American Association for the Advancement of Science.

Another important barrier defect in IBD is mucus layer disruption. Under inflammatory conditions, the mucus layer becomes thinned or lost, allowing intestinal bacteria and microbial products to directly contact epithelial cells and aggravate mucosal injury. Although hydrogel-based artificial mucus barriers have been explored, many reported systems require rectal administration, which may cause discomfort and limit patient compliance. To develop a more convenient oral artificial mucus strategy, Zhang et al. [124] designed a ROS-responsive hydrogel precursor based on thiol-modified HA (HASH). This precursor material responded to excessive ROS in inflamed intestinal regions and formed an artificial mucus layer in situ. HASH showed ROS-dependent gelation, with rapid gel formation within 1 h under high H_2_O_2_ levels, while no gel was formed within 72 h under low H_2_O_2_ conditions. HASH also remained relatively stable during GI transit, showing only about 10% viscosity decrease in simulated gastric fluid after 2 h and maintaining stability in simulated intestinal and colonic fluids for 6 h. The formed hydrogel barrier physically separated damaged mucosa from harmful microorganisms and reduced excessive microbial stimulation. *In vitro*, HASH60% almost completely blocked *E. coli* penetration. In DSS-induced IBD mouse models, HASH alleviated body weight loss, colon shortening, and reduced the MPO activity, and restored intestinal barrier function by normalizing occludin and ZO-1 expression. Microbiota analysis showed that HASH increased bacterial richness and diversity, reduced *Proteobacteria* and *Enterobacteriaceae*, and increased *Lachnospiraceae*, *Muribaculaceae*, and *Oscillospiraceae*, which are associated with SCFA production. These findings suggest that HASH functioned as an oral artificial mucus layer linking ROS-responsive gelation, mucus-like physical shielding, tight junction restoration, and SCFA-associated microbial recovery (Fig. 4).

Tight junction disruption is another important feature of IBD-associated barrier dysfunction. In UC, chronic mucosal inflammation and excessive ROS accumulation can activate myosin light chain kinase (MLCK), which promotes tight junction disassembly, increases epithelial permeability, and contributes to recurrent mucosal barrier loss. Although MLCK is a potential therapeutic target, direct enzymatic inhibition may cause toxicity, highlighting the need for localized and inflammation-responsive barrier-repairing systems. To address this issue, Wang et al. [165] developed a ROS-responsive hydrogel, ATG-CS-Gel, derived from a diselenide-bridged arctigenin–chitosan conjugate. This system was designed to target inflamed mucosa, scavenge excessive ROS, and restore epithelial barrier integrity by modulating the MLCK–tight junction pathway. ATG-CS-Gel exhibited ROS-responsive drug release, with much higher ATG release in H_2_O_2_-containing medium than in PBS alone (89.4% vs. 19.8%). After oral administration, ATG-CS-Gel preferentially adhered to inflamed colonic mucosa, with 13.0-fold higher colonic fluorescence in colitis mice than in healthy mice at 24 h. Its mucin-mediated adhesion was further supported by *in vitro* adhesion assays, in which mucin-coated surfaces retained 60.8-fold higher fluorescence than uncoated surfaces. In a DSS-induced UC mouse model, ATG-CS-Gel reduced colonic injury, extended colon length, and promoted mucosal healing, as shown by endoscopic observation of smoother mucosal surfaces, lower FITC-dextran permeability, and continuous distribution of ZO-1 and occludin-1 in the colonic epithelium. Mechanistically, ATG-CS-Gel inhibited MLCK activation and restored tight junction protein expression, indicating that it reversed mucosal barrier loss through the MLCK–tight junction pathway. Gut microbiota analysis showed that ATG-CS-Gel shifted the microbial profile closer to that of healthy mice, increased beneficial bacteria such as *Lactobacillus*, *Lachnospiraceae*, and Clostridia, and reduced pathogenic taxa such as *Bacteroides* and *Romboutsia*. These results suggest that ATG-CS-Gel suppressed colitis by integrating ROS-responsive release, inflamed-mucosa adhesion, MLCK modulation, tight junction preservation, and microbiota-associated barrier restoration.

Together, these barrier-repairing and mucosa-protective hydrogel systems mainly act by adhering to injured mucosa, forming artificial mucus-like layers, delivering reparative factors, scavenging excessive ROS, and restoring tight junction integrity. Their design logic emphasizes reconstruction of the epithelial–luminal interface, reduction of direct bacterial stimulation, and recovery of mucosal barrier function. When accompanied by increased *Lactobacillus*, *Lachnospiraceae*, *Muribaculaceae*, *Oscillospiraceae*, or Clostridia and reduced *Proteobacteria*, *Enterobacteriaceae*, *Bacteroides*, or *Romboutsia*, these systems further suggest that barrier reconstruction can support microbiota-associated restoration in IBD treatment.

#### 3.1.3 Treatment-associated microbiota and metabolite remodeling

In addition to anti-inflammatory and barrier-repairing functions, several IBD hydrogel studies have placed greater emphasis on treatment-associated microbiota remodeling and microbial metabolic recovery. These studies commonly evaluate bacterial richness and diversity, beneficial taxa, potentially harmful bacteria, and functional metabolites such as SCFAs, thereby providing additional evidence that hydrogel-mediated disease remission is accompanied by restoration of the intestinal ecological environment.

Because excessive TNF-α signaling contributes to mucosal inflammation and microbial dysbiosis in IBD, localized cytokine neutralization may provide a favorable intestinal environment for microbial recovery. However, oral delivery of therapeutic antibodies is limited by poor stability in the gastrointestinal tract and insufficient local retention. To address this limitation, Li et al. [119] developed a thin-shell hydrogel microcapsule system to encapsulate TNF-α antibodies, thereby improving antibody stability during oral delivery and enabling intestinal release. The double-crosslinked alginate-based shell protected the antibody under weak acidic conditions, with more than 85% of antibodies remaining inside the microcapsules during the first 2 h, whereas only approximately 20% remained after 10 h in PBS. In DSS-induced IBD mice, antibody-loaded microcapsules alleviated colonic inflammation, decreased the DAI score to approximately 2, and reduced TNF-α expression. More importantly for intestinal microecology, this treatment increased microbial richness and promoted the enrichment of inflammation-relieving taxa. At the genus level, *Lachnospiraceae*_UCG-001 and *Lachnospiraceae*_NK4A136 were increased after oral administration of antibody-loaded microcapsules. These *Lachnospiraceae*-related taxa are generally associated with anti-inflammatory effects and SCFA production. Other taxa favorable for intestinal microecological balance, such as *Akkermansia*, Paracaedibacteraceae, and Rumi-UCG-007, also showed enriched abundance. These findings suggest that localized TNF-α neutralization may improve the inflammatory mucosal environment and support the recovery of beneficial anaerobic bacteria.

**Fig. 5:**
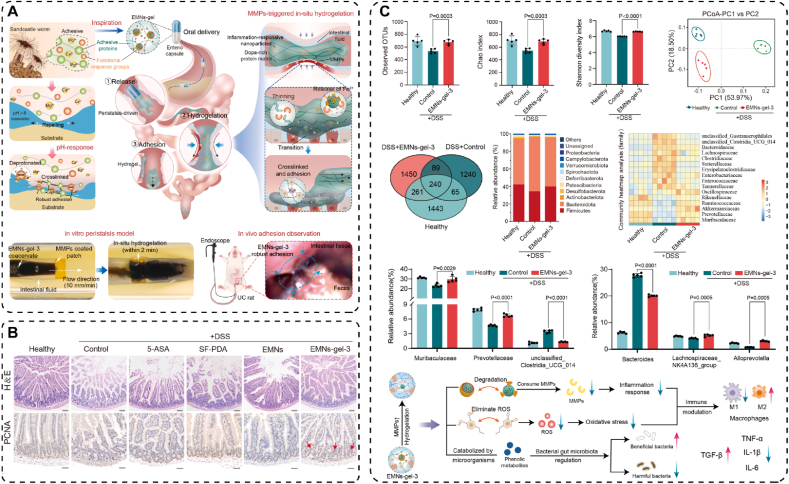
EMNs-gel-3 promotes intestinal repair and microbiota restoration in DSS-induced colitis. (A) Schematic illustration of the design and therapeutic mechanism of EMNs-gel-3, showing oral delivery, peristalsis-driven transport, MMPs-triggered in situ hydrogelation, and selective adhesion at inflamed intestinal sites. (B) Representative H&E and PCNA staining images of colonic tissues from different treatment groups, showing that EMNs-gel-3 alleviated mucosal injury and promoted epithelial regeneration compared with the control and other treatment groups. (C) Gut microbiota analysis showing that EMNs-gel-3 increased bacterial richness and α-diversity, shifted the microbial community closer to the healthy state, increased beneficial taxa such as Muribaculaceae, Prevotellaceae, Lachnospiraceae_NK4A136_group, and Alloprevotella, and reduced potentially harmful taxa including Bacteroides and unclassified_Clostridia_UCG_014. Reproduced with permission [166]. Copyright 2024, Elsevier.

In severe UC, intestinal bleeding, ulcer injury, mucus disruption, and rapid luminal clearance can hinder stable local treatment and delay ecological recovery of the inflamed mucosa. Therefore, hydrogel systems that combine active lesion coverage, prolonged retention, and inflammation-responsive transformation may provide a more suitable environment for microbiota restoration. To address these challenges, Peng et al. [166] developed an inflammation-responsive transformable coacervate system, EMNs-gel, composed of Fe^3+^-loaded inflammation-responsive nanoparticles and a dopa-rich silk fibroin matrix. In inflamed intestinal regions, elevated matrix metalloproteinase activity triggered Fe^3+^ release and induced in situ hydrogel formation. After treatment, 16S rRNA sequencing showed that EMNs-gel increased bacterial richness, as reflected by observed OTUs, and improved α-diversity, including Chao and Shannon indices. PCoA analysis further showed that the microbial profile of the EMNs-gel group shifted closer to that of healthy mice, indicating partial recovery of the DSS-disrupted microbial community. At the phylum level, EMNs-gel increased *Firmicutes*, a change consistent with improved intestinal microbial composition after UC treatment. At the family level, EMNs-gel increased *Muribaculaceae*, a mucin monosaccharide forager associated with healthy gut ecology, and *Prevotellaceae*, which is linked to SCFA production, antioxidant activity, and anti-inflammatory processes. At the genus level, EMNs-gel increased beneficial bacteria such as *Lachnospiraceae_NK4A136_group* and *Alloprevotella*, while reducing potentially harmful taxa such as *Bacteroides* and *unclassified_Clostridia_UCG_014*. These microbiota changes suggest that inflammation-responsive hydrogel formation may help restore intestinal ecological balance by enriching SCFA-associated and mucin-related bacteria while suppressing dysbiosis-associated taxa (Fig. 5).

Compared with studies that mainly report bacterial compositional changes, hydrogel systems that simultaneously evaluate microbial metabolites can provide stronger evidence for functional ecological recovery. In UC, reduced SCFA production is closely associated with impaired barrier function, immune dysregulation, and persistent intestinal inflammation. To achieve coordinated regulation of oxidative stress, macrophage polarization, microbiota composition, and SCFA metabolism, Wang et al. [167] designed an all-in-one oral hydrogel microsphere system, termed BMM/MPs, in which sodium alginate microspheres co-delivered MnO_2_ nanozymes, berberine, and R8-modified magnolol liposomes. Among these components, berberine was included as a gut microbiota-regulating agent, while the hydrogel microspheres protected the bioactive agents during upper GI transit and promoted colon-responsive release. In DSS-induced UC mice, BMM/MPs increased microbial diversity and partially reversed DSS-induced microbial dysbiosis. At the phylum level, Firmicutes showed the most obvious abundance changes among groups. At the genus level, BMM/MPs reduced harmful bacteria such as *Allobaculum*, including *Allobaculum mucolyticum*, which can degrade colonic mucin and aggravate UC. In contrast, BMM/MPs increased beneficial taxa such as *Dubosiella*, *Faecalibaculum*, and *Rikenella*. *Dubosiella* is associated with SCFA production, while *Faecalibaculum* has been linked to regulation of the intestinal mucosal barrier and anti-inflammatory effects. Importantly, GC–MS analysis showed that BMM/MPs increased multiple SCFAs that were reduced in the DSS model, including acetic acid, propionic acid, butyric acid, isobutyric acid, isovaleric acid, and valeric acid. Correlation analysis further linked specific microbial communities with SCFA levels, indicating that hydrogel-mediated microbiota remodeling was accompanied by functional metabolic recovery.

Together, these IBD hydrogel systems highlight treatment-associated microbiota and metabolite remodeling as a distinct outcome beyond conventional inflammation suppression or barrier repair. Their design logic mainly involves localized cytokine neutralization, inflammation-responsive adhesion or gelation, colon-responsive payload release, and combined delivery of microbiota-regulating agents. When accompanied by increased bacterial richness and diversity, enrichment of *Lachnospiraceae*-related taxa, *Akkermansia*, Muribaculaceae, Prevotellaceae, *Dubosiella*, *Faecalibaculum*, or *Rikenella*, reduced potentially harmful taxa such as *Proteobacteria*, *Bacteroides*, or *Allobaculum*, and recovery of SCFA levels, these systems suggest that hydrogel-mediated IBD remission is closely associated with restoration of intestinal microbial composition and metabolic function.

### 3.2 CRC: tumor inhibition, local microenvironment regulation, and microbiota-associated recovery

CRC is one of the most common malignant diseases of the lower-GI tract. Conventional therapies, including chemotherapy and surgical resection, have achieved important clinical progress. However, they remain limited by systemic toxicity, poor treatment selectivity, chemoresistance, and insufficient local drug accumulation [168,169]. Oral hydrogel systems have attracted increasing attention in CRC treatment. They can protect antitumor agents from premature degradation, improve intestinal retention, enable localized or stimuli-responsive release, and reduce systemic exposure. In addition to direct tumor inhibition, some hydrogel systems can remodel the tumor microenvironment, regulate local immune responses, and influence microbiota-associated metabolic pathways involved in CRC progression.

#### 3.2.1 Localized antitumor and microenvironment-regulating hydrogel systems

Localized tumor inhibition and tumor microenvironment regulation are major directions for oral hydrogel systems in CRC therapy. Compared with conventional chemotherapy, oral hydrogel platforms can protect antitumor agents from premature degradation or absorption in the upper GI tract, prolong intestinal retention, and enable localized release in the colorectum. By integrating pH-responsive swelling, colonic enzyme-mediated degradation, tumor-cell targeting ligands, bacterial metabolic activity, and nanoparticle-based combination therapy, these systems can improve local drug exposure while reducing systemic toxicity. In this category, the main design logic is to connect hydrogel-mediated GI protection and colon-targeted release with tumor-cell killing, chemosensitization, anti-metastatic effects, or tumor microenvironment regulation.

Pain management and insufficient colorectal drug accumulation are two important challenges in CRC treatment [170,171]. Conventional chemotherapy often fails to maintain effective drug concentrations in the colorectum because drugs may be prematurely released or absorbed in the stomach and small intestine, while cancer-related pain also requires additional symptom control. To address these issues, Sheng et al. [172] constructed a dual-drug delivery system based on alginate and sodium carboxymethyl cellulose (CMC) hydrogels crosslinked with Ca^2+^ for simultaneous chemotherapy and pain relief. In this system, methotrexate-loaded CaCO_3_ microspheres and aspirin were co-entrapped within the Alg/CMC hydrogel network. The hydrogel layer protected MTX from premature release, while the combined pH-dependent swelling of Alg/CMC and pH-sensitive decomposition of CaCO_3_ enabled region-specific release. The system showed limited drug release in simulated gastric fluid during the first 2 h. In simulated GI fluids, aspirin release reached 57.2% at 8 h, supporting intestinal pain-relief delivery, whereas MTX release increased to 47.6% at 24 h, indicating enhanced colorectal chemotherapy delivery. At the cellular level, the blank hydrogel carrier showed good biocompatibility, while the drug-loaded system reduced SW480 colon cancer cell viability to less than 10% at 128 μg mL^−1^.

Chemoresistance and insufficient tumor-cell-specific drug uptake are also major obstacles in CRC therapy [173,174]. CD98 is highly expressed in colon cancer tissues and is associated with tumor progression, making it both a therapeutic target and a receptor for targeted delivery. However, effective CRC therapy requires simultaneous delivery of gene-regulatory agents and chemotherapeutic drugs to the same tumor cells. To address this need, Xiao et al. [130] developed a chitosan/alginate hydrogel-embedded nanoparticle system for combined CD98 silencing and chemotherapy. In this system, CD98 siRNA and camptothecin were co-loaded into CD98 Fab’-functionalized PLGA nanoparticles, which were then embedded in a chitosan/alginate hydrogel. The hydrogel protected the nanoparticles during GI transit and enabled their release in the colonic lumen, while Fab’ functionalization promoted cellular uptake by CD98-overexpressing colon cancer cells. The hydrogel minimized premature leakage during GI transit, with less than 4.09% CPT released from the NP-embedded hydrogel within 24 h under tested GI conditions. After release from the hydrogel, Fab’-functionalized nanoparticles showed enhanced uptake by Colon-26 cells, with fluorescence intensity 1.9-, 2.1-, and 1.8-fold higher than PEGylated nanoparticles after 1, 3, and 5 h, respectively. Functionally, Fab’-siCD98-NPs achieved up to 86.9% CD98 knockdown after 24 h, and co-delivery of siCD98 and CPT enhanced apoptosis, inhibited migration, and improved chemosensitization. In an orthotopic colon tumor model, oral Fab’-siCD98/CPT-NP/hydrogel treatment achieved a tumor inhibition rate of 63.8%, which was higher than Fab’-CPT-NPs and PEG-siCD98/CPT-NPs alone (37.6% and 33.8%, respectively). This system linked hydrogel-mediated colon-level delivery with Fab’-mediated tumor-cell targeting, CD98 silencing, CPT chemotherapy, apoptosis induction, and inhibition of CRC progression.

Another limitation of oral nanomedicine for CRC is that nanoparticles can be rapidly cleared from the GI tract or retained in the upper GI tract, resulting in insufficient accumulation at colonic tumors [175]. In addition, monotherapy often shows limited efficacy against local tumor growth and metastasis. To achieve colon-localized and tumor-cell-targeted combination therapy, Lu et al. [176] developed microfluidized dextran microgels loaded with cisplatin/SPION-containing lipid nanoparticles for oral local colon cancer treatment. In this design, crosslinked dextran microgels reduced upper-GI retention of lipid nanoparticles and protected the therapeutic bioactive agents during oral delivery. After reaching the colon, dextranase-mediated degradation of the microgels released lipid nanoparticles and exposed folic acid residues on their surfaces, thereby promoting uptake by folate receptor-overexpressing colon cancer cells. The formulation minimized premature drug leakage, with cisplatin leakage below 5% in simulated gastric fluid for 1 h and below 10% in simulated intestinal fluid for 4 h. After dextranase pretreatment, cellular uptake of folate-modified DFCNPs was approximately four-fold higher than that of non-targeted DTCNPs in CT26 cells, and chemo/magnetothermal combination therapy produced the strongest cytotoxic effect. In orthotopic colon cancer-bearing mice, more than 50% of the fluorescence signal accumulated in the colon at 10 h after oral administration of DFCNPs@MGs. Therapeutically, DFCNPs@MGs combined with HFMF treatment reduced tumor weight approximately tenfold compared with the PBS control, whereas unencapsulated DFCNPs and folate-free DTCNPs@MGs produced only 3.3-fold and 4.1-fold reductions, respectively. H&E and caspase-3 staining further indicated reduced tumor invasion and enhanced tumor-cell apoptosis, and the treatment also decreased peritoneal carcinomatosis. This example shows that dextran microgels can provide colon-level enzymatic targeting, while folate-modified lipid nanoparticles and SPIONs enable tumor-cell targeting, chemotherapy, magnetothermal ablation, and metastasis suppression.

**Fig. 6:**
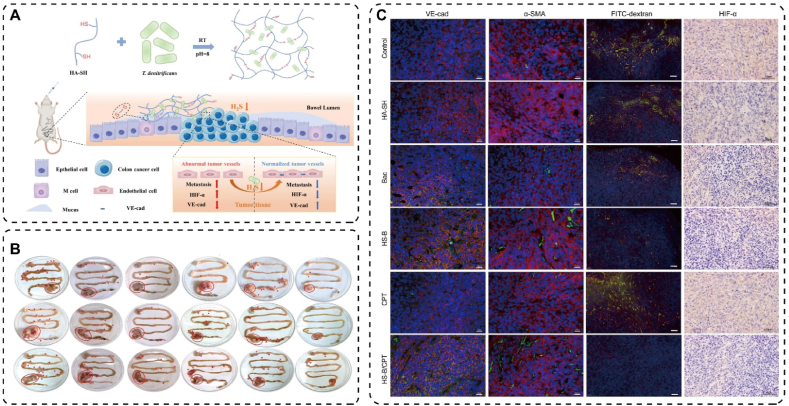
Colon-targeted bacterial hydrogel for tumor vascular normalization and improved chemotherapy. (A) Schematic illustration of the preparation of the HA-SH-based bacterial hydrogel and its proposed therapeutic mechanism after oral administration, showing colonic adhesion, bacterial H_2_S scavenging, tumor vascular normalization, and improved chemotherapy efficacy. (B) Representative images of intestinal tracts from different treatment groups, showing that HS-B/CPT reduced tumor burden and metastasis more effectively than the control and single-treatment groups. (C) Representative immunofluorescence and immunohistochemical images of tumor tissues stained for VE-cad, α-SMA, FITC-dextran, and HIF-α, demonstrating that HS-B/CPT promoted tumor vascular normalization, reduced vascular permeability, and alleviated tumor hypoxia. Reproduced with permission [180]. Copyright 2023, Elsevier.

Besides direct drug delivery and tumor-cell targeting, regulation of tumor-associated metabolic cues represents another important strategy for CRC microenvironment remodeling [177]. Endogenous H_2_S is enriched in the colon cancer microenvironment and contributes to tumor proliferation, angiogenesis, abnormal tumor vessels, and reduced chemotherapy penetration. However, direct oral administration of H_2_S-consuming bacteria is limited by bacterial loss of viability in the acidic GI tract and potential biosafety concerns [178,179]. To overcome these limitations, Li et al. [180] developed a colon-targeted bacterial hydrogel based on sulfhydryl-modified HA (HA-SH), in which *Thiobacillus denitrificans* and camptothecin were loaded to construct HS-BAC/CPT. In this system, HA-SH promoted colonic adhesion through disulfide bonding with mucin, while the hydrogel confined bacteria, protected their viability during GI transit, and prevented bacterial escape. The encapsulated *T. denitrificans* was used to consume excessive H_2_S in the CRC microenvironment. *In vitro*, *T. denitrificans* rapidly consumed H_2_S in a concentration-dependent manner; 10^7^ units mL^−1^ bacteria almost completely consumed 7 mM NaHS within 1 h. HA-SH hydrogel protected bacteria from simulated gastric fluid, reducing the bacterial death ratio from more than 80% in SGF to less than 20% in the HS-BAC/SGF group. In cell experiments, NaHS increased CT26 cell viability to more than 150%, whereas bacteria-containing treatment reduced H_2_S content and decreased NaHS-enhanced cell viability toward the control level. When combined with CPT, HS-BAC/CPT reduced CT26 cell viability to approximately 80% under H_2_S -rich conditions, compared with approximately 120% after CPT alone. In an colon cancer mouse model, oral HS-BAC/CPT reduced tumor bioluminescence, decreased H_2_S levels in tumors and feces, lowered tumor metastasis, and increased tumor-cell apoptosis. Mechanistically, H_2_S consumption promoted tumor vascular normalization, as reflected by improved VE-cadherin expression, reduced hypoxia-related signaling, and enhanced CPT delivery. Therefore, this bacterial hydrogel did not merely deliver chemotherapy but regulated a tumor-associated metabolic microenvironment, linking colonic adhesion, bacterial H_2_S scavenging, vascular normalization, improved chemotherapy penetration, and CRC suppression (Fig. 6).

Together, these localized antitumor and microenvironment-regulating hydrogel systems improve CRC therapy by connecting lower-GI delivery with tumor inhibition and local microenvironment remodeling. Their design logic mainly involves upper-GI protection, colorectal pH- or enzyme-responsive release, tumor-cell-targeted uptake, sustained local drug exposure, and combination therapy. When accompanied by higher tumor inhibition rates, reduced tumor burden, enhanced apoptosis, suppressed migration or metastasis, and improved chemotherapy penetration, these systems indicate that oral hydrogels can function as localized CRC treatment platforms rather than passive drug carriers. In particular, systems that regulate tumor-associated cues such as H_2_S further expand hydrogel therapy from drug delivery toward metabolic microenvironment modulation, vascular normalization, and chemosensitization.

#### 3.2.2 Treatment-associated microbiota and metabolite remodeling

CRC progression is closely associated with dysbiosis of the intestinal and intratumoral microbiota, as well as abnormal microbial metabolite profiles. Several CRC-oriented hydrogel systems have reported that antitumor effects are accompanied by changes in microbial richness, beneficial bacterial genera, potentially harmful taxa, SCFA-producing bacteria, or tumor-associated microbial metabolites [181,182]. These findings suggest that hydrogel-mediated CRC therapy may influence not only drug localization and tumor inhibition but also the intestinal microbial and metabolic environment associated with tumor progression.

The depletion of beneficial SCFA-producing bacteria and enrichment of tumor-associated harmful taxa are common microbial features of CRC. To combine localized chemotherapy with microbiota-associated ecological recovery, Li et al. [183] developed an orally administered inulin-based hydrogel containing oxaliplatin-loaded hollow MnO_2_ nanoparticles, termed Oxa@HMI. In this system, the inulin matrix protected the nanomedicine during upper-GI transit, prolonged intestinal retention, and could be degraded by beneficial colonic bacteria to generate SCFAs. Meanwhile, the exposed MnO_2_-based nanocarrier responded to the acidic tumor microenvironment, producing Mn^2+^ and O_2_, increasing ROS generation, and enhancing Oxa-mediated tumor killing. In orthotopic colorectal tumor-bearing mice, Oxa@HMI markedly reduced tumor bioluminescence, tumor size, and tumor weight compared with PBS, free Oxa, and Oxa@HM groups, while Ki-67 staining decreased and TUNEL-positive apoptotic signals increased. Importantly, 16S rRNA sequencing showed that Oxa@HMI increased the richness and diversity of the intestinal microbiota, with Chao-1 and Shannon indices shifting closer to those of healthy controls. At the phylum level, Oxa@HMI increased Firmicutes and reduced Bacteroidota; at the genus level, it increased beneficial SCFA-associated bacteria, including *Lactobacillus*, *Lachnospiraceae*, and *Roseburia*, while reducing harmful CRC-associated bacteria such as *Alistipes*, *Helicobacter*, and Clostridia. Similar remodeling was also observed in the intratumoral microbiota, where Oxa@HMI increased beneficial *Lactobacillus*, *Lachnospiraceae*, and *Akkermansia*, while reducing Bacteroidota, *Alistipes*, *Helicobacter*, and *Escherichia*. These microbiota changes were further associated with increased CD8^+^ and CD4^+^ T-cell infiltration, DC maturation, M1 macrophage polarization, decreased MDSCs, and enhanced IFN-γ secretion, suggesting that inulin hydrogel-assisted chemotherapy may promote CRC suppression through coordinated microbiota–SCFA–immune remodeling.

The connection between hydrogel-mediated microbial fermentation, SCFA production, and antitumor immunity has also been demonstrated in immune-checkpoint therapy [184]. Because beneficial commensal microorganisms such as *Akkermansia*, *Lactobacillus*, and *Roseburia* mainly reside in the colon and are associated with improved immune-checkpoint blockade responses, Han et al. [131] developed an orally administered colon-retentive inulin gel to modulate the gut microbiome in situ. Compared with free inulin, the inulin gel remained longer in the colon and cecum, thereby increasing cumulative colonic exposure to fermentable inulin. In CT26 tumor-bearing mice, inulin gel plus anti-PD-1 therapy induced a distinct shift in gut microbial community structure, increased the relative abundance of *Akkermansia*, and showed a trend toward increased *Roseburia*, while reducing LPS-producing *Oscillibacter*. This microbial remodeling was accompanied by increased fecal SCFAs, including acetate, propionate, and butyrate. Mechanistically, microbial fermentation-derived SCFAs triggered GPR43-dependent antitumor CD8^+^ T-cell responses; antibiotic treatment abolished the antitumor effect, whereas GPR43-deficient mice lost the ability to respond effectively to inulin gel plus anti-PD-1 therapy. Therapeutically, inulin gel plus anti-PD-1 markedly delayed tumor growth and doubled the complete regression rate compared with free inulin plus anti-PD-1. It also increased AH1-specific CD8^+^ T cells, IFN-γ^+^ CD8^+^ T-cell responses, intratumoral CD8^+^ and CD4^+^ T-cell infiltration, CD86^+^ CD11c^+^ dendritic cells, and stem-like Tcf1^+^PD-1^+^CD8^+^ T cells. This study demonstrates that oral hydrogel-induced microbiota and SCFA remodeling can support systemic antitumor immunity and potentiate immune-checkpoint therapy.

Together, these studies show that CRC-oriented hydrogel therapy can be accompanied by remodeling of intestinal and intratumoral microbial ecosystems and their functional metabolites. The most relevant changes include increased microbial richness and diversity, enrichment of SCFA-associated beneficial bacteria such as *Lactobacillus*, *Roseburia*, *Lachnospiraceae*, *Akkermansia*, and *Ruminococcus*, reduction of potentially harmful taxa such as Bacteroidota, *Alistipes*, *Helicobacter*, Clostridia, *Escherichia–Shigella*, and *Oscillibacter*, and increased SCFA production. These outcomes suggest that hydrogel-mediated CRC treatment may act through a broader microbiota–SCFA–immune axis, rather than through localized drug delivery alone.

### 3.3 IBS and gut–brain axis-related disorders: symptom relief and microbiota–metabolite interactions

The gut–brain axis provides a bidirectional communication route between the gastrointestinal tract and the central nervous system through neural, immune, endocrine, and microbial signaling. Dysregulation of this axis has been implicated in functional gastrointestinal disorders, such as IBS, as well as extraintestinal symptoms including sleep disturbance, anxiety, and depression. Alterations in gut microbiota can influence intestinal permeability, systemic inflammation, microbial metabolite production, neurotransmitter metabolism, and neuroimmune responses. These changes may affect both intestinal symptoms and neurological functions [185,186].

#### 3.3.1 Hydrogel systems for gut–brain axis-related symptom relief

High-altitude sleep disturbance is one of the most common neurological symptoms of acute mountain sickness (AMS), characterized by sleep fragmentation, difficulty falling asleep, early awakening, vivid dreams, and impairments in memory and behavior [[187], [188], [189]]. Recent studies have shown that the gut–brain axis plays an important role in altitude-induced sleep dysfunction. High-altitude hypoxia can disrupt gut microbiota composition, impair intestinal homeostasis, promote systemic inflammation, and alter microbiota-mediated neuroimmune communication, thereby aggravating sleep and cognitive dysfunction [190,191]. Therefore, oral delivery systems that can regulate gut microbiota, preserve intestinal barrier conditions, and reduce inflammatory signaling may provide a non-invasive strategy for improving gut–brain axis-related symptoms.

**Fig. 7:**
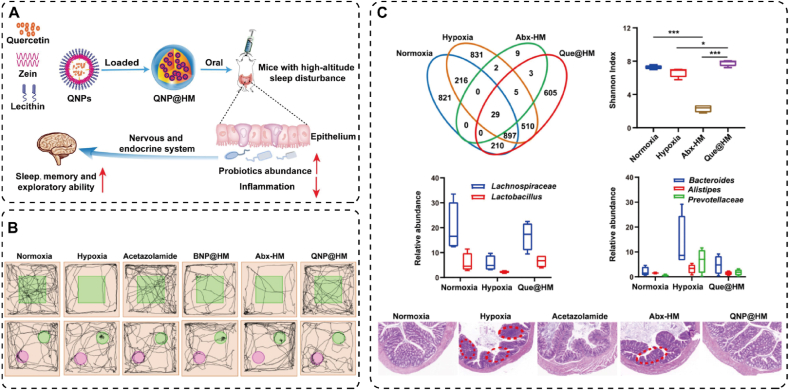
QNP@HM hydrogel microspheres for colon-targeted quercetin delivery and gut–brain axis regulation. (A) Schematic illustration of QNP@HM preparation and oral administration, showing quercetin-loaded zein nanoparticles embedded in hydrogel microspheres to improve intestinal delivery and regulate gut microbiota-related sleep, memory, and exploratory behaviors. (B) Behavioral assessment showing that QNP@HM improved spontaneous activity and short-term memory in mice with high-altitude sleep disturbance compared with the hypoxia group. (C) Gut microbiota and colon histology analyses showing that QNP@HM restored microbial diversity, increased probiotic-associated taxa such as Lachnospiraceae and Lactobacillus, reduced harmful bacteria, and alleviated hypoxia-induced colonic injury. Reproduced with permission [134]. Copyright 2024, Elsevier.

Quercetin has antioxidant, anti-inflammatory, and microbiota-regulating activities, and is considered a potential candidate for preventing high-altitude sleep disturbance. However, its poor water solubility, low dispersibility, and limited oral bioavailability restrict its biological function in the gastrointestinal tract. To overcome these limitations and enhance colon-targeted delivery, Wu et al. [134] developed calcium alginate-based hydrogel microspheres encapsulating quercetin nanoparticles, termed QNP@HMs. In this system, quercetin was first formulated into zein-based nanoparticles using an antisolvent method and then embedded into calcium alginate hydrogel microspheres by electrostatic spraying. The microspheres were further coated with thiolated chitosan, which could interact with colonic mucin and improve intestinal retention. Compared with free quercetin, QNP@HMs reduced premature release in simulated gastric and intestinal fluids and mainly released quercetin in simulated colonic fluid. Free quercetin showed rapid release in the early simulated GI process, with cumulative release reaching approximately 68% within 0–8 h, whereas QNP@HMs remained relatively stable in gastric and intestinal conditions and released the payload mainly after entering the simulated colon environment **(**Fig. 7**)**.

In a mouse model of high-altitude sleep disturbance, QNP@HMs showed clear symptom-relieving effects. Hypoxia markedly shortened sleep duration compared with normoxia, whereas QNP@HMs prolonged sleep duration to a level close to the acetazolamide-treated group. In the open-field test, hypoxia reduced spontaneous locomotor activity and exploration of the central area, while QNP@HMs increased total movement distance, central-area entry frequency, and central-area exploration time. In the novel object recognition test, QNP@HMs also improved the recognition index, indicating partial recovery of short-term memory. Notably, blank hydrogel microspheres and QNP@HMs administered after long-term antibiotic treatment did not produce comparable behavioral improvement, suggesting that the therapeutic effect was closely associated with gut microbiota regulation.

QNP@HMs also alleviated hypoxia-associated inflammatory and physiological changes. High-altitude hypoxia increased peripheral blood WBC, RBC, HGB, and HCT levels and elevated inflammatory mediators, including TNF-α and iNOS. After QNP@HMs treatment, these abnormal blood parameters shifted toward the healthy range, while TNF-α and iNOS levels were reduced compared with the hypoxia group. The antibiotic-treated QNP@HM group showed little improvement, further supporting the contribution of gut microbiota to the anti-inflammatory and symptom-relieving effects of this hydrogel system.

Gut microbiota analysis provided additional evidence for microbiota-dependent regulation of high-altitude sleep disturbance. Hypoxia reduced microbial richness and diversity, increased the abundance of potentially harmful bacteria such as *Bacteroides*, *Alistipes*, and *Prevotellaceae*, and decreased beneficial bacteria including *Lactobacillus* and *Lachnospira*. QNP@HMs reversed these dysbiotic changes by increasing microbial diversity, enriching beneficial taxa such as *Lactobacillus* and *Lachnospira*, and reducing potentially harmful bacteria. Since *Lactobacillus* and *Lachnospira* are associated with SCFA production, these microbial changes suggest that QNP@HMs may improve sleep-related neurological symptoms partly through microbiota-associated metabolic regulation. Histological analysis further showed that QNP@HMs alleviated hypoxia-induced colonic injury, preserved crypts and goblet cells, reduced inflammatory infiltration, and maintained intestinal barrier conditions. However, after antibiotic-induced gut microbiota depletion, QNP@HMs failed to exert comparable therapeutic effects, indicating that intact gut microbiota was necessary for its gut–brain axis-related efficac.

Together, this hydrogel microsphere system demonstrates how oral hydrogels can be used for gut–brain axis-related symptom relief by connecting colon-targeted release, microbiota regulation, intestinal barrier protection, systemic inflammation reduction, and behavioral improvement. In this category, the design logic mainly involves improving the gastrointestinal stability and colonic retention of poorly soluble bioactive compounds, restoring beneficial microbiota such as *Lactobacillus* and Lachnospira, reducing harmful taxa such as *Bacteroides* and *Alistipes*, and preserving intestinal mucosal integrity. These effects support the role of hydrogel-mediated intestinal regulation in improving extraintestinal neurological symptoms associated with gut–brain axis dysfunction.

#### 3.3.2 Treatment-associated microbiota and metabolite remodeling in hydrogel-mediated gut–brain regulation

Gut–brain axis dysregulation is closely associated with mental health disorders, including anxiety and depression. In IBD, chronic intestinal inflammation and microbial dysbiosis can disrupt epithelial barrier integrity, increase intestinal permeability, and promote the leakage of microbial products such as lipopolysaccharide (LPS) into systemic circulation [192,193]. These gut-derived inflammatory signals may further reach the central nervous system, especially the hippocampus, thereby inducing neuroinflammation, impairing neuroplasticity, and contributing to anxiety- and depression-like behaviors. Therefore, hydrogel systems capable of improving intestinal retention, restoring barrier integrity, and regulating microbiota–metabolite interactions may provide a non-invasive strategy for modulating gut-derived neuroimmune signals.

IBD-associated anxiety and depression are difficult to treat because conventional IBD therapies mainly focus on intestinal inflammation, while psychiatric symptoms may persist through microbiota–gut–brain axis dysfunction. Rhein has anti-inflammatory, antioxidant, microbiota-regulating, and neuroprotective activities, but its poor water solubility, limited intestinal retention, and insufficient oral bioavailability restrict its therapeutic efficacy. Meanwhile, *Spirulina platensis* (SP), a natural microalga with nutritional and microbiota-regulating properties, can help maintain intestinal microbial homeostasis but still requires improved intestinal retention for efficient oral therapy. To address these limitations, Zhong et al. [194] developed an oral microalgae-based hydrogel system, SP@Rh-gel, by integrating *Spirulina platensis* with self-assembled rhein hydrogel for the synergistic treatment of IBD and IBD-associated anxiety and depression. This system improved rhein solubility, enabled pH-responsive intestinal release, and enhanced gastrointestinal retention. Compared with free SP or rhein solution, SP@Rh-gel produced stronger and more prolonged gastrointestinal fluorescence signals after oral administration, with SP and rhein-related signals increasing by 2.4-fold and 3.3-fold, respectively. The fluorescence signal remained detectable even at 34 h post-administration, indicating prolonged intestinal residence. SP@Rh-gel also showed minimal release under gastric conditions but sustained rhein release under intestinal conditions, reaching approximately 100% release within 72 h.

In DSS-induced chronic colitis mice, SP@Rh-gel alleviated intestinal inflammation and improved colitis symptoms more effectively than free SP, rhein solution, or rhein hydrogel alone. Colon length was restored from 6.33 ± 0.53 cm in the DSS group to 8.02 ± 0.42 cm after SP@Rh-gel treatment, indicating reduced colonic inflammation. Histological analysis showed that SP@Rh-gel preserved epithelial structure and reduced crypt destruction, while immunohistochemical and RT-qPCR analyses showed lower TNF-α, IL-6, and IL-1β expression in colon tissues. More importantly for gut–brain axis regulation, SP@Rh-gel restored epithelial barrier integrity by upregulating tight junction proteins, including Claudin-1, Occludin-1, and ZO-1, and reduced plasma zonulin and LPS leakage. These results indicate that hydrogel-enhanced intestinal retention and local release helped repair the intestinal barrier and limit the systemic entry of microbial inflammatory products.

Behavioral analyses further demonstrated that SP@Rh-gel improved IBD-associated anxiety- and depression-like symptoms. In the open field test and elevated plus maze, SP@Rh-gel increased central-zone residence time, open-arm time, and open-arm crossing frequency compared with DSS-treated mice. In the forced swimming and tail suspension tests, SP@Rh-gel reduced immobility time, suggesting relief of depression-like behavior. At the CNS level, SP@Rh-gel reduced plasma and hippocampal LPS levels, decreased hippocampal pro-inflammatory cytokines, inhibited NLRP3 inflammasome and NF-κB signaling, and suppressed microglial activation. It also promoted microglial polarization from a pro-inflammatory M1-like phenotype toward an M2-like phenotype, increased hippocampal neurogenesis, and improved neuronal survival and neuroplasticity. These results suggest that intestinal hydrogel-mediated barrier repair and reduced microbial product leakage can be connected to hippocampal neuroimmune protection.

**Fig. 8:**
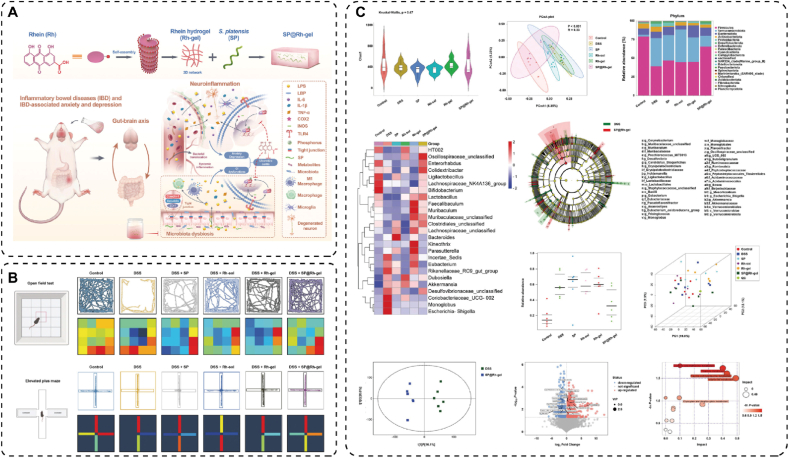
SP@Rh-gel hydrogel for microbiota–gut–brain axis regulation in IBD-associated anxiety and depression. (A) Schematic illustration of SP@Rh-gel preparation and therapeutic mechanism, showing self-assembled rhein hydrogel loaded with *Spirulina platensis* for oral treatment of IBD and IBD-associated anxiety/depression through microbiota–gut–brain axis regulation. (B) Behavioral analyses, including open field and elevated plus maze tests, showing that SP@Rh-gel alleviated DSS-induced anxiety-like behaviors compared with untreated IBD mice. (C) Gut microbiota and fecal metabolomics analyses showing that SP@Rh-gel reshaped DSS-disrupted microbial community structure, reduced gram-negative bacteria, and regulated inflammation- and neuroprotection-related metabolites and pathways. Reproduced with permission [194]. Copyright 2024, Wiley-VCH.

Microbiota and metabolomic analyses further supported the role of microbiota–metabolite interactions in SP@Rh-gel-mediated gut–brain regulation. Although alpha diversity indexes showed no major differences among groups, beta diversity analysis revealed that SP@Rh-gel shifted the microbial community structure away from the DSS group and closer to the healthy state. At the genus level, SP@Rh-gel increased SCFA-associated and anti-inflammatory bacteria, including *Ligilactobacillus*, *Faecalibacterium*, *Muribaculum*, *Anaerostipes*, and *Phascolarctobacterium*, while reducing the predicted abundance of gram-negative bacteria. Fecal metabolomics showed that DSS altered 130 metabolites compared with the control group, and these changes were partially restored after SP@Rh-gel treatment. Differential metabolites included indoleacrylic acid, indole-3-acetic acid, thiamine, and isobutyric acid, while KEGG enrichment highlighted thiamine metabolism, vitamin B6 metabolism, and taurine/hypotaurine metabolism. Correlation analysis further linked thiamine and indoleacrylic acid with beneficial bacteria such as *Ligilactobacillus*, *Anaerostipes*, and *Muribaculum*. These findings suggest that SP@Rh-gel alleviated IBD-associated anxiety- and depression-like behaviors by coordinating intestinal retention, barrier repair, microbiota remodeling, microbial metabolite regulation, systemic inflammation reduction, and hippocampal neuroinflammation suppression **(**Fig. 8**)**.

Together, this microalgae-based hydrogel system demonstrates how oral hydrogels can regulate gut–brain axis-related disorders through treatment-associated microbiota and metabolite remodeling. Its design logic mainly involves improving intestinal retention of bioactive microalgae and poorly soluble neuroprotective compounds, restoring epithelial barrier integrity, reducing LPS leakage, enriching SCFA-associated beneficial bacteria, regulating indole-, vitamin-, and taurine-related metabolites, and suppressing hippocampal neuroinflammation. Compared with hydrogel systems that mainly relieve intestinal symptoms, SP@Rh-gel provides a representative example in which hydrogel-mediated intestinal ecological restoration is linked to extraintestinal neurological improvement through the microbiota–gut–brain axis.

### 3.4 Gut microbiota-associated metabolic disorders: indirect regulation through microbiota–metabolite pathways

Metabolic disorders, such as obesity and type 2 diabetes, are not lower-GI disorders in a strict anatomical sense. However, their occurrence and progression are closely associated with lower-GI microbial ecology, microbial metabolites, intestinal barrier dysfunction, and metabolic endotoxemia. Increasing evidence indicates that dysbiosis of the gut microbiota can impair SCFA production, promote the expansion of endotoxin-producing bacteria, disrupt epithelial barrier integrity, and aggravate systemic low-grade inflammation, thereby contributing to insulin resistance and metabolic dysfunction [195,196]. Therefore, in the context of lower-GI-targeted hydrogel systems, metabolic disorders are discussed here only when their therapeutic effects are closely related to gut microbiota remodeling, microbiota-derived metabolites, intestinal barrier repair, or endotoxemia reduction. They are not discussed from the perspective of conventional systemic glucose control alone.

#### 3.4.1 Barrier-repairing hydrogel systems for endotoxemia reduction and metabolic improvement

Intestinal barrier dysfunction is closely associated with obesity, diabetes, non-alcoholic Fatty Liver Disease (NAFLD), and other metabolic disorders. Under high-fat diet conditions, disruption of tight junctions and mucus protection can promote the translocation of microbial products, especially LPS, into the bloodstream [197,198]. This process contributes to metabolic endotoxemia, adipose tissue inflammation, insulin resistance, and lipid metabolic disorder. Therefore, hydrogel systems that reinforce the epithelial–luminal interface and reduce endotoxin leakage may indirectly improve systemic metabolic homeostasis.

To address these interconnected abnormalities, Jiang et al. [199] developed an orally administered β-glucan-based superabsorbent hydrogel (βC-MA hydrogel) for obesity-associated metabolic disorders [200]. This hydrogel was designed to swell in the gastrointestinal tract, delay gastric emptying, reduce nutrient digestion and absorption, and improve the intestinal microenvironment. In high-fat diet-induced obese mice, βC-MA hydrogel reduced body weight gain with an efficacy comparable to semaglutide and superior to β-glucan or carboxymethylcellulose alone. In addition to its gastric expansion effect, βC-MA hydrogel improved intestinal barrier function, as shown by increased expression of claudin-1, ZO-1, and MUC2, together with reduced serum LPS and lower intestinal inflammatory cytokines, including TNF-α and IL-6. Gut microbiota analysis showed that βC-MA hydrogel enriched obesity-negative bacteria, including *Akkermansia*, n*orank_f__Muribaculaceae*, and *Faecalibaculum*, while increasing fecal SCFA levels, including acetate, propionate, and butyrate. These changes were accompanied by improved glucose and lipid metabolism, reduced fat accumulation, improved HOMA-IR, and alleviated hepatic lipid deposition. This example suggests that the metabolic benefit of βC-MA hydrogel is not limited to appetite control, but is also associated with barrier repair, reduced endotoxemia, beneficial microbiota enrichment, and SCFA recovery.

Mucus-layer impairment and reduced abundance of mucus-associated bacteria are also important features of obesity- and NAFLD-related intestinal dysfunction [201,202]. In this context, Silvestri et al. [139] developed a biomimetic oral superabsorbent hydrogel (OSH) that mimics the mechanical and compositional features of fiber-rich raw vegetables and functions as a gut-protective “dynamic exoskeleton”. Unlike conventional prebiotic fibers, OSH was designed as a crosslinked cellulose-based hydrogel with high water content, elasticity, and structural stability in the gastrointestinal lumen. In high-fat diet-fed mice, OSH protected intestinal barrier function by reducing circulating LPS, enhancing MUC2 expression in the colon, and preserving ileal ZO-1 expression. At the metabolic level, OSH reduced body weight gain, adipose tissue expansion, fasting glucose, insulin levels, circulating LDL, and hepatic steatosis. Importantly, metagenomic analysis showed that OSH partially reversed high-fat diet-induced dysbiosis, restored the Firmicutes/Bacteroidetes ratio, and specifically expanded *Akkermansia muciniphila*. In short-term experiments, *A. muciniphila* abundance increased 5.7-fold and 12.0-fold after 2% and 4% OSH supplementation, respectively, compared with the HFHCC diet alone. *In vitro* experiments further showed that OSH directly promoted *A. muciniphila* growth, whereas non-crosslinked CMC, inulin, psyllium, or a synthetic gel with similar elasticity did not reproduce this effect. These results indicate that both the chemical composition and 3D mechanical structure of OSH are required to support *A. muciniphila* expansion and metabolic improvement.

Together, these barrier-repairing hydrogel systems improve metabolic dysfunction mainly by targeting the intestinal barrier–microbiota–endotoxemia axis. Their design logic involves gastrointestinal swelling, mucus-layer mimicry, tight junction preservation, MUC2 restoration, *A. muciniphila* enrichment, SCFA recovery, and reduced LPS translocation. Compared with conventional glucose- or appetite-centered strategies, these examples show that oral hydrogels can indirectly regulate systemic metabolism by rebuilding intestinal barrier function and reshaping the lower-GI microbial environment.

#### 3.4.2 Carrier-associated microbiota remodeling and insulin sensitivity

Some oral hydrogel systems were originally developed for oral insulin delivery, with their primary design focused on insulin protection, pH-sensitive release, or enhanced intestinal absorption. However, when the carrier itself influences gut microbial composition and metabolic homeostasis during long-term administration, such systems can also be discussed from a microbiota-associated metabolic perspective. Therefore, this subsection does not emphasize conventional insulin delivery efficiency, but focuses on carrier-associated microbiota remodeling and its possible contribution to insulin sensitivity.

Oral insulin therapy is limited by enzymatic degradation, gastric acidity, poor intestinal permeability, and insufficient absorption across the intestinal epithelium. To overcome these delivery barriers, Ren et al. [203] developed a microalgae-derived natural hydrogel carrier, which possesses good biocompatibility, pH-responsive behavior, and the potential to interact with the intestinal microenvironment. In this context, the authors developed a *Chlorella vulgaris*-based oral insulin delivery platform, CV@INS@ALG, in which insulin was incorporated into an alginate-coated microalgal carrier. This system protected insulin from gastric degradation and enabled pH-sensitive intestinal release. Under acidic gastric conditions, the hydrogel network retained insulin, whereas the increased intestinal pH promoted hydrogel swelling and insulin release. CV@INS@ALG further facilitated insulin absorption through direct release from the delivery matrix and uptake by M cells via macrophage-mediated endocytosis. In STZ-induced type 1 diabetic mice, CV@INS@ALG achieved glycemic control comparable to injected insulin without causing apparent intestinal damage.

**Fig. 9:**
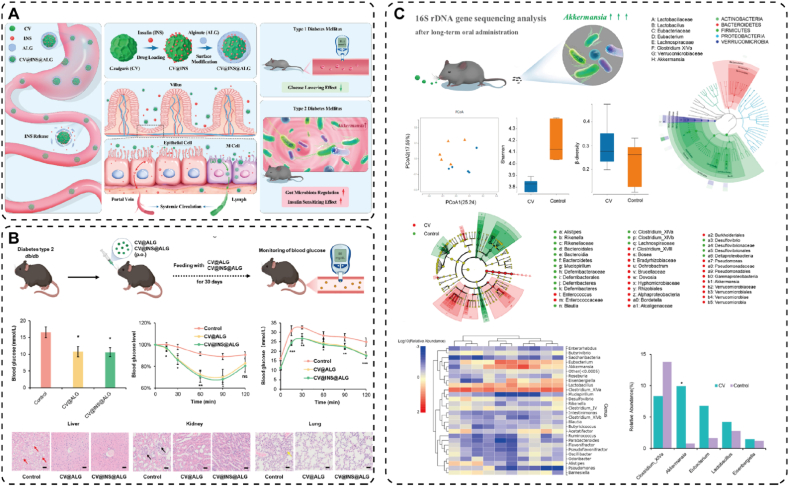
Microalgae-derived pH-responsive hydrogel for oral insulin delivery and microbiota-associated metabolic regulation. (A) Schematic illustration of CV@INS@ALG construction and oral insulin delivery, showing insulin loading into *Chlorella vulgaris*, alginate coating, pH-responsive intestinal insulin release, and insulin absorption through direct epithelial uptake or M cell/macrophage-mediated transport. (B) Long-term oral administration of the CV@ALG carrier modulated the gut microbiota in db/db type 2 diabetic mice, as shown by 16S rDNA sequencing, with altered microbial community structure and increased abundance of beneficial taxa such as *Akkermansia*, which was associated with improved insulin sensitivity. Reproduced with permission [203]. Copyright 2023, American Chemical Society.

From the perspective of lower-GI microbiota-associated metabolic regulation, the more relevant finding is that the carrier itself showed microbiota-modulating effects during long-term administration. In db/db type 2 diabetic mice, oral administration of the CV@ALG carrier improved gut microbiota composition and markedly increased the abundance of *Akkermansia*, which was associated with enhanced insulin sensitivity. This result suggests that the microalgae-derived hydrogel carrier may provide metabolic benefits beyond insulin protection and pH-responsive release. In this case, the hydrogel matrix functions not only as a peptide delivery vehicle but also as a microbiota-modulating carrier that may improve insulin sensitivity through *Akkermansia*-associated pathways (Fig. 9).

By contrast, several pH-responsive or glucose-responsive hydrogels, including acid-resistant DNA hydrogels and phenylboronic acid- or ConA-containing glucose-responsive systems, mainly focus on improving oral insulin stability, intestinal absorption, and blood glucose control [[204], [205], [206]]. Although these systems are valuable for diabetes management, their relationship with lower-GI microbiota remodeling, microbial metabolites, epithelial barrier repair, or endotoxemia reduction remains relatively limited. Therefore, they are not discussed here as representative microbiota-associated metabolic hydrogel systems.

## 4 Emerging probiotic-delivering hydrogel strategies for microbiota modulation

The disease-centered hydrogel systems discussed above show that oral hydrogels can alleviate lower-GI-related disorders. These effects are often accompanied by changes in gut microbiota composition, microbial metabolites, epithelial barrier integrity, and immune responses. However, in many of these studies, microbiota alterations are mainly evaluated as treatment-associated outcomes rather than being incorporated as the primary design target. To advance from disease-centered therapy toward microbiota-guided design, this section focuses on probiotic-oriented hydrogel systems. In these systems, microbial viability, gastrointestinal protection, intestinal retention, mucosal colonization, and controlled release of living bacteria are considered central design elements. These systems highlight the potential of oral hydrogels to function not only as drug delivery carriers, but also as active platforms for delivering beneficial microbes and reshaping host–microbiota interactions.

### 4.1 Probiotic-delivering hydrogel systems for IBD therapy

Probiotic-based therapy has attracted increasing attention in IBD treatment because beneficial microorganisms can regulate gut microbial balance, reinforce epithelial barrier integrity, and modulate mucosal immune responses. However, the therapeutic efficacy of orally administered probiotics is often limited by gastric acid and bile salt damage, rapid intestinal clearance, poor colonization, insufficient lesion-site accumulation, and reduced viability in the inflammatory intestinal microenvironment [207,208]. In addition, IBD lesions are characterized by excessive ROS accumulation, epithelial barrier disruption, mucus damage, immune dysregulation, and microbial dysbiosis, which further impair probiotic survival and function [209,210]. Therefore, probiotic-protective hydrogel systems have been developed to improve bacterial viability during gastrointestinal transit, prolong intestinal retention, enhance mucosal adhesion or inflammatory-site targeting, and integrate probiotic delivery with barrier repair, oxidative stress regulation, and microbiota restoration.

**Fig. 10:**
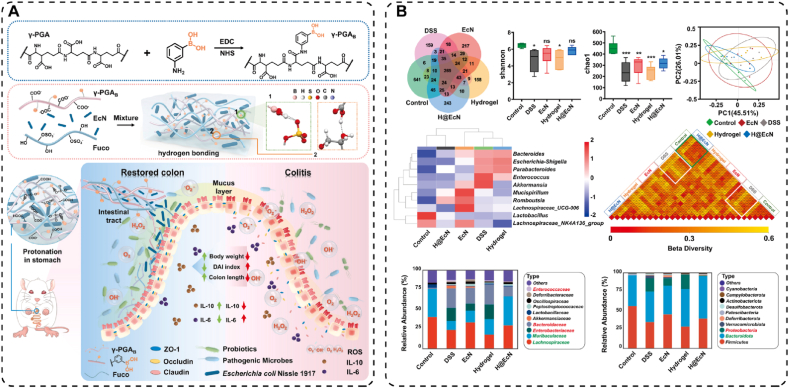
Fuco-PGAB hydrogel for enhanced probiotic delivery and microbiota restoration in colitis. (A) Schematic illustration of Fuco-PGAB hydrogel construction and its therapeutic mechanism, showing γ-PGAB–fucoidan co-assembly, probiotic encapsulation, protection during gastrointestinal transit, prolonged intestinal retention, and restoration of epithelial barrier function in DSS-induced colitis. (B) Gut microbiota analysis showing that H@EcN restored microbial diversity and community structure, reduced dysbiosis-associated taxa such as Enterobacteriaceae, Enterococcaceae, Bacteroidaceae, Proteobacteria, and *Escherichia-Shigella*, and enriched beneficial taxa including Lachnospiraceae, Muribaculaceae, and Bacteroidota. Reproduced with permission [213]. Copyright 2026, Elsevier.

For probiotic delivery in IBD, one major challenge is that most hydrogel carriers mainly provide physical protection but contribute limited intrinsic therapeutic activity. In addition, static hydrogel networks may be insufficient to resist the continuous mechanical stress caused by intestinal peristalsis, which can compromise probiotic retention and survival [211,212]. To address these limitations, Liu et al. [213] developed a bioengineered fucoidan-based hydrogel, Fuco-PGAB, by crosslinking fucoidan with γ-polyglutamic acid functionalized with 3-aminophenylboronic acid. This design enhanced hydrogen-bonding density and endowed the hydrogel with pronounced shear-thinning behavior and ultrafast self-healing properties, allowing the material to dissipate peristaltic stress and protect encapsulated probiotics during gastrointestinal transit. The hydrogel efficiently encapsulated both *Lactiplantibacillus plantarum 90* and *Escherichia coli Nissle 1917*, with encapsulation efficiencies of 98.32 ± 0.06% and 98.60 ± 0.14%, respectively. In simulated gastric fluid, H@EcN maintained 7.52 log CFU/mL after 0.5 h and 6.37 log CFU/mL after 1 h, whereas free EcN was completely inactivated at 1 h. In bile salt solution, H@EcN retained 5.63 log CFU/mL after 4 h, while free EcN became undetectable. *In vivo*, H@EcN further prolonged intestinal retention, with viable counts in the colon and cecum exceeding those of free EcN by 2.80 and 4.12 log CFU/mL at 96 h, respectively. In a DSS-induced colitis model, H@EcN alleviated weight loss, slowed DAI progression, preserved colon length, reduced IL-6 from 2.94 to 1.45 pg/mg, increased IL-10 from 0.39 to 1.99 pg/mg, and restored tight junction proteins, including claudin, occludin, and ZO-1. Microbiota analysis further showed that H@EcN restored microbial diversity, reduced harmful taxa such as *Enterobacteriaceae*, *Enterococcaceae*, *Bacteroidaceae*, *Proteobacteria*, and *Escherichia-Shigella*, and enriched beneficial taxa such as *Lachnospiraceae*, *Muribaculaceae*, and *Bacteroidota*. This study demonstrates that a bioactive hydrogel matrix can simultaneously improve probiotic survival, prolong intestinal retention, alleviate oxidative stress, reinforce the epithelial barrier, and reshape gut microbiota in IBD therapy (Fig. 10).

In addition to insufficient protection, another important limitation of probiotic therapy for IBD is the difficulty in delivering viable bacteria precisely to inflamed intestinal sites while simultaneously improving the oxidative inflammatory microenvironment. Excessive ROS accumulation in IBD damages epithelial cells, disrupts barrier integrity, and further reduces probiotic survival [214,215]. To overcome these challenges, Zhou et al. [216] developed dual-targeting Mn@CeO_2_ nanozyme-modified probiotic hydrogel microspheres, termed MnCe@LR/AMs. In this system, Mn-doped CeO_2_ nanozymes were first attached to the surface of *Limosilactobacillus reuteri* to enhance ROS-scavenging capacity, and the modified probiotics were then encapsulated into sodium alginate hydrogel microspheres using an electrostatic spraying method. The alginate microspheres protected probiotics from gastric acidity and provided dual targeting to inflamed intestinal sites through electrostatic attraction and mannose receptor binding. *In vitro*, MnCe@LR/AMs markedly enhanced probiotic resistance to gastrointestinal challenges: after simulated gastric fluid exposure, the survival rate of MnCe@LR/AMs was 9.43-fold higher than that of native *L. reuteri*, and after simulated intestinal fluid or H_2_O_2_ exposure, the survival rates were 19.56- and 10.88-fold higher, respectively. *In vivo* imaging and qPCR further confirmed enhanced adhesion and retention of *L. reuteri* in inflamed colonic tissue. In DSS-induced colitis mice, MnCe@LR/AMs improved body weight recovery, reduced DAI scores, restored colon length, alleviated histological injury, and increased goblet cells. The system also restored intestinal barrier proteins ZO-1 and occludin, reduced pro-inflammatory cytokines TNF-α and IL-1β, and increased anti-inflammatory cytokines IL-4 and IL-10. Importantly, 16S rDNA sequencing showed that MnCe@LR/AMs restored α-diversity and β-diversity, increased beneficial bacteria including *Bacteroidia*, *Clostridia*, *Bifidobacteriales*, *Ruminococcaceae*, *Bifidobacteriaceae*, *Limosilactobacillus*, *Lachnospiraceae_NK4A136_*group, and *Akkermansia*, while reducing potentially harmful taxa such as *Enterobacterales* and *Escherichia-Shigella*. Combined metabolomic and transcriptomic analyses further indicated that MnCe@LR/AMs enhanced amino acid metabolism, including histidine, arginine, and tryptophan pathways, and regulated macrophage polarization. This study highlights a probiotic hydrogel design strategy that integrates gastric protection, inflammation targeting, ROS scavenging, barrier repair, immune modulation, and microbiota remodeling [216].

IBD-associated complications, such as intestinal fibrosis and *Clostridioides difficile*-complicated colitis, are more difficult to treat because they involve not only mucosal inflammation but also extracellular matrix deposition, myofibroblast activation, microbial dysbiosis, and opportunistic pathogen overgrowth [217,218]. Although LL37 peptide and probiotics both show therapeutic potential, their oral application is limited by enzymatic degradation and rapid clearance [219]. Inspired by bacteriocin transport by bacteria, Yu et al. [220] developed an orally administered peptide–probiotic delivery platform, BTB-Alg, in which LL37-decorated probiotics were encapsulated within a protective sodium alginate shell. In this “all-in-one” architecture, probiotics served not only as therapeutic microorganisms but also as carriers for LL37, enabling spatiotemporal coordination between probiotic colonization and peptide release. The alginate layer protected LL37 from pepsin degradation, preserved probiotic viability, and enabled pH-responsive release in the intestinal environment. *In vivo* imaging showed that BTB-Alg prolonged intestinal residence of both probiotics and LL37, with Lac and LL37 signals derived from Lac/LL@Alg remaining higher than those of native Lac or free LL37 at 2, 4, 6, and 8 h after administration. At 8 h, the alginate layer showed substantial degradation, while Lac and LL37 retained 80.05% and 37.34% of their initial signals, respectively, indicating staged alginate separation and sustained biotherapeutic retention. In DSS-induced acute colitis, BTB-Alg reduced body weight loss and DAI scores, restored colon length, decreased TNF-α and MPO/MDA-related oxidative damage, increased IL-10, preserved goblet cells, and restored gut microbial homeostasis by increasing beneficial taxa such as *Bacillota*, *Muribaculaceae*, and *Ligilactobacillus*. In DSS plus *C. difficile*-challenged colitis, BTB-Alg downregulated colonic IL-6, IL-17, and IL-1β mRNA by 75.99%, 71.89%, and 23.79%, respectively, compared with the saline-treated group, enhanced ZO-1 expression, suppressed NF-κB signaling, and promoted macrophage polarization from the M1 phenotype toward the M2 phenotype. In chronic colitis-associated intestinal fibrosis, BTB-Alg reduced inflammatory progression, extracellular matrix deposition, Col I and α-SMA expression, and fibrosis-related transcripts, while metagenomic and proteomic analyses suggested that the therapeutic effect involved microbiota reprogramming and LL37-induced AMPK/mTOR-mediated autophagy. This study provides a representative example of a probiotic-based hydrogel system that goes beyond simple bacterial protection by integrating probiotics, therapeutic peptides, intestinal retention, microbiota reconstitution, immune regulation, and antifibrotic mechanisms.

Together, these probiotic-protective hydrogel systems demonstrate that oral hydrogels can be designed around the specific barriers and pathological features of IBD, including gastric acid and bile salt stress, rapid intestinal clearance, oxidative inflammatory microenvironments, impaired mucosal adhesion, epithelial barrier disruption, immune imbalance, and microbial dysbiosis. Their design logic mainly involves biocompatible encapsulation, shear-thinning and self-healing protection, inflammation-targeted adhesion, ROS scavenging, peptide–probiotic co-delivery, prolonged intestinal retention, and restoration of microbiota–barrier–immune homeostasis. Compared with disease-centered systems in which microbiota changes are mainly observed after treatment, these probiotic-delivering hydrogel systems place microbial viability, colonization, and ecological restoration closer to the initial design goal, thereby representing an important transition toward microbiota-oriented hydrogel therapy for IBD.

### 4.2 Probiotic-delivering hydrogel systems for CRC therapy

CRC is closely associated with gut microbiota dysbiosis, impaired intestinal barrier function, immunosuppressive tumor microenvironment, and altered microbial metabolite production. In addition to conventional chemotherapy and targeted therapy, probiotic-based interventions have attracted increasing attention because probiotics can regulate intestinal flora, produce antitumor metabolites, enhance epithelial barrier integrity, and modulate local immune responses [221,222]. However, orally administered probiotics often suffer from poor resistance to gastric acid, bile salts, digestive enzymes, antibiotics, and rapid intestinal clearance, which restricts their intestinal retention, tumor-site accumulation, and therapeutic efficacy. Therefore, probiotic-delivering hydrogel systems have been developed to improve bacterial survival in the gastrointestinal tract, prolong intestinal residence, enhance microbiota-mediated antitumor effects, and cooperate with chemotherapy or immunotherapy.

One major challenge in CRC immunotherapy is that the immunosuppressive tumor microenvironment can reduce therapeutic responsiveness, especially for strategies targeting TGF-β signaling [177]. Although Galunisertib, a TGF-β receptor I inhibitor, can suppress TGF-β/SMAD signaling, its antitumor efficacy is often limited by tumor heterogeneity and insufficient immune activation. To address this limitation, Niu et al. [132] developed an oral probiotic microgel system, EcN@(CS-SA)_2_, by coating *Escherichia coli* Nissle 1917 (EcN) with chitosan and sodium alginate through layer-by-layer assembly and Ca^2+^ crosslinking. In this design, sodium alginate provided a biocompatible gel matrix for probiotic encapsulation, while chitosan improved microgel stability, adhesion, and protection against the harsh gastrointestinal environment. EcN@(CS-SA)_2_ maintained high bacterial viability after coating, with viable bacteria accounting for 99.6%, and showed improved resistance to simulated gastric fluid, bile salts, and antibiotics compared with free EcN. *In vivo*, the microgel prolonged EcN residence in the small intestine, cecum, and colon, and slowed fecal bacterial clearance, indicating improved intestinal persistence.

In an orthotopic CRC mouse model, EcN@(CS-SA)_2_ reshaped gut microbiota composition by increasing beneficial bacteria with antitumor relevance, including *Lactobacillus*, *Akkermansia*, *Bifidobacterium*, *Muribaculaceae*, and *Akkermansia*, while reducing tumor-promoting microbial imbalance. Antibiotic depletion experiments further showed that the antitumor effect of EcN@(CS-SA)_2_ depended at least partly on intestinal flora, because antibiotic pretreatment weakened its tumor-suppressive efficacy. Mechanistically, EcN@(CS-SA)_2_ induced tumor cell apoptosis and immunogenic cell death, as reflected by increased extracellular ATP release, CRT exposure, and HMGB1 translocation. When combined with Galunisertib, EcN@(CS-SA)_2_ further reduced TGF-β levels, activated NLRP3 and IL-1β signaling, promoted dendritic cell maturation, and enhanced CD8^+^ T-cell infiltration into tumor tissues. *In vivo*, the combination of EcN@(CS-SA)_2_ and Galunisertib achieved the strongest tumor suppression, with DC maturation increasing to 66.2% and IFN-γ^+^ CD8^+^ T-cell infiltration rising markedly compared with Galunisertib or EcN alone. This study suggests that probiotic microgels can improve CRC immunotherapy by linking gastrointestinal probiotic protection, microbiota remodeling, immunogenic cell death, dendritic cell activation, and T-cell-mediated antitumor immunity.

Another important limitation of probiotic therapy for CRC is that probiotics alone may exhibit insufficient colonization and antitumor potency, while conventional chemotherapy such as 5-fluorouracil (5-FU) is limited by drug resistance, gastrointestinal toxicity, and disruption of gut microbiota. To enhance probiotic survival and simultaneously exploit prebiotic fermentation, Wang et al. [223] developed synbiotic hydrogel capsules, Lr@GI, by encapsulating *Lactobacillus reuteri* (Lr) within a gelatin–inulin hydrogel “shield”. In this system, inulin served as a prebiotic component and fermentable substrate, while gelatin improved hydrogel formation and structural stability through hydrogen-bonding interactions. The GI hydrogel shield enhanced the resistance of Lr to gastric acid and other gastrointestinal stresses without impairing bacterial growth or viability. Compared with free Lr, Lr@GI showed higher survival in simulated gastric fluid, bile salts, simulated intestinal fluid, and antibiotic solution. *In vivo*, Lr@GI increased viable bacterial counts in the stomach, small intestine, cecum, and colon, and promoted bacterial accumulation at orthotopic colon tumor sites, with the highest tumor-site colony count observed at 48 h after oral administration.

**Fig. 11:**
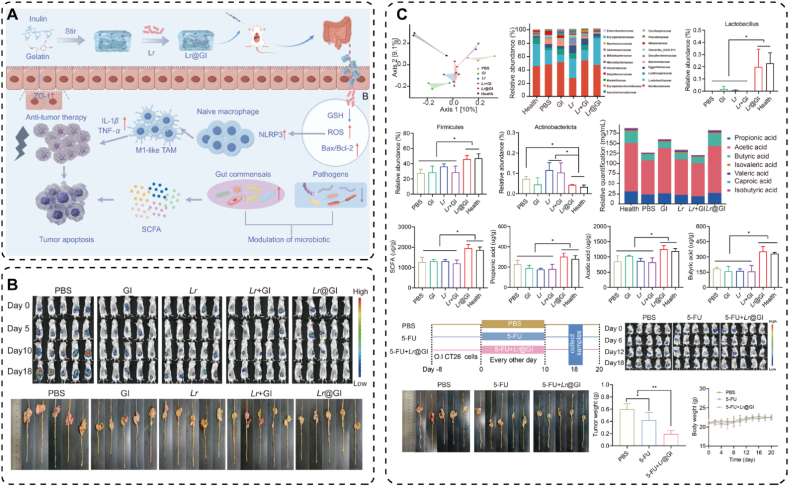
Synbiotic Lr@GI hydrogel capsules for colon cancer therapy and chemotherapy sensitization. (A) Schematic illustration of Lr@GI preparation and therapeutic mechanism, showing *Lactobacillus reuteri* encapsulated within a gelatin–inulin hydrogel “shield” for improved gastrointestinal protection, intestinal retention, microbiota modulation, SCFA production, GSH depletion, ROS generation, NLRP3 activation, and M1 macrophage polarization. (B) In vivo antitumor evaluation in orthotopic colon cancer mice, showing that Lr@GI reduced tumor growth, decreased tumor weight and Ki67 expression, and promoted tumor cell apoptosis compared with free Lr, GI hydrogel, or their simple mixture. (C) Gut microbiota, SCFA, and combination therapy analyses showing that Lr@GI reshaped intestinal microbiota, increased fecal SCFAs including acetate, propionate, and butyrate, and enhanced the antitumor efficacy of 5-FU without obvious body weight loss. Reproduced with permission [223]. Copyright 2025, Elsevier.

In orthotopic colon cancer mice, Lr@GI showed stronger antitumor efficacy than GI hydrogel, free Lr, or their simple mixture. Tumor weight was markedly reduced, Ki67 staining decreased, and TUNEL staining revealed extensive tumor cell apoptosis in the Lr@GI group. Mechanistically, Lr@GI depleted glutathione in tumor tissues, increased ROS production, activated the NLRP3 inflammasome, and promoted tumor-associated macrophage polarization toward the M1 phenotype. The M1/M2 macrophage ratio increased from approximately 0.15 in the Lr group and 2.85 in the Lr + GI group to 9.15 in the Lr@GI group, indicating strong immune remodeling toward an antitumor phenotype. Lr@GI also increased iNOS expression, reduced arginase expression, and elevated pro-inflammatory antitumor cytokines, including IL-1β and TNF-α. In addition to immune remodeling, Lr@GI enhanced intestinal barrier function by increasing ZO-1 expression. Microbiota analysis further showed that Lr@GI enriched Lactobacillaceae, Rikenellaceae, *Lactobacillus*, *Firmicutes*, *Muribaculaceae*, and *Prevotellaceae*, while reducing *Actinobacteria*. These microbial shifts were accompanied by increased fecal SCFAs, especially acetate, propionate, and butyrate, which were further shown to inhibit CT26 cell proliferation. Importantly, Lr@GI also potentiated the antitumor effect of 5-FU, leading to lower tumor fluorescence intensity and tumor weight than 5-FU alone, without obvious body weight loss. This study demonstrates that synbiotic hydrogel capsules can integrate probiotic protection, prebiotic fermentation, SCFA production, barrier repair, macrophage polarization, and chemotherapy sensitization for CRC therapy (Fig. 11).

Together, these probiotic-delivering hydrogel systems demonstrate that CRC-oriented microbial therapy can be strengthened by rational hydrogel design. EcN@(CS-SA)_2_ mainly improves CRC immunotherapy by protecting EcN during gastrointestinal transit, prolonging intestinal retention, remodeling gut microbiota, inducing immunogenic cell death, and enhancing Galunisertib-mediated antitumor immunity. Lr@GI further shows that a synbiotic hydrogel capsule can combine probiotic delivery with a prebiotic matrix, thereby improving bacterial survival, increasing SCFA-associated microbial metabolism, restoring epithelial barrier integrity, polarizing macrophages toward the M1 phenotype, and enhancing 5-FU chemotherapy. Their design logic mainly involves probiotic protection, intestinal retention, microbiota remodeling, microbial metabolite production, immune microenvironment activation, and combination with existing CRC therapies. Compared with conventional CRC hydrogel systems that mainly focus on chemotherapeutic drug delivery, these probiotic-delivering hydrogels place living bacteria and microbiota-mediated antitumor immunity closer to the therapeutic design center.

### 4.3 Probiotic-delivering hydrogel systems for gut–brain axis and other lower-GI-related disorders

Beyond IBD and CRC, gut microbiota dysbiosis is also closely associated with gut–brain axis dysfunction and functional lower-GI disorders, such as colitis-associated cognitive impairment, anxiety- or depression-like behaviors, and diarrhea-predominant IBS [224,225]. These conditions are often characterized by impaired epithelial barrier integrity, intestinal inflammation, altered microbial metabolites, visceral hypersensitivity, and abnormal gut–brain communication. Conventional treatments usually focus on symptomatic relief, while probiotic-based therapies may provide a more ecological approach by restoring microbial balance, improving mucosal barrier function, and modulating immune–neural signaling. However, orally administered probiotics or probiotic-like bioactive microorganisms still face limited gastrointestinal stability, poor intestinal retention, and insufficient mucosal colonization. Therefore, hydrogel systems that protect probiotics, enhance intestinal adhesion, and support site-specific colonization may provide new opportunities for treating gut–brain axis-related and functional lower-GI disorders.

**Fig. 12:**
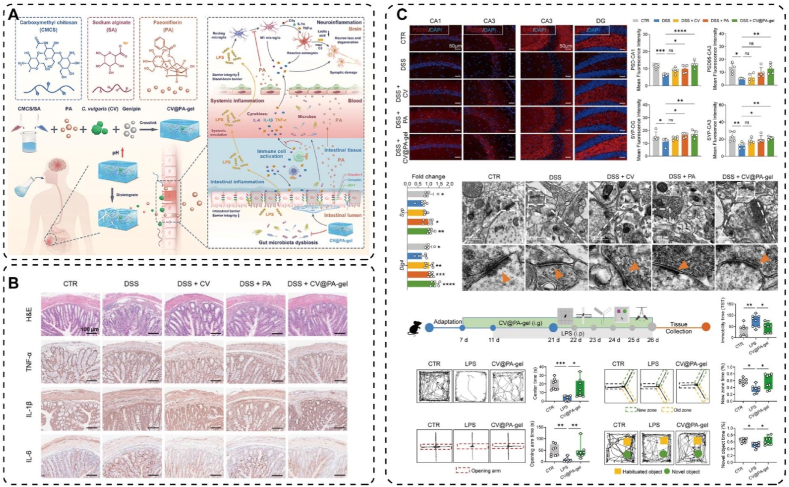
CV@PA-gel hydrogel microalgal platform for microbiota–gut–brain axis protection in chronic colitis. (A) Schematic illustration of CV@PA-gel preparation and therapeutic mechanism, showing paeoniflorin and *Chlorella vulgaris* co-encapsulated in a pH-responsive CMCS/SA hydrogel for colon-targeted release, intestinal barrier repair, microbiota regulation, and suppression of LPS-mediated systemic and hippocampal neuroinflammation. (B) Representative H&E and immunohistochemical staining of colonic tissues showing that CV@PA-gel alleviated DSS-induced mucosal injury and reduced inflammatory cytokine expression, including TNF-α, IL-1β, and IL-6. (C) Hippocampal immunofluorescence, ultrastructural synapse images, and behavioral tests showing that CV@PA-gel restored synaptic-related markers, protected neuronal ultrastructure, and improved anxiety-, depression-like, and cognitive behaviors associated with chronic colitis. Reproduced with permission [228]. Copyright 2025, Wiley-VCH.

For IBD-associated gut–brain axis dysfunction, one important pathological link is that intestinal barrier disruption can promote endotoxin leakage and systemic inflammation, thereby contributing to hippocampal neuroinflammation and behavioral abnormalities [226,227]. Paeoniflorin (PA) has anti-inflammatory and neuroprotective activity, but its poor oral bioavailability and rapid clearance limit its therapeutic effect. To address this limitation, Lu et al. [228] developed a colon-targeted pH-responsive hydrogel microalgal platform, CV@PA-gel, by co-encapsulating *Chlorella vulgaris* and PA within a genipin-crosslinked carboxymethyl chitosan/sodium alginate matrix. This system showed limited PA release under simulated gastric conditions but sustained release under intestinal pH, with PA release reaching 74.38% at pH 7.4 compared with 35.81% at pH 1.8 over 72 h. In chronic DSS-induced colitis mice, CV@PA-gel restored epithelial barrier integrity, reduced endotoxemia and complement C3 activation, inhibited microglia-mediated neurotoxic A1 astrocyte polarization, and improved anxiety-, depression-like, and cognitive behaviors. Microbiota and metabolomic analyses further showed enrichment of beneficial genera such as *Bifidobacterium* and *Lachnoclostridium*, together with regulation of fecal metabolites including azelaic acid, 3-indoleacetic acid, and tryptophan. This study suggests that microalgae-integrated hydrogel systems can connect colon-targeted delivery with microbiota regulation, barrier repair, systemic inflammation reduction, and neuroimmune protection through the microbiota–gut–brain axis (Fig. 12).

Similarly, insufficient inflamed-site colonization limits the therapeutic persistence of probiotics in colitis-associated cognitive disorders [229]. To improve probiotic viability and inflammation-targeted adhesion, Chen et al. [230] developed an aptamer-assisted hydrogel-encapsulated probiotic system, EcN-Apt@HG. In this system, an IL-6 aptamer was conjugated onto the surface of *Escherichia coli* Nissle 1917 to recognize IL-6-enriched inflamed mucosa, and the modified probiotics were further encapsulated in a dynamic Schiff-base hydrogel composed of aldehyde-functionalized chondroitin sulfate and amine-terminated PAMAM. The hydrogel protected EcN against gastric acid, bile salts, intestinal fluid, and oxidative stress, while aptamer modification enhanced adhesion to inflamed colonic sites. Compared with naked EcN, EcN-Apt@HG increased viable bacterial counts by approximately 6.9-fold in the intestine, 123.5-fold in the cecum, and 197.2-fold in the colon. In DSS-induced colitis mice, EcN-Apt@HG restored mucosal barrier proteins, modulated macrophage polarization, improved microbial diversity, enriched beneficial bacteria such as *Lactobacillus*, *Akkermansia*, and *norank_f_Muribaculaceae*, and reduced harmful taxa such as *Escherichia-Shigella*. More importantly for this section, EcN-Apt@HG relieved depression-like behavior, cognitive impairment, hippocampal neuroinflammation, and neuronal damage, indicating that inflammation-targeted probiotic hydrogels may improve gut–brain axis dysfunction by coupling local mucosal repair with microbiota–metabolite regulation.

Functional lower-GI disorders provide another important application scenario for probiotic-delivering hydrogel systems. IBS-D is characterized by diarrhea, abdominal pain, visceral hypersensitivity, mucosal immune activation, increased intestinal permeability, and gut microbiota dysbiosis. Although probiotics have shown potential for restoring intestinal microbial balance in IBS-D, their clinical efficacy is often restricted by poor survival during gastrointestinal transit and weak colonization in the mucus layer. To address this colonization barrier, Wu et al. [231] developed a “Janus” structured nanoclay microgel for targeted delivery of *Pediococcus pentosaceus Li05*. The microgel was fabricated using microfluidics and contained two functional hemispheres: a polydopamine-containing adhesive side and a nanoclay/probiotic side. Polydopamine promoted adhesion to intestinal mucins and prolonged intestinal residence, while nanoclay promoted Li05 aggregation and growth through charge interactions. The optimized 100 μm and 150 μm microgels showed high probiotic encapsulation efficiency of 99.93 ± 0.002% and 99.90 ± 0.004%, respectively, and sustained release of active Li05 in simulated intestinal fluid. The microgels also maintained Li05 viability under simulated gastric and intestinal conditions, suggesting improved oral probiotic bioavailability.

In an IBS-D rat model, the nanoclay microgel significantly alleviated diarrhea-related symptoms, visceral hypersensitivity, mucosal inflammation, and intestinal villus injury. Compared with the IBS-D group, nanoclay microgel treatment improved body weight loss, reduced abdominal withdrawal reflex scores, and decreased fecal water content, loose stool rate, diarrhea index, and diarrhea rate. Histological and ultrastructural analyses further showed reduced inflammatory infiltration and improved intestinal villus morphology. Mechanistically, the nanoclay microgel suppressed mucosal inflammation by downregulating inflammatory cytokines, including IL-1β, IL-2, IL-7, IL-18, IFN-γ, and RANTES, and inhibited the NLRP3 inflammasome pathway, as shown by reduced NLRP3, ASC, caspase-1, and GSDMD expression. It also modulated MUC2 and ZO-1 expression, suggesting partial restoration of mucosal barrier conditions. Microbiota analysis showed that nanoclay microgel treatment improved IBS-D-associated dysbiosis, increased beneficial taxa such as *Lachnospiraceae*, *Lactobacillaceae*, *Lachnospiraceae_NK4A136_*group, and *Lactobacillus*, and reduced potentially harmful taxa related to inflammatory activation. This study demonstrates that mucosa-adhesive and probiotic growth-supportive microgels can extend probiotic hydrogel therapy from inflammatory diseases to functional bowel disorders, where effective mucosal colonization and microbiota restoration are central therapeutic requirements.

Together, these probiotic-based hydrogel systems indicate that gut–brain axis-related and other lower-GI disorders require design strategies beyond simple probiotic encapsulation. For colitis-associated neurobehavioral dysfunction, hydrogel systems mainly improve intestinal retention, inflammation-targeted colonization, barrier repair, endotoxin leakage, microbiota–metabolite balance, and neuroimmune signaling. For IBS-D, the Janus nanoclay microgel further highlights the importance of mucus adhesion, probiotic growth support, sustained release, and functional colonization in treating diarrhea, visceral hypersensitivity, mucosal inflammation, and microbial dysbiosis. Therefore, this category of hydrogel systems expands probiotic delivery from IBD and CRC therapy toward broader lower-GI-related disorders by integrating gastrointestinal protection, mucosal residence, microbial ecosystem remodeling, immune regulation, and gut–brain axis modulation.

### 4.4 Colonization-enhancing hydrogel design for probiotic delivery

Although probiotic-delivering hydrogels can improve probiotic survival during gastrointestinal transit, stable intestinal colonization remains a central challenge for long-term probiotic therapy. Conventional oral probiotics are usually administered as planktonic cells, which are vulnerable to gastric acid, bile salts, antibiotics, intestinal peristalsis, mucus turnover, and competition from resident microbiota. As a result, their beneficial effects are often limited to the administration period, and sustained probiotic colonization is difficult to achieve. From a biological perspective, probiotic colonization is not simply a matter of survival, but involves a sequential process of reversible attachment, irreversible adhesion, microcolony formation, and extracellular matrix production [232,233]. Therefore, hydrogel systems designed for probiotic therapy should not only passively protect probiotics, but also provide a three-dimensional microenvironment that supports probiotic self-organization, stress adaptation, mucosal adhesion, and colonization-related gene expression.

**Fig. 13:**
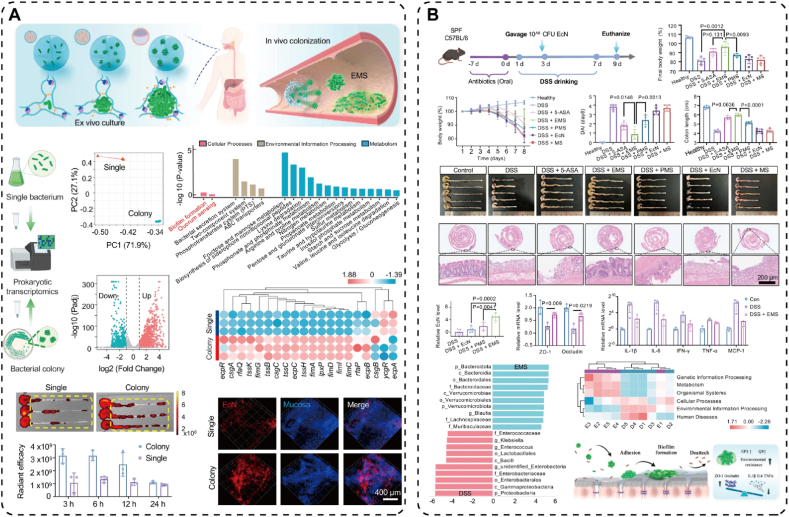
EMS hydrogel micro-cargo for enhanced probiotic colonization. (A) Schematic illustration of the Express Microcolony Service (EMS) design, showing *Escherichia coli* Nissle 1917 encapsulated in covalent–ionic crosslinked alginate hydrogel microspheres, which provide stress-relaxation, nutrient diffusion, and extracellular matrix support to promote multicellular microcolony self-organization. (B) Transcriptomic analysis comparing single bacteria and EMS microcolonies, showing upregulation of biofilm formation, quorum sensing, adhesion-related, exopolysaccharide-related, and stress-response genes in microcolonies, indicating enhanced colonization potential and environmental resistance. Reproduced with permission [234]. Copyright 2025, Springer Nature.

To address the poor adaptability and limited colonization of planktonic probiotics, Liu et al. [234] proposed a multicellular self-organized probiotic delivery strategy termed Express Microcolony Service (EMS). This strategy was based on the observation that multicellular probiotic microcolonies exhibit more favorable colonization-related gene patterns than single planktonic bacteria. In this system, *Escherichia coli Nissle 1917* (EcN) was encapsulated in covalent–ionic crosslinked alginate hydrogel microspheres, which combined methylacrylylated sodium alginate and classical calcium alginate networks. This dual-network hydrogel provided acid resistance, stress-relaxing mechanical behavior, tunable nutrient diffusion, and extracellular matrix support, thereby allowing dispersed probiotics to self-organize into compact microcolonies within the hydrogel micro-cargo. Transcriptomic analysis showed that multicellular colonies exhibited upregulated biofilm formation and quorum sensing pathways compared with single bacteria, together with enhanced expression of adhesion-related, extracellular polysaccharide-related, and stress-response genes. These results suggest that probiotic microcolonies possess greater intrinsic colonization potential than planktonic probiotics (Fig. 13).

A key design feature of EMS is the use of hydrogel viscoelasticity to regulate bacterial microcolony formation. The authors prepared alginate/AlgMA hydrogels with different AlgMA ratios, ranging from 0% to 100%, to tune matrix stiffness, stress relaxation, swelling, and degradation behavior. Hydrogels with lower AlgMA content exhibited faster stress relaxation, whereas higher AlgMA content improved network stability. Among these formulations, the 50% AlgMA hydrogel achieved a balance between gastrointestinal stability and colon-specific degradation: it remained stable in artificial gastric fluid and artificial intestinal fluid, but degraded more rapidly in artificial colon fluid. This formulation also supported better bacterial growth, with the highest OD600 value after 6 h of culture, and promoted higher expression of colonization-related genes, including *sdiA*, *csrA*, *csgD*, *csgA*, *adrA*, and *iraM*. The hydrogel micro-cargoes were prepared by a gas-shearing microfluidic method, producing uniform microspheres with an average diameter of 434 ± 12.75 μm. Within these microspheres, probiotics gradually formed self-organized microcolonies during culture, while the stress-relaxing matrix provided spatial constraints that prevented uncontrolled expansion and supported compact, metabolically active colony formation.

The EMS system further demonstrated that probiotic colonization can be enhanced by combining material protection with microcolony-level biological adaptation. During *in vitro* environmental challenge tests, the hydrogel matrix protected bacteria from gastric acid, while the multicellular microcolony structure further improved resistance to bile salts and antibiotics compared with both free single bacteria and uncultured bacteria encapsulated in hydrogel micro-cargoes. The enhanced resistance was associated with the dense microcolony structure and increased extracellular polysaccharide production. Compared with single bacteria, EMS-derived microcolonies showed higher adhesion to Caco-2 intestinal epithelial cells, approximately two-fold higher extracellular polysaccharide levels, and stronger biofilm-forming ability. *In vivo*, EMS markedly improved intestinal colonization after oral administration. Compared with primary hydrogel micro-cargoes containing dispersed bacteria, EMS achieved 89-fold higher EcN colonization in the cecum and 52-fold higher colonization in the colon, while only a limited increase was observed in the small intestine. These results indicate that hydrogel-assisted microcolony self-organization can shift probiotic delivery from transient survival toward stable lower-GI colonization.

The therapeutic relevance of this colonization-enhancing design was further evaluated in a DSS-induced acute colitis model. Because EcN is known to exert anti-inflammatory effects, protect the mucosal barrier, and regulate intestinal flora, the authors compared EMS with free EcN, primary micro-cargoes, blank micro-cargoes, and 5-ASA. A single oral dose of EMS maintained body weight, reduced disease activity, preserved colon length, and alleviated epithelial damage and immune cell infiltration. EMS also increased the expression of tight junction markers, including ZO-1 and occludin, and reduced inflammatory cytokines such as IFN-γ, IL-1β, IL-6, TNF-α, and MCP-1. Importantly, qPCR confirmed higher EcN abundance in colon tissues after EMS treatment compared with free EcN or primary micro-cargoes, supporting the relationship between enhanced colonization and therapeutic efficacy. Microbiota analysis showed that EMS reduced IBD-associated taxa such as *Enterococcaceae* and *Gammaproteobacteria*, while increasing beneficial bacteria including Bacteroides, *Lachnospiraceae*, *Akkermansia muciniphila*, and *Blautia*. Functional prediction further suggested a shift away from disease-associated and environmental stress-related pathways toward metabolism, genetic information processing, and organismal systems. Thus, EMS provides a representative example in which hydrogel mechanics, microcolony biology, colonization enhancement, microbiota remodeling, barrier repair, and colitis remission are integrated into one probiotic delivery strategy.

This study also provides several general principles for constructing colonization-enhancing probiotic hydrogels. First, the hydrogel should protect probiotics during upper GI transit while allowing release or degradation in the lower-GI tract. Second, the matrix should not be too rigid or too rapidly degradable; instead, stress-relaxing and partially dynamic networks can provide space for bacterial proliferation while maintaining microcolony integrity. Third, hydrogel design can regulate bacterial behavior, including quorum sensing, biofilm formation, extracellular polysaccharide production, stress resistance, and adhesion-related gene expression. Fourth, probiotic delivery units may be more effective when organized as multicellular microcolonies rather than isolated planktonic bacteria. Finally, colonization-enhancing hydrogels should be evaluated not only by short-term bacterial survival, but also by tissue-level colonization, microbiota remodeling, barrier restoration, immune regulation, and sustained disease improvement. Therefore, EMS represents an important transition from protective probiotic encapsulation toward hydrogel-guided probiotic self-organization and functional colonization.

## 5 Hydrogel–microbiota–host interactions: mechanistic insights for microbiota-guided design

As discussed in Section 2, oral hydrogel systems for lower-GI delivery are designed according to several key factors. These include gastrointestinal transit barriers, regional intestinal microenvironments, hydrogel composition, bioactive cargos, and nanoparticle-integrated functional elements. Based on these design principles, Sections 2.1, 2.1.1 further summarized disease-centered hydrogel therapies and emerging microbiota-oriented hydrogel strategies. These studies collectively show that oral hydrogels can improve local retention, protect therapeutic agents, enable controlled release, and alleviate lower-GI-related disorders. At the same time, many hydrogel treatments are accompanied by changes in gut microbiota composition, microbial metabolites, epithelial barrier integrity, and immune responses.

These observations indicate that the therapeutic performance of oral hydrogel systems is not determined only by material composition or drug release behavior. It is also shaped by their interactions with the microbiota–barrier–immune network in the lower-GI tract. The lower-GI tract is not simply a delivery destination; it is a complex ecological system composed of dense microbial communities, mucus layers, epithelial cells, immune components, and metabolite networks. Therefore, after summarizing disease-centered therapeutic outcomes and emerging microbiota-oriented strategies, this review further integrates the mechanistic links among hydrogel properties, delivery behavior, microbial remodeling, metabolite regulation, barrier repair, immune modulation, and disease improvement.

**Fig. 14:**
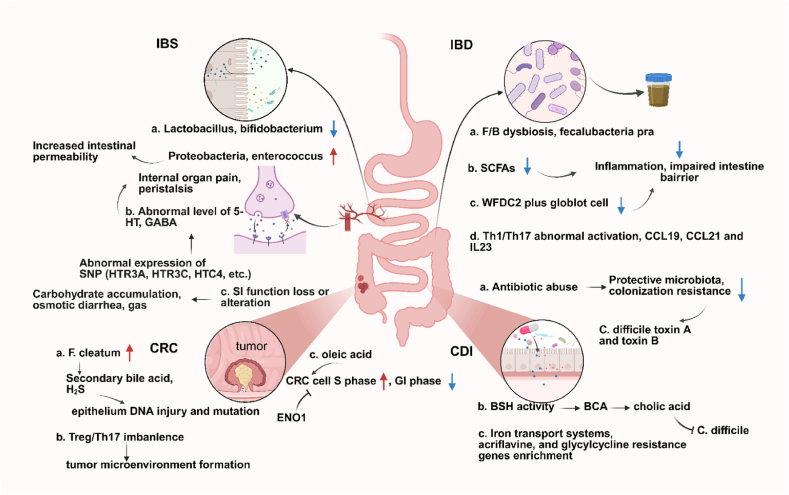
Microbial dysbiosis and immunological mechanisms relevant to microbiota-guided hydrogel design in lower-GI disorders. IBD: a. An imbalance in the ratio of Bacteroidetes to Firmicutes, particularly the depletion of *Faecalibacterium prausnitzii*, is closely associated with inflammation in IBD. b–c. A reduction in SCFAs and decreased expression of the protein WFDC2 contribute to intestinal inflammation and barrier dysfunction. d. Aberrant activation of Th1/Th17 cells, along with overexpression of chemokines CCL19, CCL21, and IL-33, further exacerbates disease progression. IBS: a. Decreased abundance of *Lactobacillus* and *Bifidobacterium*, alongside increased levels of *Proteobacteria* and *Enterococcus*, leads to enhanced intestinal permeability. b. Single-nucleotide polymorphisms in serotonin transporters and receptors alter the 5-HT signaling pathway, triggering visceral pain or abnormal motility. c. Loss or alteration of sucrase-isomaltase function causes undigested carbohydrates to accumulate in the colon, leading to osmotic diarrhea and gas production via microbial fermentation. CRC: a.CRC-associated microbial dysbiosis is accompanied by increased production of pro-tumorigenic metabolites, including secondary bile acids and hydrogen sulfide, while enriched bacteria such as *Fusobacterium nucleatum* further contribute to inflammation, epithelial damage, and tumor progression. b. Imbalance between regulatory T cells and Th17 cells contributes to the formation of a tumor-promoting microenvironment. c. Oleic acid enhances CRC cell survival by increasing the S-phase population while decreasing G1-phase cells, whereas inhibition of ENO1 suppresses proliferation. CDI: a. Antibiotic overuse reduces protective gut microbiota and impairs colonization resistance, increasing susceptibility to *Clostridioides difficile* infection. b. Bile salt hydrolase-producing bacteria convert taurine-conjugated bile acids into deoxycholic acid, which inhibits *C. difficile* growth. c. Enrichment of iron transport modules and antibiotic resistance genes is observed in patients with CDI. Created with BioRender.

In this section, we summarize microbiota dysbiosis and functional disturbances in representative lower-GI-related disorders, highlight key microbial metabolites as actionable targets for hydrogel design, and discuss barrier–immune–microbiota crosstalk relevant to hydrogel therapy. As summarized in Fig. 14, different lower-GI disorders involve distinct but interconnected microbiota-related mechanisms, including SCFA depletion and Th1/Th17 activation in IBD, microbial metabolite-driven tumorigenesis in CRC, neurotransmitter-related alterations in IBS and gut–brain axis disorders, and impaired colonization resistance in CDI.

### 5.1 Microbiota dysbiosis and functional disturbance in lower-GI disorders

Microbial dysbiosis is a common pathological feature of multiple lower-GI disorders. In IBD, patients often exhibit reduced microbial diversity and altered microbial composition, including imbalance between Bacteroidetes and Firmicutes and depletion of *Faecalibacterium prausnitzii* [235,236]. The reduction of microbial metabolites, especially SCFAs such as butyrate, compromises epithelial barrier integrity and promotes excessive immune activation [237,238]. In parallel, mucus depletion caused by reduced goblet cells and downregulation of WFDC2 weakens the mucosal barrier and facilitates microbial invasion [239,240]. Stromal-cell-derived chemokines, including CCL19, CCL21, and IL-33, further accelerate inflammatory progression [241]. In addition, decreased IPA-producing bacteria and reduced systemic indole-3-propionic acid (IPA) levels have been observed in IBD. IPA supplementation has been reported to alleviate experimental colitis, potentially through mechanisms involving HSP70-associated regulation of Th1/Th17 responses. [87,242]. These findings indicate that IBD-related dysbiosis involves not only compositional changes but also loss of microbial metabolites, mucus protection, epithelial barrier function, and immune-regulatory capacity. For hydrogel design, these mechanisms suggest potential targets such as SCFA-associated bacterial recovery, mucus-layer restoration, tight junction repair, and Th1/Th17-dominant inflammation regulation.

CRC is also closely associated with microbiota dysbiosis and microbial metabolic changes. Increased abundance of *Fusobacterium nucleatum* and *Escherichia coli* has been observed in CRC patients, and these bacteria contribute to tumorigenesis by producing pro-carcinogenic metabolites, including secondary bile acids and hydrogen sulfide, which can induce DNA damage and epithelial mutations [243,244]. Gut microbiota also modulates the balance between Tregs and Th17 cells, thereby shaping the tumor immune microenvironment [8,84]. Recent evidence indicates that microbiota-associated metabolites and host metabolic regulators are involved in CRC progression. Elevated oleic acid may promote tumor growth by supporting lipid metabolic reprogramming and proliferative signaling, whereas ENO1, a glycolytic enzyme frequently upregulated in CRC, contributes to aerobic glycolysis, tumor invasion, and immune evasion. These observations suggest that microbiota-driven metabolic alterations may influence CRC development through both lipid-metabolic and glycolytic pathways [245,246].

Several CRC-enriched bacteria, including *Peptostreptococcus stomatis*, *Fusobacterium nucleatum*, *Parvimonas micra*, *Peptostreptococcus anaerobius*, and *Bacteroides fragilis*, are increased in CRC-associated microbiomes, while beneficial taxa such as *Coprobacter fastidosus*, *Eubacterium ventriosum*, *Roseburia intestinalis*, and *Roseburia inulinivorans* are depleted [247,248]. These microbial signatures have been proposed as potential diagnostic markers for CRC [249]. From the perspective of hydrogel therapy, these findings support strategies that suppress tumor-associated bacteria, modulate H_2_S or bile-acid-related metabolism, improve local drug exposure, and remodel the tumor immune microenvironment.

Metabolic disorders, including obesity, diabetes, and NAFLD, are not lower-GI diseases in the anatomical sense, but their progression is closely linked to the gut microbiota–metabolite–barrier–immune axis. Transferring gut microbiota from obese mice or obese human donors into germ-free mice can induce increased body fat and elevated blood glucose in recipients, indicating a causal relationship between microbiota and metabolic phenotypes [250,251]. In metabolic disease, microbial imbalance can promote energy harvest, reduce SCFA-producing bacteria, impair epithelial barrier function, and facilitate LPS translocation into the bloodstream. This activates TLR4/NF-κB signaling in macrophages and adipocytes, resulting in chronic inflammation and insulin resistance [[252], [253], [254], [255]]. Disruption of bile acid metabolism also contributes to glucose and lipid metabolic disorders through FXR and TGR5 signaling [256,257]. Increased *Desulfovibrio* and H_2_S production can inhibit GLP-1 synthesis by enteroendocrine cells, aggravating glucose intolerance [258]. These mechanisms explain why microbiota-associated metabolic disorders can be discussed within lower-GI-focused hydrogel therapy when the intervention is mediated through intestinal microbial ecology, barrier integrity, endotoxemia reduction, or microbial metabolites.

IBS and gut–brain axis-related disorders are characterized by altered microbial composition, visceral hypersensitivity, intestinal barrier dysfunction, and abnormal neurotransmitter metabolism. IBS patients often exhibit decreased levels of beneficial bacteria, such as *Lactobacillus* and *Bifidobacterium*, along with increased *Proteobacteria* and *Enterococcus*, which may compromise barrier integrity and promote low-grade mucosal inflammation [[259], [260], [261]]. Microbiota-associated neuroactive molecules and neurotransmitter-related pathways, including5-HT and GABA signaling, may influence central nervous system activity through the gut–brain axis, thereby contributing to visceral hypersensitivity, altered motility, and behavioral symptoms [[262], [263], [264], [265]]. Sucrase-isomaltase deficiency further promotes undigested carbohydrate accumulation in the colon, resulting in osmotic diarrhea and gas production through microbial fermentation [266]. These mechanisms suggest that hydrogel systems for gut–brain axis-related disorders may act by improving intestinal retention of neuroactive compounds, regulating microbial metabolites, reducing barrier leakage, and suppressing gut-derived inflammatory signaling.

CDI represents a typical example of pathogen overgrowth caused by disruption of colonization resistance. Antibiotic use reduces microbial diversity and depletes protective taxa such as Bacteroidetes and Firmicutes, creating ecological niches for *C. difficile* colonization and toxin production [[267], [268], [269], [270], [271]]. Bile acid metabolism is closely involved in CDI resistance. Bile salt hydrolase-producing bacteria first deconjugate taurine-conjugated bile acids into primary bile acids, which can subsequently be transformed into secondary bile acids such as deoxycholic acid by 7α-dehydroxylating bacteria. [272]. Metagenomic analysis further revealed increased Enterobacteriaceae and functional enrichment of iron transport modules and antibiotic resistance genes in CDI patients [273]. These findings suggest that restoring colonization resistance, microbial diversity, and bile acid metabolism may be important targets for microbiota-guided hydrogel design, especially for hydrogel systems intended to deliver probiotics, phages, or defined microbial communities.

### 5.2 Microbial metabolites as actionable targets for hydrogel design

Microbial metabolites provide functional links between gut microbiota, epithelial barrier integrity, immune regulation, and disease progression. Among them, SCFAs, secondary bile acids, tryptophan-derived metabolites, and hydrogen sulfide represent important molecular targets for microbiota-guided hydrogel design.

SCFAs, including acetate, propionate, and butyrate, regulate intestinal homeostasis by providing energy to epithelial cells, reinforcing tight junctions, modulating luminal pH, and suppressing excessive inflammation [[274], [275], [276], [277]]. SCFAs also regulate immune cell function by activating G protein-coupled receptors and inhibiting histone deacetylases [278,279]. Butyrate promotes Treg differentiation and immune tolerance through mechanisms involving histone deacetylase inhibition and G protein-coupled receptor signaling. [280]. In IBD and metabolic disorders, reduced SCFA production is associated with epithelial barrier dysfunction, microbial invasion, and systemic inflammation [254,255]. Therefore, hydrogel systems designed to deliver fermentable polysaccharides, protect SCFA-producing bacteria, or support microbial fermentation may help restore SCFA-mediated barrier and immune regulation.

Secondary bile acids are another important class of microbiota-derived metabolites. Deoxycholic acid and lithocholic acid regulate intestinal and systemic immune responses by activating FXR and TGR5 [281,282]. These receptors are expressed in intestinal epithelial and immune cells, and their activation can inhibit NF-κB signaling and attenuate inflammatory responses. In CDI, microbial bile salt hydrolase activity contributes to the conversion of taurine-conjugated bile acids into secondary bile acids that suppress *C. difficile* growth [272]. In metabolic disorders, disruption of microbial bile acid transformation impairs FXR/TGR5 signaling and contributes to glucose and lipid metabolic dysregulation [256,257]. These findings suggest that hydrogel systems responsive to microbial enzymes or capable of delivering bile acid-modulating agents may be useful for restoring bile acid-mediated intestinal homeostasis.

Tryptophan-derived metabolites participate in both intestinal immune regulation and gut–brain communication. Indole and its derivatives regulate immune cell function by activating the aryl hydrocarbon receptor (AhR), promoting Treg differentiation and suppressing excessive Th17 responses [283,284]. In IBD, reduced IPA-producing bacteria and decreased systemic IPA levels are associated with intestinal inflammation, whereas IPA supplementation alleviates colitis by binding to HSP70 and inducing apoptosis of Th1 and Th17 cells [87,242]. In IBS and gut–brain axis-related disorders, tryptophan metabolism and serotonin-related signaling are closely associated with visceral sensitivity, motility, mood regulation, and neuroimmune communication [262,263]. Hydrogel systems that improve the intestinal delivery of tryptophan-modulating compounds or reshape microbial tryptophan metabolism may therefore influence both local intestinal inflammation and systemic neuroimmune responses.

Hydrogen sulfide represents a context-dependent microbial metabolite with particular relevance to CRC and metabolic dysfunction. In CRC, excessive bacteria-derived H_2_S has been associated with tumor-promoting effects, including epithelial DNA damage, altered mitochondrial metabolism, and enhanced proliferation in CRC models. [243,244]. In metabolic disorders, increased *Desulfovibrio*-derived H_2_S can inhibit GLP-1 production, thereby worsening glucose intolerance [258]. These findings indicate that hydrogel systems capable of reducing excessive H_2_S or modulating H_2_S-producing microbial communities may provide a strategy for reshaping disease-associated microbial metabolic environments.

### 5.3 Barrier–immune–microbiota crosstalk relevant to hydrogel therapy

The intestinal microenvironment is a complex ecosystem composed of epithelial cells, immune cells, microbial communities, and metabolites. These components interact continuously to maintain intestinal homeostasis and regulate local and systemic immune responses [285]. For oral hydrogel systems, this crosstalk provides both biological barriers and therapeutic opportunities.

The epithelial barrier is the first interface between the host and intestinal microbiota. Intestinal epithelial cells provide a physical and biochemical barrier, while goblet cells secrete mucus that limits direct contact between microbes and epithelial surfaces [286,287]. Paneth cells contribute to antimicrobial defense and can participate in tissue repair under pathological conditions [288]. Enteroendocrine cells regulate gastrointestinal activity and metabolic homeostasis through hormone secretion [289]. Intestinal stromal cells redistribute to damaged areas and support mucosal repair [290]. These cellular components are directly relevant to hydrogel therapy because bioadhesive and mucosa-protective hydrogels can prolong local retention, protect damaged epithelial surfaces, deliver repair-promoting factors, and support tight junction restoration.

Immune cells respond to microbial signals through pattern recognition receptors, including TLRs and NLRs, which recognize microbial-associated molecular patterns such as LPS, peptidoglycan, and flagellin [291,292]. Activation of these receptors induces NF-κB and MAPK signaling and promotes cytokine secretion, including IL-1β, IL-6, and TNF-α. The gut microbiota also regulates the balance between Tregs and Th17 cells, which is critical for maintaining immune tolerance and host defense [293,294]. Certain commensal microbes promote Th17 differentiation, whereas others, including members of *Bacteroides* and *Clostridium*, favor Treg induction [295,296]. Macrophages, ILC3, tissue-resident memory T cells, and other immune populations further participate in mucosal inflammation, epithelial repair, and host–microbiota communication [297,298]. Hydrogel systems that modulate cytokine release, macrophage polarization, or Treg/Th17 balance may therefore indirectly reshape microbial homeostasis by improving the immune conditions of the intestinal niche.

Gut microbiota-derived metabolites also connect intestinal immunity with systemic immune responses. SCFAs can enter the circulation and influence hematopoietic stem cell differentiation and immune cell development in the bone marrow [299,300]. By promoting tight junction expression and preserving the mucus layer, gut microbes reduce microbial translocation and systemic inflammation [301]. SCFAs also promote peripheral Treg expansion, reduce pro-inflammatory cytokine production, and enhance dendritic cell and macrophage function [302,303]. These mechanisms highlight the systemic impact of local hydrogel–microbiota–host interactions and help explain why intestinal hydrogel therapy may influence extraintestinal metabolic or neurological outcomes.

The major epithelial, immune, microbial, and metabolite-related components involved in intestinal homeostasis are summarized in Table 4. These components represent important biological interfaces for hydrogel therapy, because oral hydrogels may interact with mucus layers, epithelial cells, immune cells, microbial communities, and microbial metabolites during intestinal retention, degradation, cargo release, barrier repair, and microbiota modulation.

**Table 4:** The regulation of intestinal homeostasis.

	Classification	Effect on intestinal	References
Intestinal cells	Mesenchymal stem cell	Mesenchymal stem cells secrete CCL2 and CXCL12	[304]
	Paneth cells	Under pathological conditions, PCs can be dedifferentiated into stem cells to promote the repair of intestinal tissues	[305]
	Intestinal Mucosal Mast Cells	Mast Cells as Neuro-Immune Players in the Regulation of Intestinal Barrier Function	[306]
	Intestinal stromal cells	Actively redistributes to damaged areas and helps mucosal repair	[307]
	Intestinal epithelial cells	provide a physical and biochemical barrier	[3]
	enteroendocrine cells	Different hormones are produced to regulate gastrointestinal activity and nutritional homeostasis	[308,309]
	RPPFs	Paracrine control is exerted on tumor-initiating stem cells via the druggable PGE2/Ptger4/Yap signaling axis	[310]
Immune cells	Tissue-resident memory T	In IBD, the number of pro-inflammatory TRM cells increases, whereas the number of regulatory subgroups decreases	[311]
	B-cell	Impaired expression of the costimulatory molecule CD80, inducing tolerance	[312]
	Eosinophils	release lipid mediators leukotrienes, and smooth muscle contractions; Modulation of Th17 cells by production of IL-1 receptor antagonists	[313,314]
	Th2 cell	IL-10 secretion activates the Jak1/STAT3 signaling pathway and inhibits the secretion of inflammatory cytokines	[315]
	Microglia	Regulates inflammation, synaptic plasticity, and neural network formation	[316]
	ILC3	Modulates interactions with the microbiota through the production of IL-17 and IL-22	[317]
	Th17	IL-17A production plays a role in inflammatory diseases and tumor environments	[318]
	Macrophages	PGE2 40 factor is produced, stimulates epithelial progenitor cell proliferation in intestinal crypts, and regulates the epithelial barrier	[319]
Gut microbiota	*Bacteroides fragilis*	Promote the expression of pro-inflammatory cytokines and nitric oxide, and induce immune cell differentiation	[315]
	*Lactobacillus casei BL23*	Immunomodulation mediated by downregulating IL-22 cytokines	[320]
	*E. coli Nissle 1917*	Secretion of C18-3OH acts through activation of PPARγ	[321]
	*Bacteroides*	Influences the occurrence of epilepsy by regulating the maturation and activation of glial cells	[322]
	*Lactic acid bacteria*	produce a neurotransmitter called gamma-amino butyric acid (GABA), which helps to control feelings of fear and anxiety	[323]
	*Prausnitzii*	Producing butyrate maintains intestinal barrier function and has immunoregulatory and anti-inflammatory properties	[324]
	*Propionibacterium freudenreichii*	The release of propionate increases the expression of genes associated with Tc17 cells and CD8 CTL and promotes antitumor effects	[325]

Beyond cargo-mediated regulation, the hydrogel matrix itself may also contribute to hydrogel–microbiota–host interactions after intestinal degradation. This is particularly relevant for natural polysaccharide-based hydrogels, such as alginate, chitosan, pectin, inulin, cellulose derivatives, and other dietary fiber-like polymers. Many of these polysaccharides resist digestion in the upper GI tract and can reach the cecum or colon, where they may be degraded or fermented by microbial enzymes. The resulting oligosaccharides or fermentation products can serve as prebiotic-like substrates, supporting the growth or metabolic activity of beneficial bacteria and contributing to SCFA production. Therefore, natural polysaccharide hydrogels may exert a matrix–cargo dual regulatory function: the hydrogel network protects and releases loaded cargos, while the degraded matrix itself may help reshape the microbial community and metabolite profile. This matrix-mediated effect has been systematically discussed in previous reviews on natural polysaccharide-based colon-targeted hydrogel systems; therefore, we briefly highlight it here as an important component of hydrogel–microbiota–host interactions rather than providing an exhaustive discussion.

### 5.4 Hydrogel systems for emerging microbiota-based therapies

Emerging microbiota-based therapies, including phage therapy, fecal microbiota transplantation (FMT), defined microbial consortia, and microbiota-derived extracellular vesicles, provide new opportunities for reshaping the intestinal ecosystem. However, their therapeutic translation remains limited by poor gastrointestinal stability, insufficient intestinal retention, uncontrolled release, variable biological activity, and safety concerns related to undefined microbial components. In this context, hydrogels are not merely passive carriers, but adaptable platforms that can protect fragile biological cargos, localize treatment within the lower-GI tract, regulate release kinetics, and improve the controllability of microbiota-based interventions.

Phage therapy is particularly suitable for hydrogel-assisted delivery because phages can selectively eliminate pathogenic bacteria while preserving commensal microbes, but free phages are easily inactivated by gastric acid and often show low intestinal bioavailability. Yang et al. [326] developed double-responsive oral hydrogel microspheres composed of sodium alginate, HA, and Eudragit S100 for intestinal delivery of a Salmonella-targeting phage cocktail. The hydrogel microspheres achieved approximately 90% phage encapsulation efficiency, protected phages from simulated gastric conditions, prolonged intestinal retention, and enabled pH-responsive release in the intestine. In a Salmonella Typhimurium-induced colitis model, HMs-Phages reduced intestinal *Salmonella Typhimurium* (STm) burden by nearly 2000-fold and decreased TNF-α, IL-6, and IL-1β levels to approximately 60% of those in the infected group. Compared with ciprofloxacin, this strategy achieved comparable antibacterial efficacy while avoiding antibiotic-associated microbiota dysbiosis and diarrhea. This study demonstrates that oral hydrogel microspheres can support in situ gut microbiota editing by combining pathogen-specific eradication with commensal microbiota preservation.

Hydrogels may also support more complex microbiota-based interventions by protecting living components or FMT-derived functional cargos. For example, Tao et al. [327] developed a dual-layer living hydrogel, ProΦGel, in which phages were incorporated into an outer gelatin-PEG hydrogel and *Lactobacillus plantarum* was encapsulated in inner alginate microbeads. Although this system was designed for wound infection rather than intestinal delivery, its pathogen-responsive and sequential release design provides a useful concept for future gut-oriented living hydrogels that combine phages, probiotics, or engineered microbes. For FMT-related therapy, Zu et al. [327] showed that chitosan/alginate hydrogel encapsulation improved the richness, diversity, bioavailability, and intestinal colonization of orally transplanted microbiota compared with unprotected microbiota transplantation. The same study further proposed extracellular vesicles derived from nanomedicine-trained gut microbiota as a safer alternative to FMT. These Gm-EVs alleviated DSS-induced UC by reducing inflammatory cytokines, restoring ZO-1 and occludin-1 expression, reshaping gut microbiota composition, and regulating purine metabolism to reduce uric acid levels. Although this work does not yet represent a complete hydrogel-based Gm-EV delivery system, it supports the feasibility of integrating FMT-derived microbial components or EV-based therapeutics with protective hydrogel carriers.

Overall, the main value of hydrogels in emerging microbiota-based therapies lies in their ability to protect phages, probiotics, microbial consortia, or microbiota-derived vesicles during gastrointestinal transit; enhance lower-GI localization and mucosal retention; enable responsive or sequential release; and reduce systemic exposure or uncontrolled microbial dissemination. Therefore, hydrogel platforms may help transform phage therapy, FMT-derived interventions, and other microbiota-based strategies into more stable, localized, and controllable therapeutic systems for lower-GI disorders.

## 6 Outlook and conclusions

### 6.1 Outlook

With advancements in materials science, microbiome research, and pharmaceutical engineering, oral hydrogel systems have shown considerable potential for lower-GI drug delivery and microbiota-associated therapy. These systems can protect therapeutic agents from gastric acidity and enzymatic degradation, improve local retention, enable delayed or stimuli-responsive release, and deliver diverse bioactive agents, including conventional drugs, biologics, probiotics, prebiotics, and microbiota-modulating agents.

**Fig. 15:**
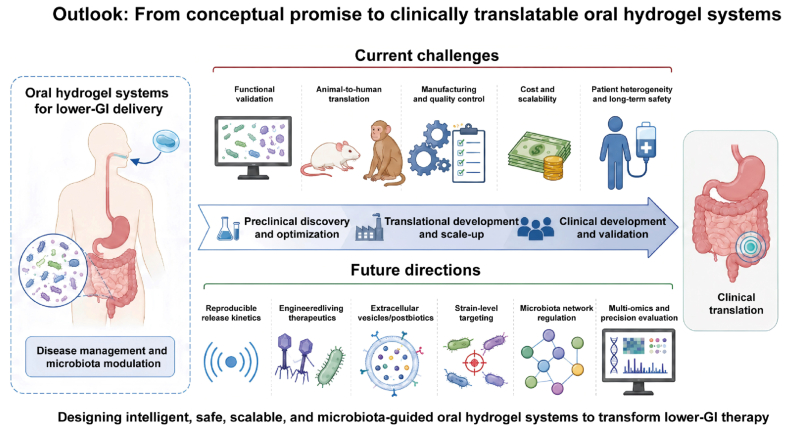
Future directions for clinically translatable oral hydrogel systems in lower-GI diseases therapy.

Currently, oral hydrogels can improve payload protection, reduce premature degradation, prolong intestinal residence, and enhance local therapeutic exposure. Nevertheless, several key challenges remain unresolved, including unstable release kinetics under dynamic GI conditions, insufficient regional and lesion-specific targeting, limited strain-level or function-level microbiota selectivity, weak validation of microbiota functional restoration, substantial patient heterogeneity, and unclear clinical translation pathways [328,329]. Therefore, future studies should establish clearer relationships among hydrogel design parameters, regional GI physiology, microbiota function, disease mechanisms, and translational evaluation criteria. Based on these bottlenecks, future development may focus on the following aspects (Fig. 15).

#### 6.1.1 From broad stimuli responsiveness to reproducible release kinetics

A major bottleneck of current oral hydrogel systems is that many “stimuli-responsive” designs have not yet been fully evaluated under physiologically relevant GI conditions. Hydrogels are often described as pH-, enzyme-, ROS-, or microbiota-responsive, but their response thresholds, release kinetics, and *in vivo* reproducibility are not always systematically characterized. In particular, some pH-responsive systems are designed based on the simplified assumption that pH increases progressively from the stomach to the intestine. However, the pH differences among the distal small intestine, cecum, and proximal colon can be relatively narrow and highly variable, which may limit the accuracy of pH-triggered lower-GI release. In addition, many release studies are still performed in static buffer systems, which only partially reproduce the complexity of gastric emptying, bile salts, digestive enzymes, mucus turnover, intestinal peristalsis, and fed/fasted-state variations.

Future hydrogel design may therefore benefit from moving from broad responsiveness toward more reproducible and quantitatively described release kinetics. One feasible route is to construct sequentially responsive systems in which different triggers play distinct roles during GI transit. For example, acid-resistant networks can reduce gastric leakage, delayed swelling or time-dependent erosion can support distal intestinal transit, microbial enzyme-sensitive linkages can promote cecal or colonic activation, and ROS-responsive bonds can further facilitate release at inflamed lesions. To better compare different systems, future studies could report parameters such as gastric leakage rate, swelling ratio, degradation half-life, release half-time, residual payload activity, and mucosal retention time. More physiologically relevant models, including sequential simulated GI fluids, mucus-containing diffusion systems, dynamic digestion models, intestinal organoids, gut-on-a-chip platforms, and ex vivo intestinal tissues, would also help evaluate whether hydrogel responsiveness is predictable and reproducible across different GI conditions.

#### 6.1.2 Matching bioactive agents selection with disease mechanisms and microbiota functions

Another important challenge is the need to better align encapsulated bioactive agents with disease pathology and microbiota-related functions. Many hydrogel systems incorporate anti-inflammatory drugs, antioxidants, growth factors, probiotics, prebiotics, nucleic acids, or nanoparticles. However, in some studies, the rationale for payload selection is mainly based on the general therapeutic potential of these agents, rather than on a clearly defined match with disease mechanisms or microbial functional deficits. This may lead to increasingly complex formulations whose mechanistic advantages are not always easy to distinguish.

Future studies may benefit from establishing a more explicit “payload–release site–microbiota function–therapeutic outcome” relationship. For IBD, payload selection could be considered not only in relation to suppression of pro-inflammatory cytokines, such as TNF-α, IL-6, or IL-1β, but also in relation to epithelial barrier repair, mucus restoration, immune-cell rebalancing, and recovery of beneficial microbial metabolites such as SCFAs. For CRC, hydrogel systems may need to evaluate not only tumor growth inhibition, but also tumor-associated microbiota, local immune activation, tumor microenvironment remodeling, and the safety of prolonged intestinal exposure. For metabolic disorders, microbiota-modulating hydrogels could further clarify whether microbial changes are linked to functional outputs such as bile acid metabolism, endotoxin reduction, glucose tolerance, insulin sensitivity, lipid metabolism, and SCFA production. In this way, future hydrogel systems may be designed according to disease-specific pathological mechanisms, rather than simply increasing the number of incorporated therapeutic components.

#### 6.1.3 From taxonomic shifts to functional microbiota restoration

A limitation in many current studies is that taxonomic changes are sometimes interpreted as evidence of microbiota restoration. Increased abundance of potentially beneficial genera or decreased abundance of potentially pathogenic taxa after hydrogel treatment can provide useful clues, but such compositional changes do not necessarily indicate functional recovery of the gut ecosystem. For example, an increase in *Lactobacillus* or *Akkermansia*, or a decrease in *Proteobacteria*, may be associated with improved intestinal health; however, these findings alone are not sufficient to demonstrate restoration of microbial metabolic capacity, epithelial barrier function, immune tolerance, or colonization resistance.

Future research may therefore need to move from taxonomic description toward functional validation. In addition to 16S rRNA sequencing, approaches such as shotgun metagenomics, metatranscriptomics, metabolomics, and microbial functional assays could help determine whether hydrogel treatment is associated with recovery of microbiota function [330]. Useful readouts may include SCFA production, bile acid transformation, tryptophan metabolism, mucin utilization, antimicrobial peptide induction, epithelial tight junction expression, mucus integrity, and immune tolerance markers. Moreover, microbiota findings from animal models should be interpreted with caution, because microbial composition, diet, immune background, mucus structure, and GI transit can differ substantially between animals and humans. Cross-model validation using human-derived microbiota models, intestinal organoids, ex vivo mucus systems, gnotobiotic animals, and patient-derived samples may provide stronger evidence for functional microbiota restoration. Such a balanced interpretation would help reduce the risk of overestimating the microbiota-regulating capacity of hydrogel systems.

#### 6.1.4 Improving spatial precision: regional targeting, mucus interaction, and strain-level selectivity

Spatial precision remains an important challenge for oral hydrogel delivery. Many current systems are designed to target broad intestinal regions, while more precise delivery to specific lower-GI segments, diseased lesions, microbial niches, or bacterial strains remains difficult. For example, some colon-targeted systems rely mainly on pH-triggered release, although the distal small intestine, cecum, and colon differ not only in pH, but also in bacterial density, microbial enzyme activity, mucus thickness, and residence time. Similarly, inflamed mucosa, ulcerated epithelium, and tumor tissues present diverse pathological cues, including ROS accumulation, matrix metalloproteinase activity, altered mucus structure, immune-cell infiltration, and abnormal vascular permeability. These complex and dynamic features may limit the reliability of single-trigger targeting strategies.

Future strategies could improve spatial precision at several levels. First, regional targeting may be refined by integrating pH gradients, transit time, bacterial enzyme activity, and mucus thickness to better distinguish distal small intestinal transit, cecal activation, and colonic release. Second, lesion-level targeting could make use of disease-specific cues, such as inflamed mucus, ulcerated epithelium, tumor acidity, elevated ROS, MMPs, and immune-cell recruitment, to enhance local retention and release at pathological sites. Third, strain-level or function-level microbiota targeting may be explored using bacterial adhesins, lectin–glycan interactions, phage-derived binding proteins, antimicrobial peptides, engineered probiotics, or CRISPR-associated modules. At the same time, these advanced approaches need careful evaluation for potential off-target ecological effects, unintended depletion of commensal bacteria, horizontal gene transfer, antibiotic resistance transfer, and long-term microbiota instability. Thus, spatial precision may progress gradually from regional release to lesion-specific retention and, ultimately, to function- or strain-level microbial regulation.

#### 6.1.5 Addressing patient heterogeneity and personalized hydrogel therapy

Patient heterogeneity is an important factor that may limit the clinical translation of oral hydrogel systems. Even patients with the same clinical diagnosis can differ substantially in baseline microbiota composition, intestinal pH, mucus thickness, gastrointestinal transit time, diet, antibiotic exposure, disease subtype, inflammation severity, medication history, age, and metabolic status. These differences can influence hydrogel swelling, degradation, microbial enzyme-triggered activation, mucosal adhesion, drug release, and overall therapeutic response. Therefore, a single universal hydrogel formulation may not be adequate for complex lower-GI or microbiota-associated disorders.

Future development could incorporate patient stratification into hydrogel design and evaluation. Stool metagenomics, metabolomics, and clinical phenotyping could help identify microbial functional deficiencies, such as reduced SCFA production, altered bile acid metabolism, increased abundance of endotoxin-producing bacteria, or impaired colonization resistance. These profiles may guide the selection of hydrogel materials, responsive linkages, active agents, prebiotics, probiotics, or postbiotics. Patient-derived microbiota culture systems, intestinal organoids, gut-on-a-chip platforms, and ex vivo mucus models may further help predict hydrogel behavior before clinical application. In the longer term, integrating microbiome data, disease phenotypes, and hydrogel response parameters could support predictive models linking a patient's microbiota profile with hydrogel performance and therapeutic outcome. Such patient-stratified strategies may enhance therapeutic precision and reduce unnecessary exposure to ineffective or poorly matched formulations.

#### 6.1.6 Translational evaluation: manufacturability, safety window, and regulatory classification

A further challenge is the gap between promising preclinical performance and practical clinical translation. Many oral hydrogel systems show favorable results in animal models, but their reproducibility, sterilization, storage stability, scalability, and regulatory pathway are not always fully addressed. Complex multi-component hydrogels, especially those containing nanoparticles, biologics, probiotics, engineered bacteria, stem cells, or gene-editing components, may face additional challenges related to manufacturing consistency, long-term safety, and regulatory classification. Therefore, translational considerations would ideally be incorporated at an early design stage rather than only after preclinical validation.

Future studies could adopt more practical and standardized evaluation dimensions. For material manufacturing, useful parameters may include batch-to-batch consistency, polymer molecular weight distribution, crosslinking degree, particle size distribution, mechanical stability, sterilization compatibility, storage stability, and GMP manufacturing compatibility. For delivery performance, studies could report drug-loading efficiency, encapsulation efficiency, gastric leakage rate, intestinal release kinetics, GI retention time, mucus adhesion or penetration capacity, and release kinetics stability under simulated fed and fasted states. For safety evaluation, long-term toxicity, local mucosal irritation, systemic exposure, immunogenicity, microbiota disturbance, chronic administration safety, and therapeutic safety window may need to be assessed. In particular, for synthetic or semi-synthetic hydrogel systems intended for repeated or long-term oral administration, the potential toxicity of residual monomers, initiators, organic solvents, or non-degradable crosslinking agents should be carefully considered, because these components may accumulate locally in the intestine or disturb the mucosal and microbial microenvironment over time. To reduce these risks, future designs may favor physically crosslinked networks, ionically crosslinked polysaccharide systems, enzyme-degradable linkers, or dynamic covalent bonds, such as Schiff base, boronate ester, disulfide, or hydrazone bonds, which may improve degradability, reversibility, and clearance while avoiding persistent non-degradable residues. For systems containing live bacteria, engineered probiotics, or gene-editing components, additional concerns include genetic stability, environmental shedding, horizontal gene transfer, antibiotic resistance markers, reversibility of colonization, and unintended ecological effects. From a regulatory perspective, multifunctional hydrogel systems may fall between drug, device, biologic, live biotherapeutic product, or combination-product categories. Early clarification of regulatory classification, together with GMP-compatible manufacturing, scalable encapsulation, validated quality control assays, and release specifications, would be important for advancing oral hydrogel systems toward clinical application.

### 6.2 Conclusion

Oral hydrogel systems have emerged as versatile platforms for lower-GI delivery because they can protect therapeutic cargos during upper-GI transit, prolong intestinal residence, and enable localized or stimuli-responsive release in diseased intestinal regions. In this review, we summarized the physiological and ecological barriers that affect oral hydrogel delivery, the major material and functional components of hydrogel systems, and their disease-centered applications in IBD, CRC, gut–brain axis-related disorders, and microbiota-associated metabolic disorders. Current evidence indicates that oral hydrogels can improve drug stability, enhance local retention, suppress inflammation, promote epithelial and mucus barrier repair, remodel tumor or inflammatory microenvironments, and support the delivery of probiotics, prebiotics, microbial metabolites, and other microbiota-modulating agents. These properties provide a material basis for expanding oral hydrogels from conventional drug delivery carriers toward platforms that interact with the lower-GI ecological niche.

Nevertheless, the microbiota-regulating role of oral hydrogel systems should be interpreted carefully. In many existing studies, gut microbiota changes are still evaluated mainly as treatment-associated outcomes rather than being predefined as primary design targets. Moreover, taxonomic shifts alone do not necessarily indicate functional restoration of the gut ecosystem. Therefore, future microbiota-guided hydrogel design may need to move beyond compositional analysis and incorporate functional readouts, such as microbial metabolite recovery, epithelial barrier restoration, immune rebalancing, colonization resistance, and host–microbiota metabolic interactions. In this context, hydrogels may contribute not only by delivering therapeutic agents, but also by shaping the spatial, biochemical, and ecological conditions under which microbiota–host interactions occur.

Overall, the transition from disease-based therapy to microbiota-guided design represents an important direction for the next generation of oral hydrogel systems. Future progress will depend on the development of reproducible release kinetics, region- and lesion-specific targeting, rational matching between bioactive agents and disease mechanisms, function-oriented microbiota modulation, patient-stratified evaluation, long-term biosafety assessment, and scalable manufacturing. By integrating materials science, microbiome functional analysis, pharmaceutical engineering, and translational evaluation, oral hydrogel systems may gradually evolve into more controllable and clinically relevant therapeutic platforms for lower-GI and microbiota-associated disorders.

## CRediT authorship contribution statement

**Haoming Wu:** Investigation, Validation, Visualization, Writing – original draft. **Jingjing Tian:** Validation, Writing – original draft. **Wuzheng Luo:** Validation, Writing – original draft. **Jiayu Liu:** Validation, Writing – original draft. **Yingying Chen:** Data curation, Visualization. **Kaiwen Yang:** Data curation, Visualization. **Jixin Zhou:** Data curation, Visualization. **Jingwen Li:** Data curation, Visualization. **Shuhao Yang:** Data curation, Visualization. **Yao Zhang:** Data curation, Visualization. **Kainan Li:** Conceptualization, Supervision, Writing – review & editing. **Gaohui Zhu:** Conceptualization, Funding acquisition, Supervision, Writing – review & editing. **Shuai Tan:** Conceptualization, Funding acquisition, Supervision, Writing – review & editing. **Xulin Hu:** Conceptualization, Funding acquisition, Supervision, Writing – review & editing.

## Declaration of competing interest

The authors declare no conflict of interest.

## Data Availability

No data was used for the research described in the article.
