# Supplementary material for: Oral hydrogel systems in lower gastrointestinal disorders: From disease-based therapy to microbiota-guided design
Source: Mater Today Bio. 2026 Jun 16;39:103357. doi: 10.1016/j.mtbio.2026.103357 (PMC13320260; doi:10.1016/j.mtbio.2026.103357)

**The permissions of Figure1**


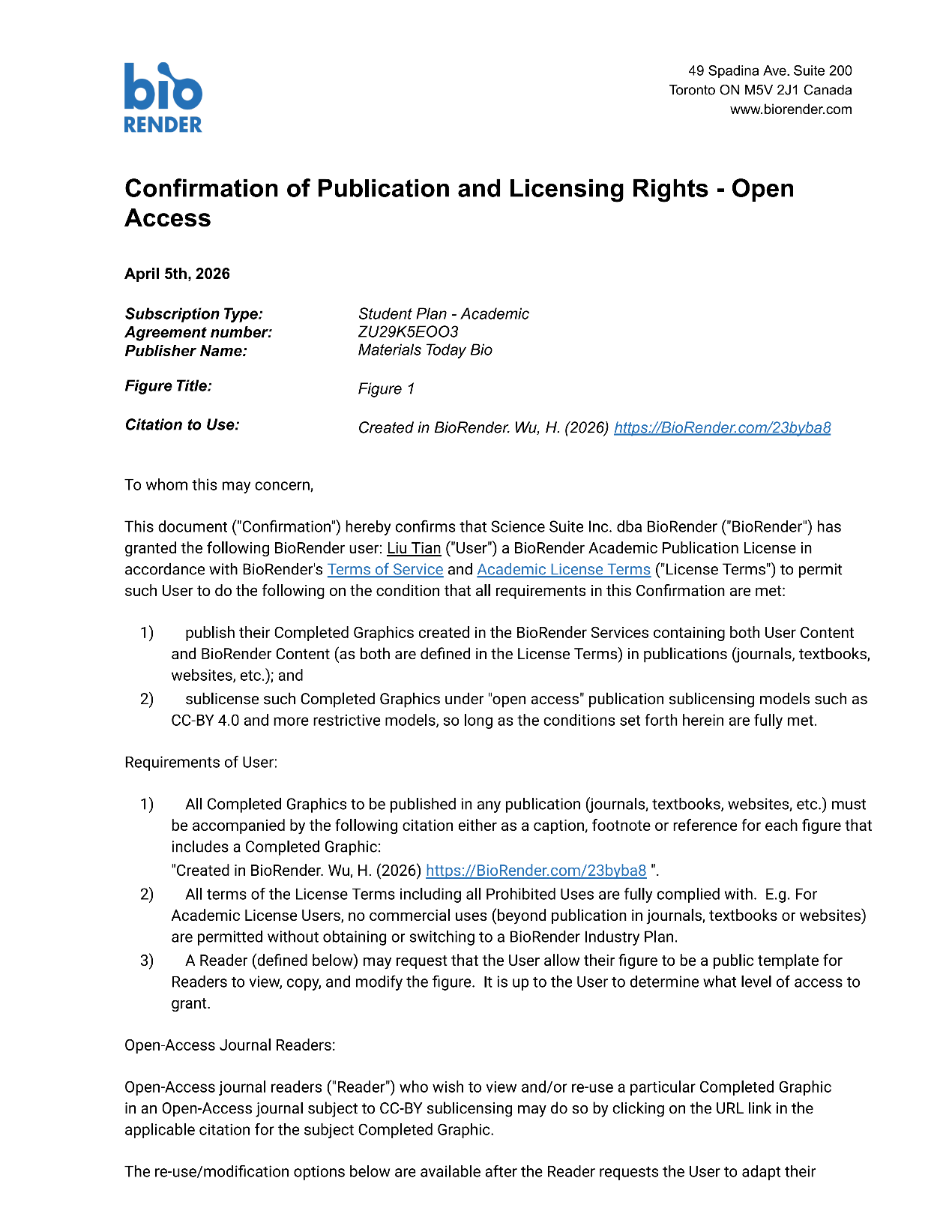

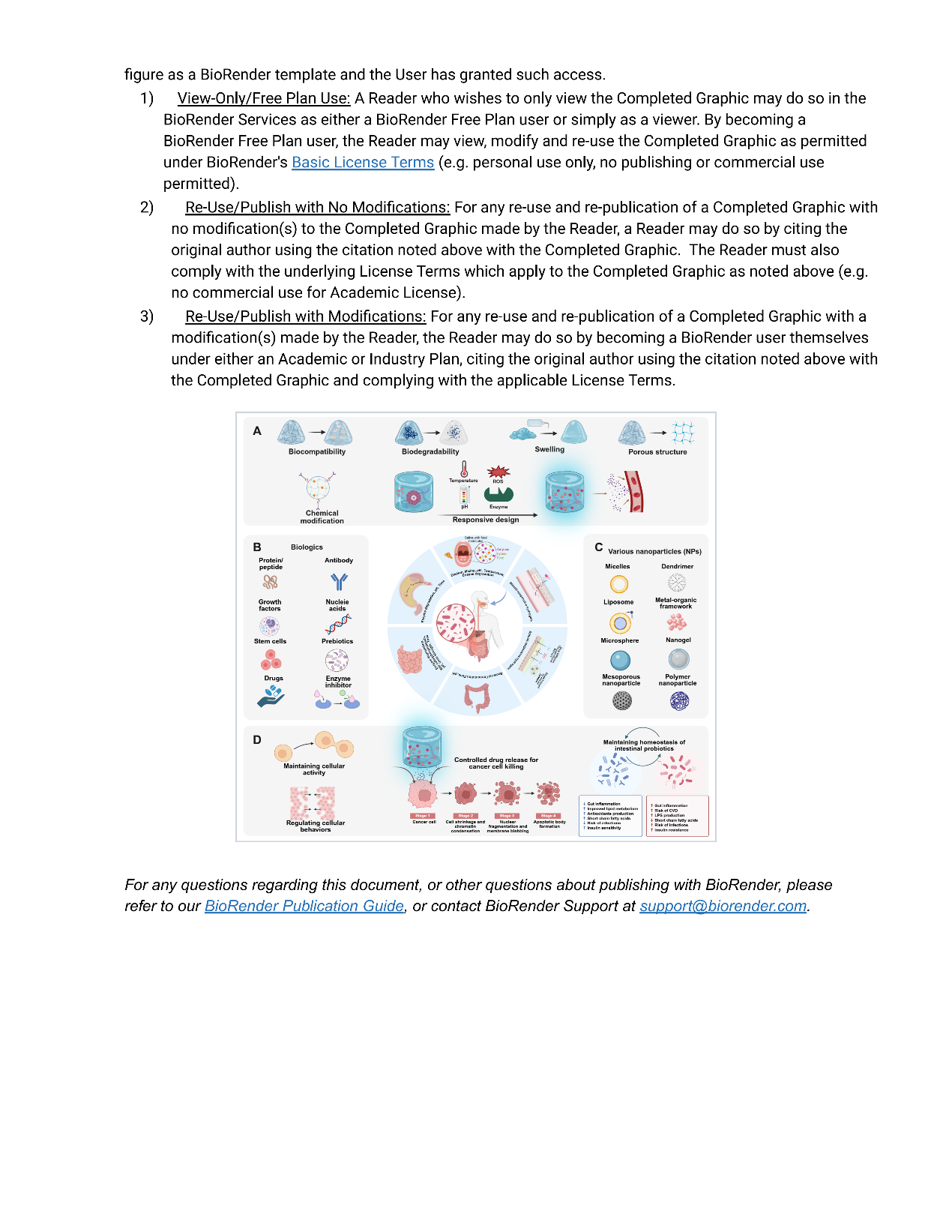


**The permissions of Figure2**


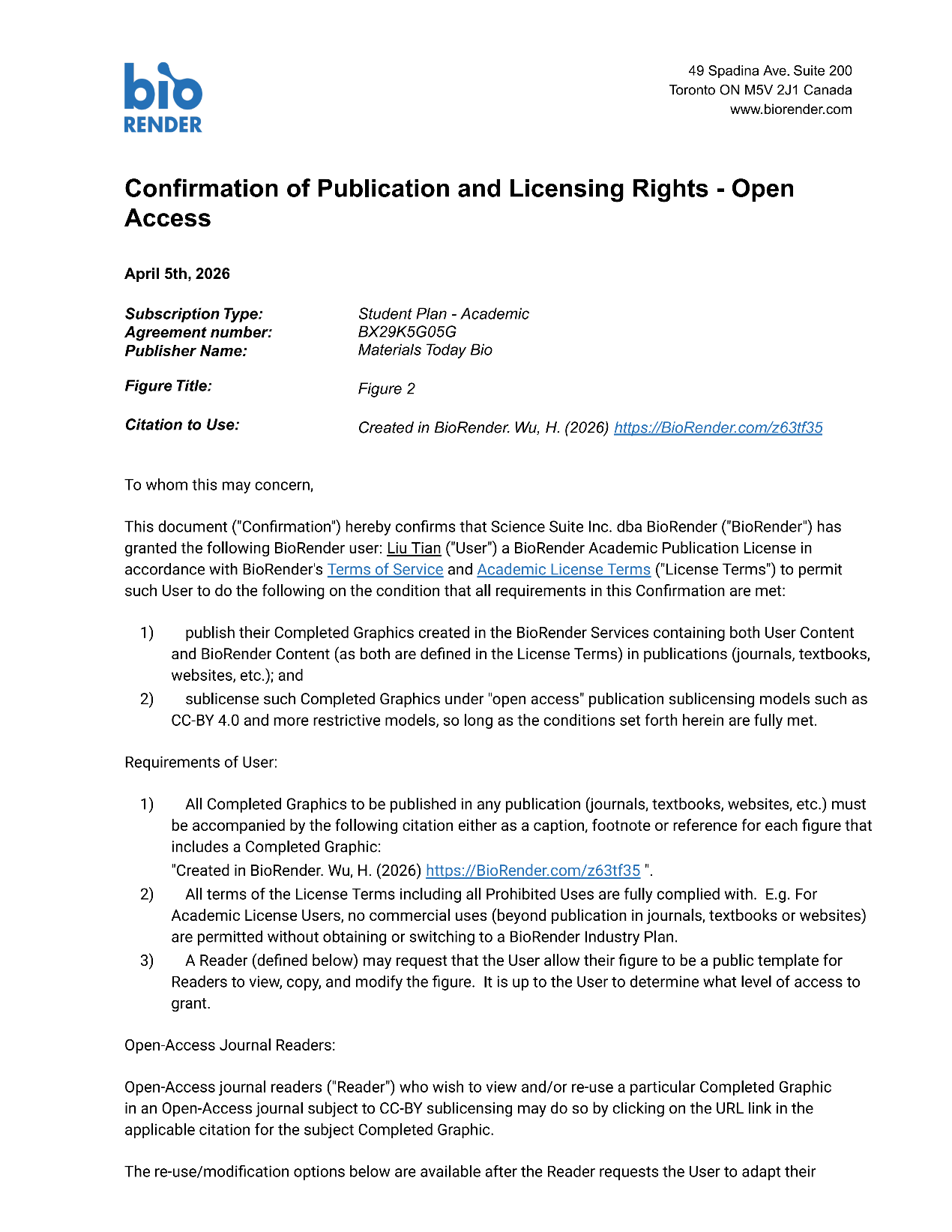


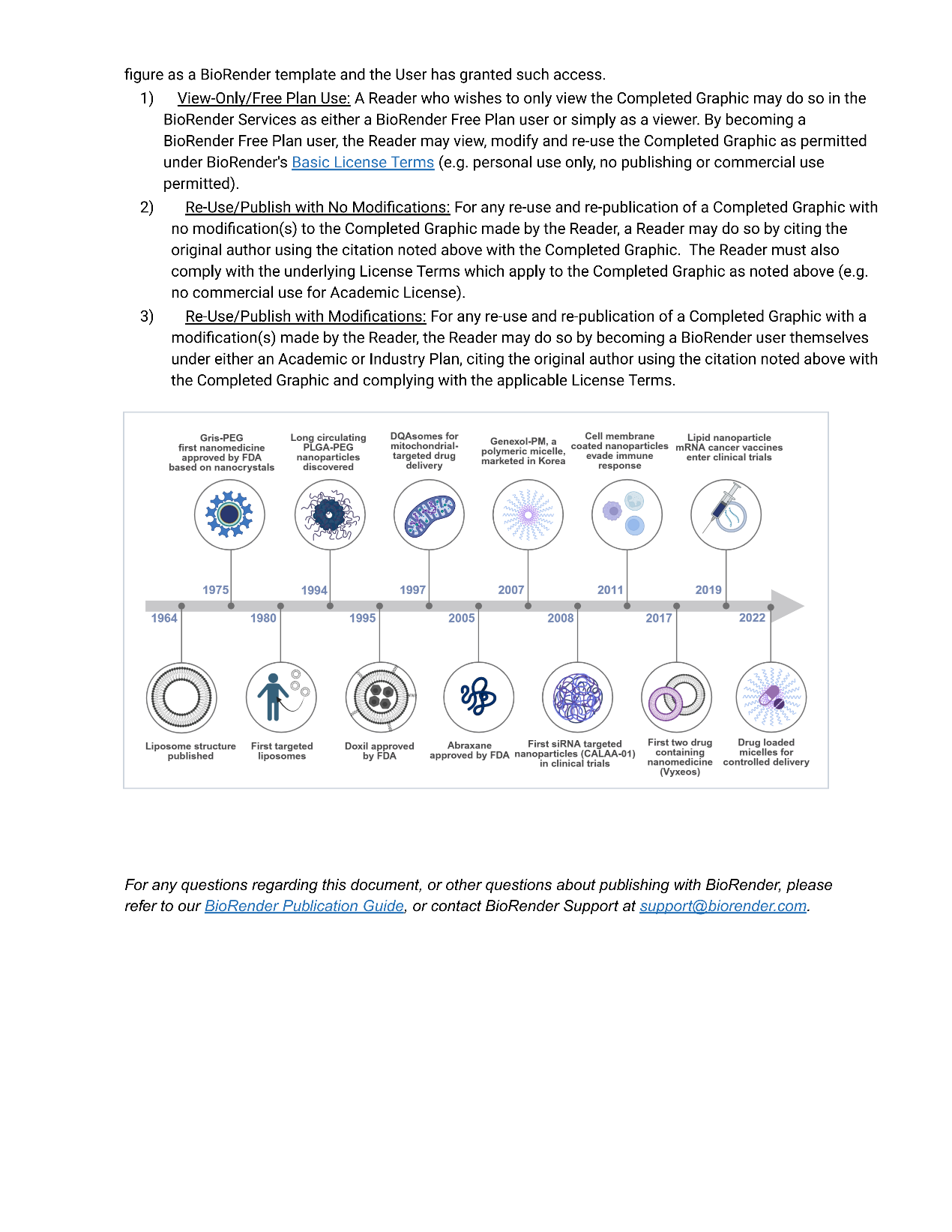


**The permissions of Figure 3**


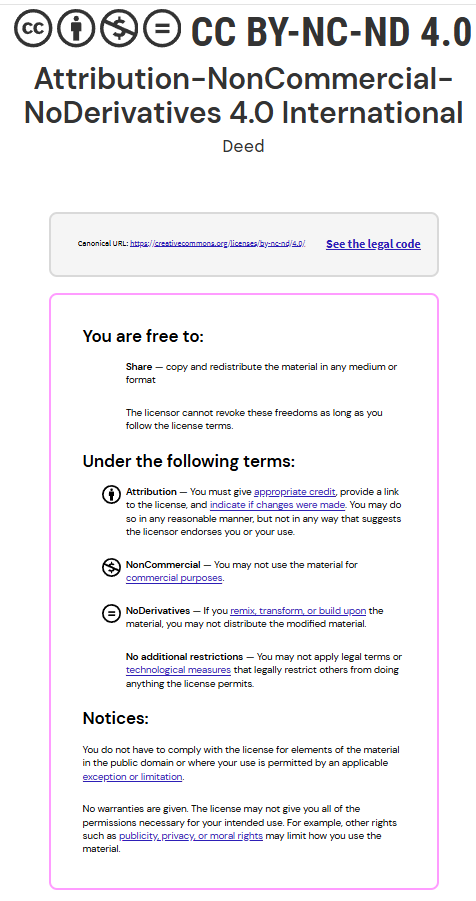


**The permissions of Figure 4**

**
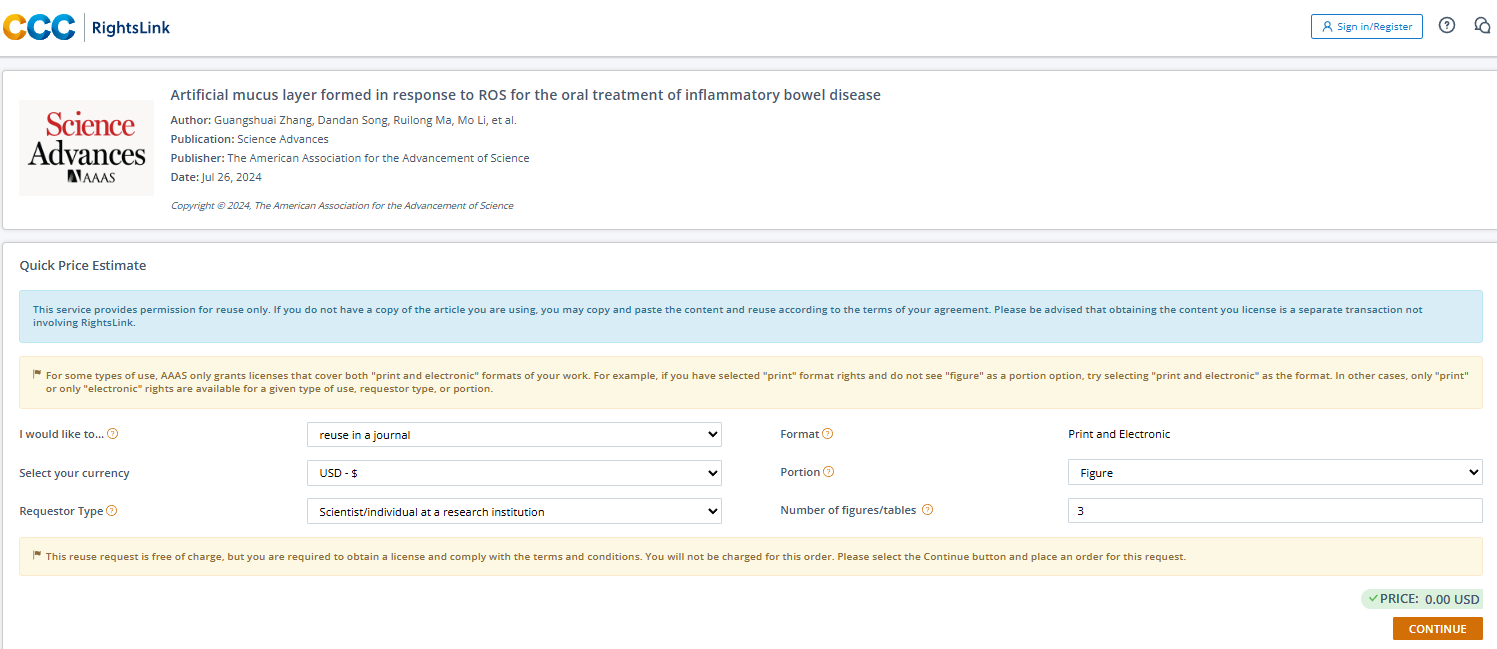
**

**The permissions of Figure 5**

**
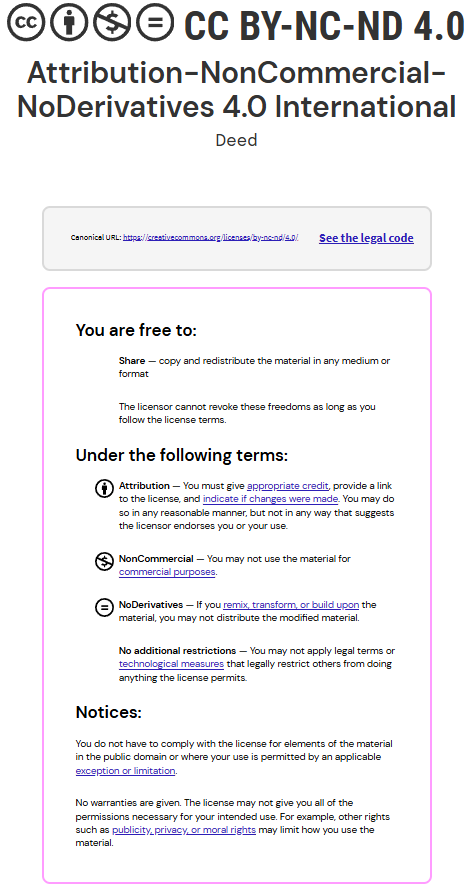
**

**The permissions of Figure 6**

**
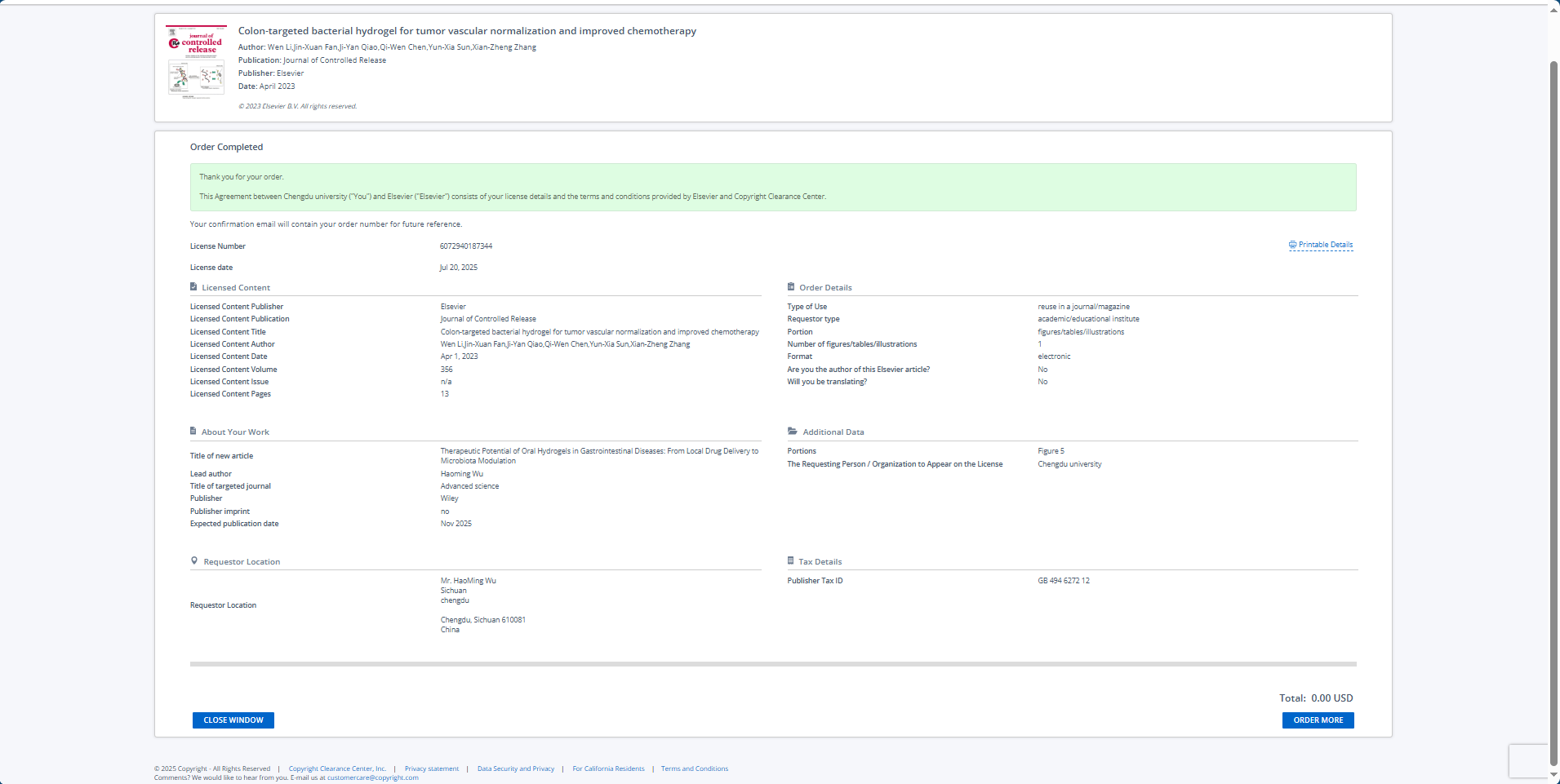
**

**The permissions of Figure 7**

**
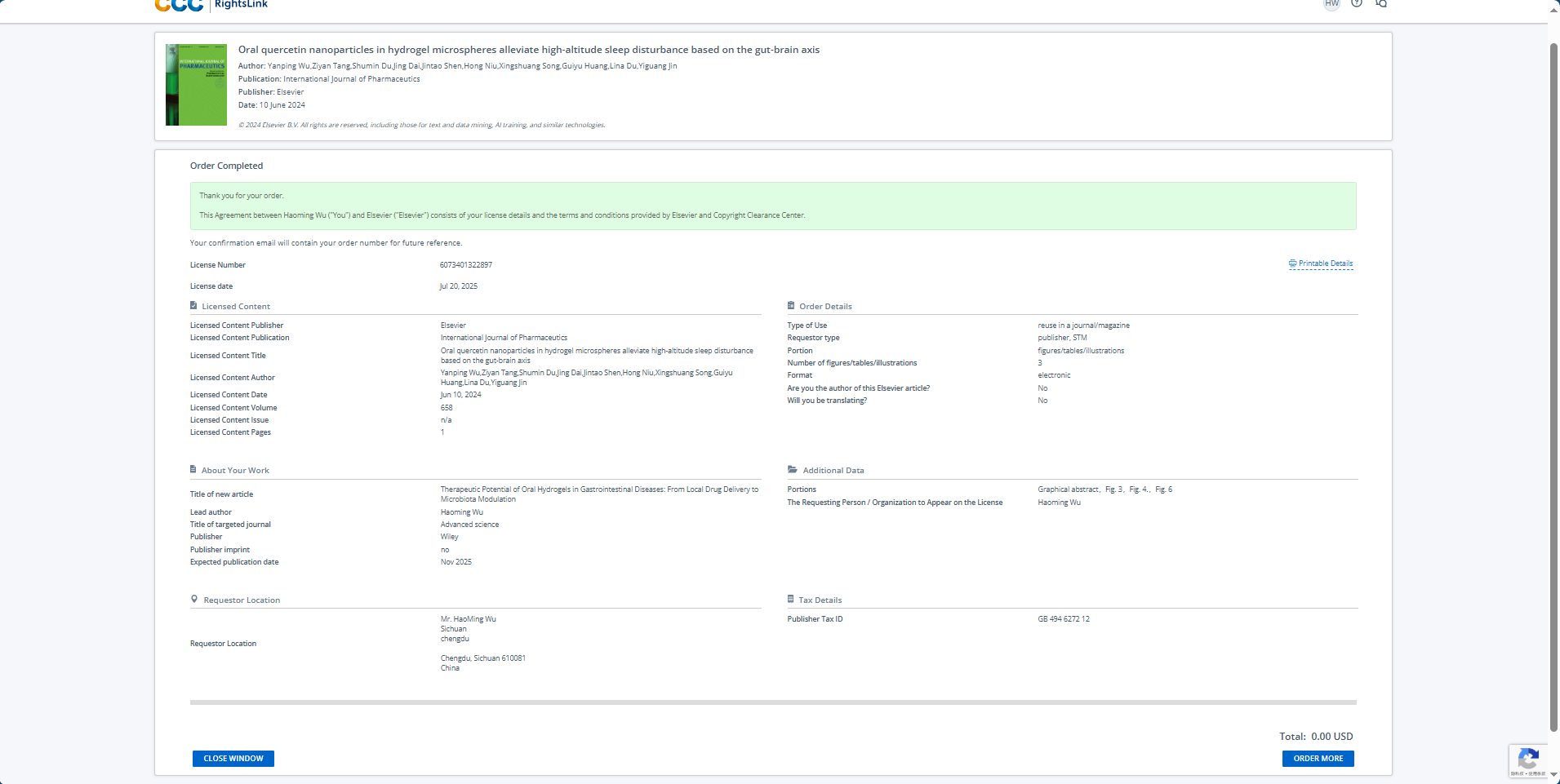
**

**The permissions of Figure 8**

**
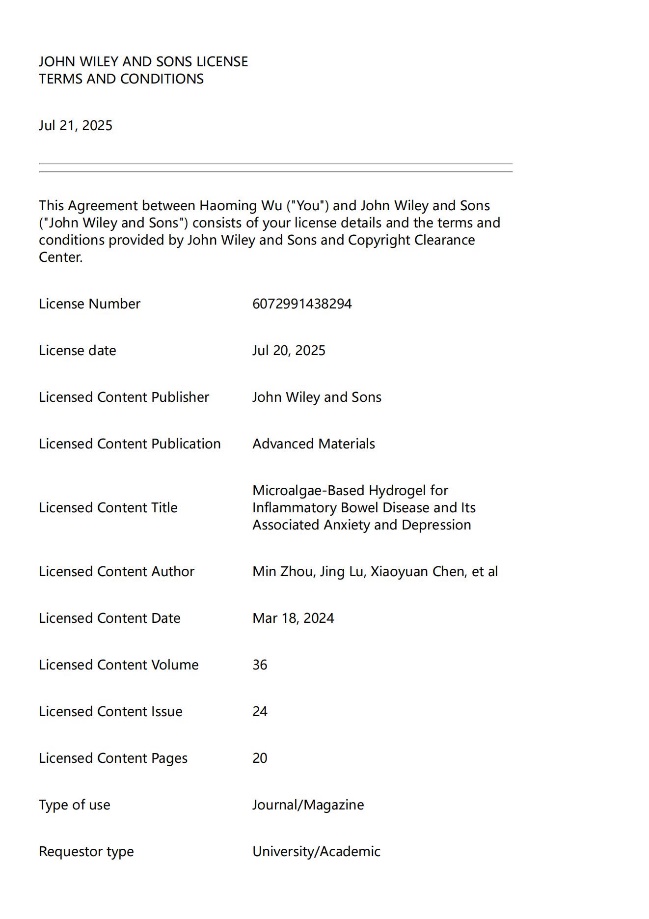
**

**The permissions of Figure 9**

**
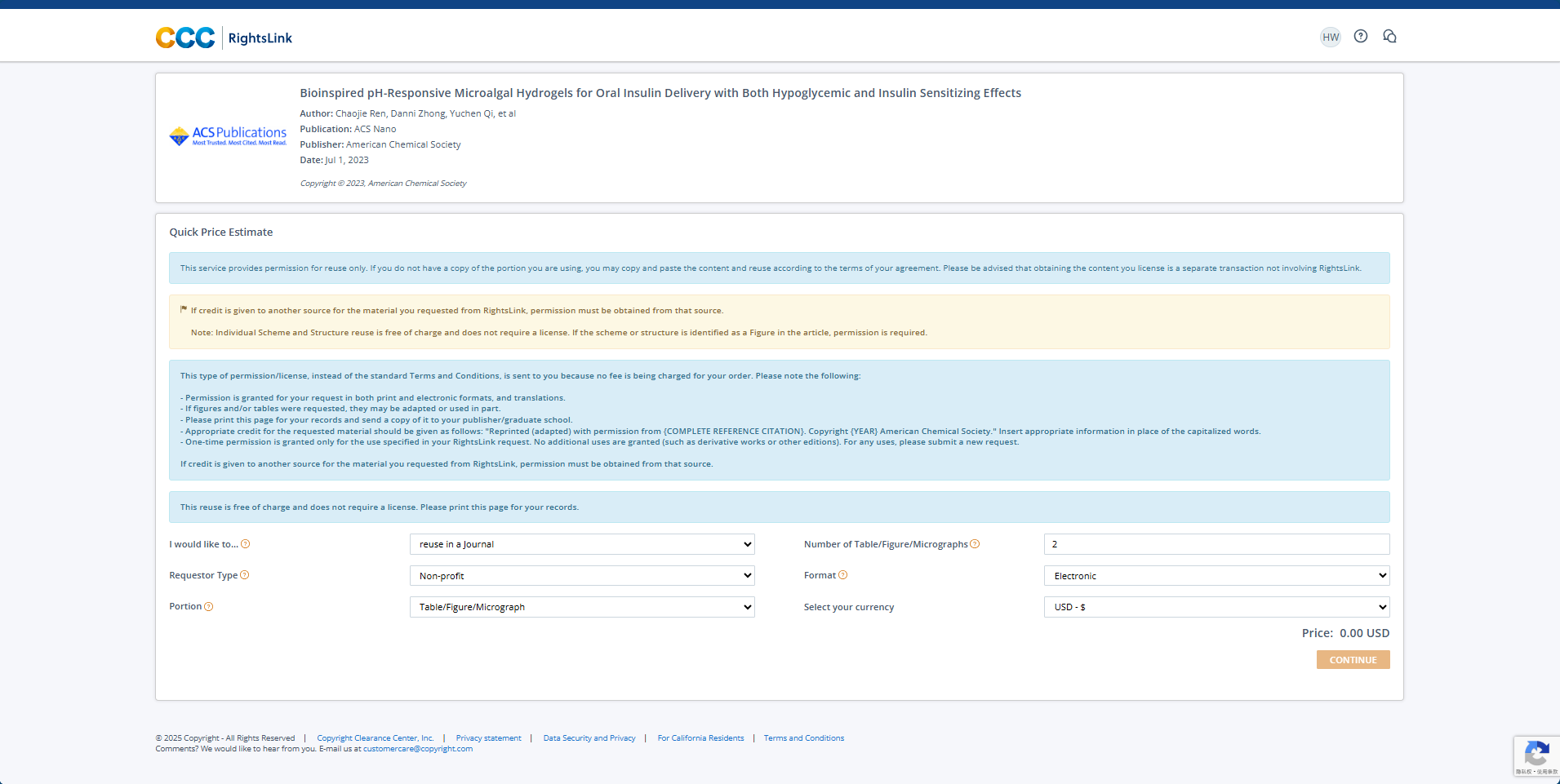
**

**The permissions of Figure 10**

**
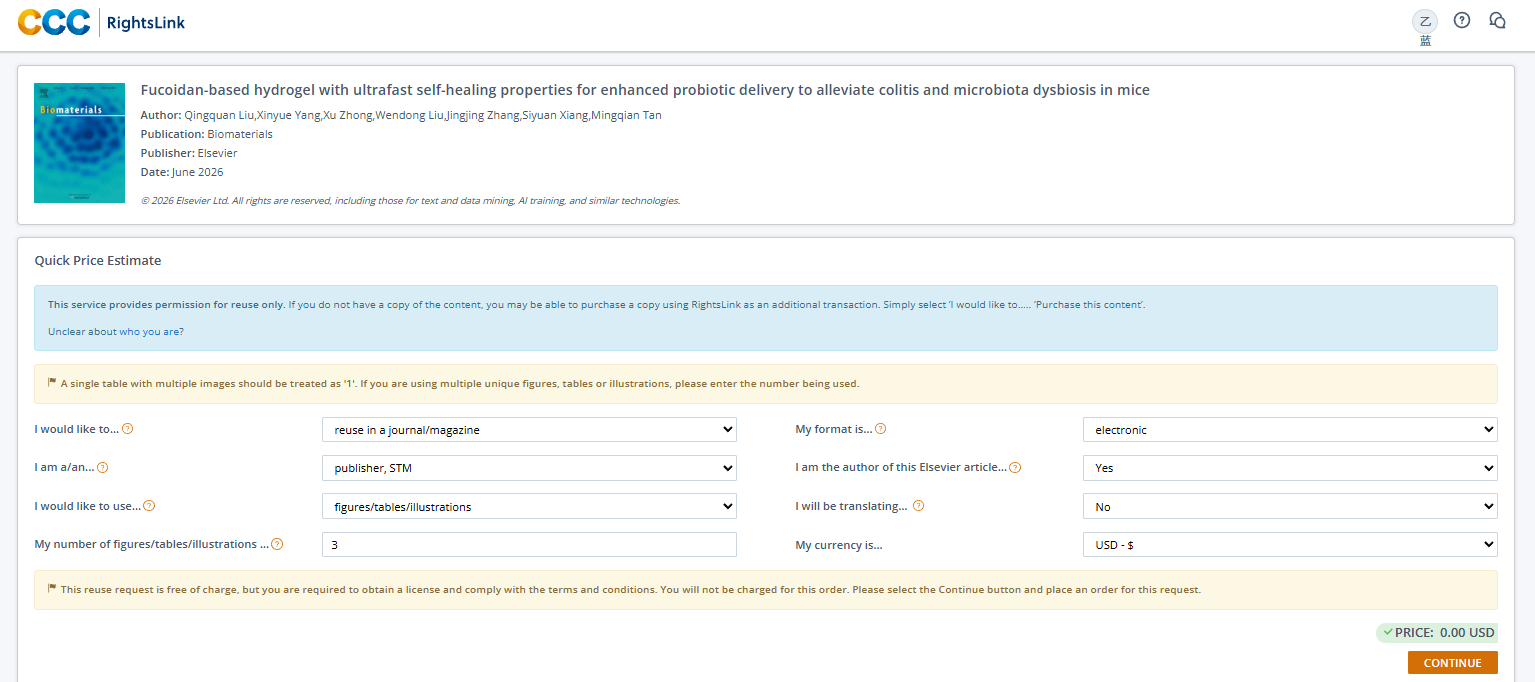
**

**The permissions of Figure 11**

**
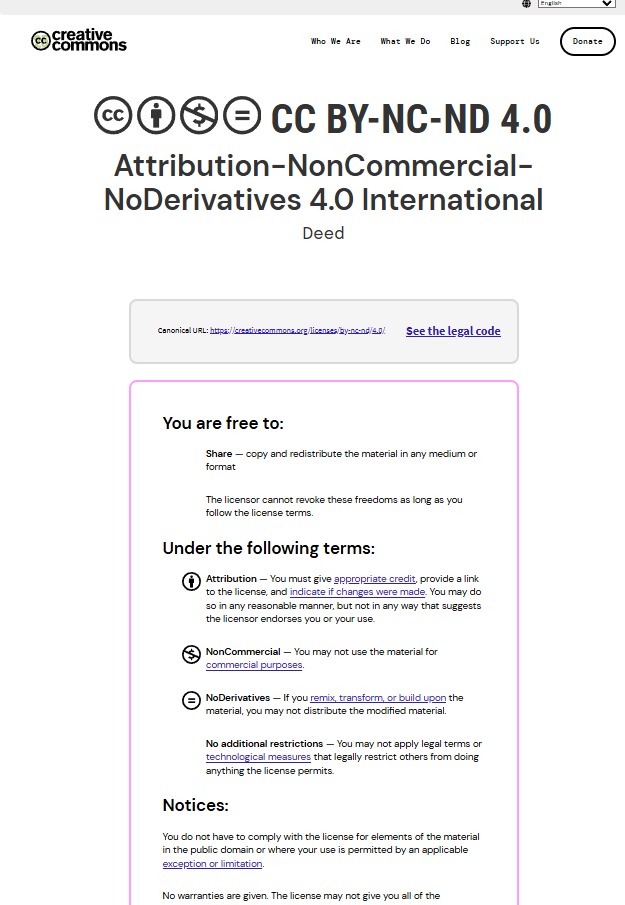
**

**The permissions of Figure 12**

**
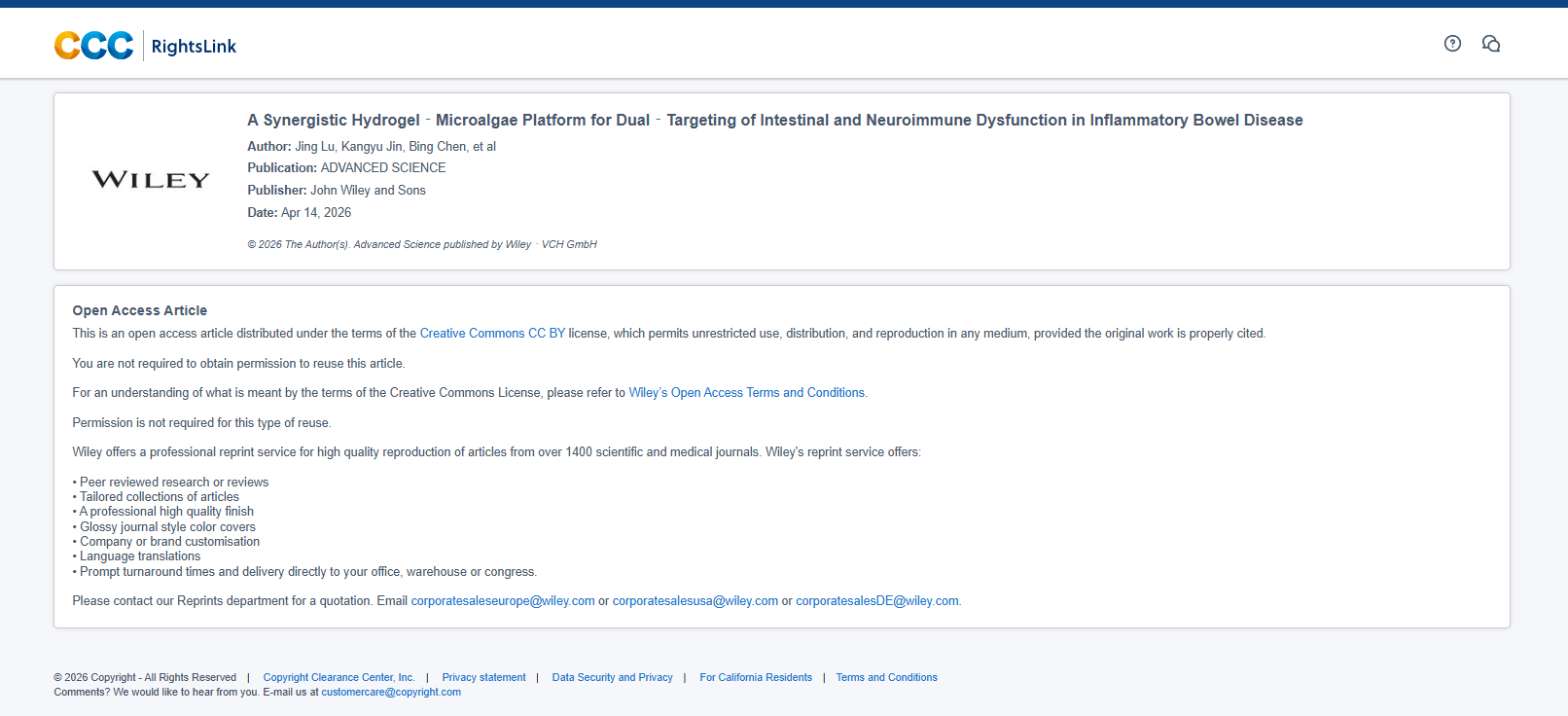
**

**The permissions of Figure 13**

**
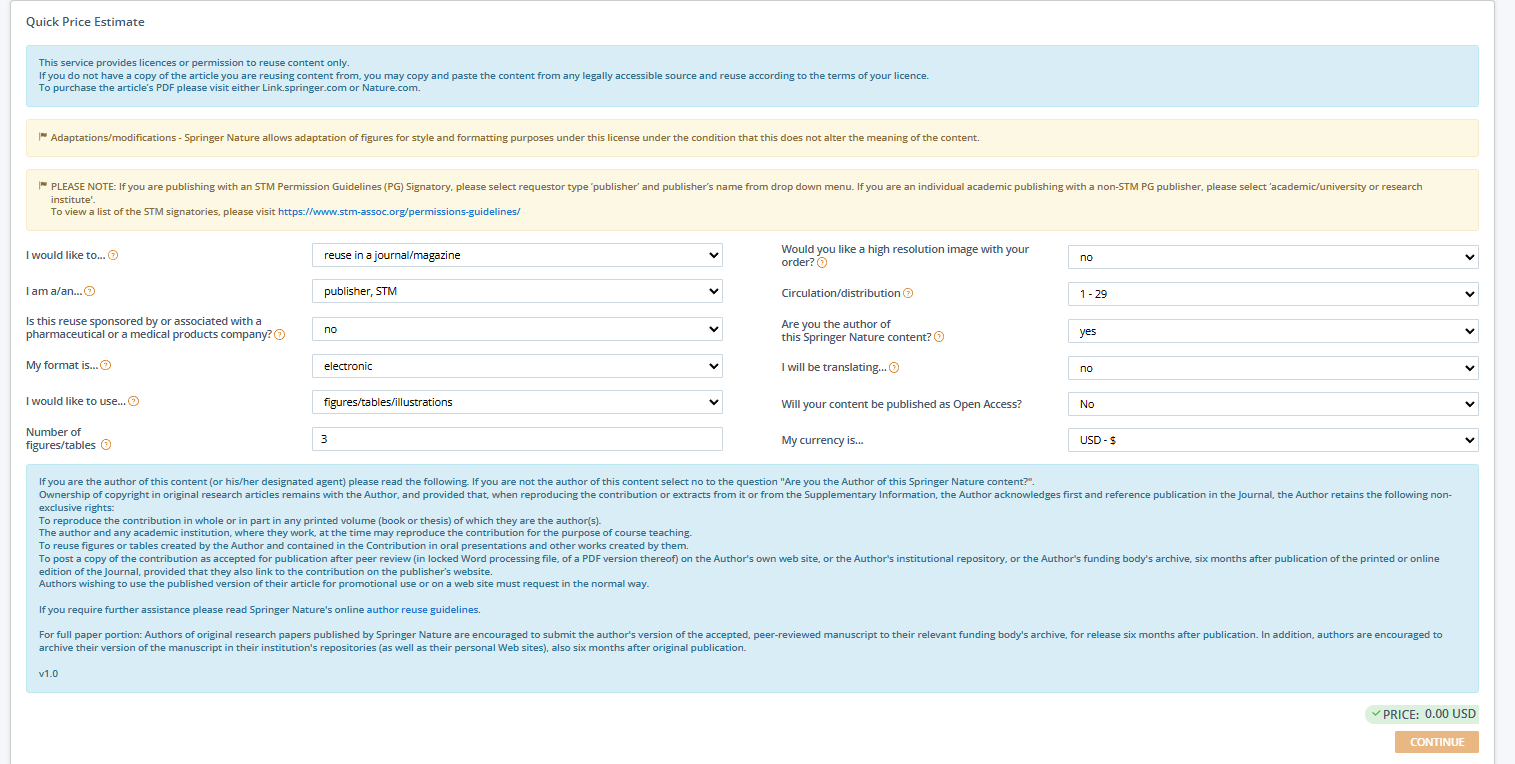
**

**The permissions of Figure 14**


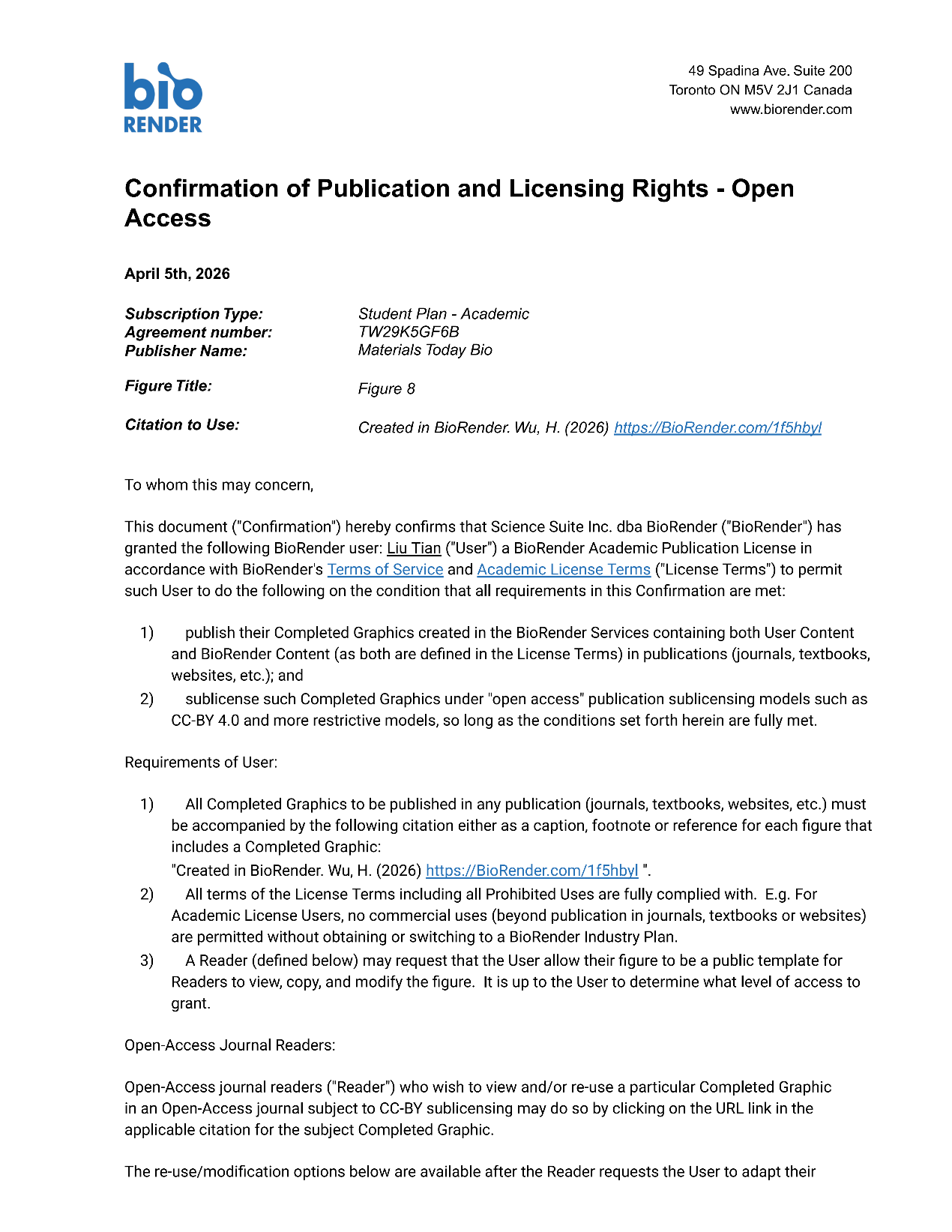


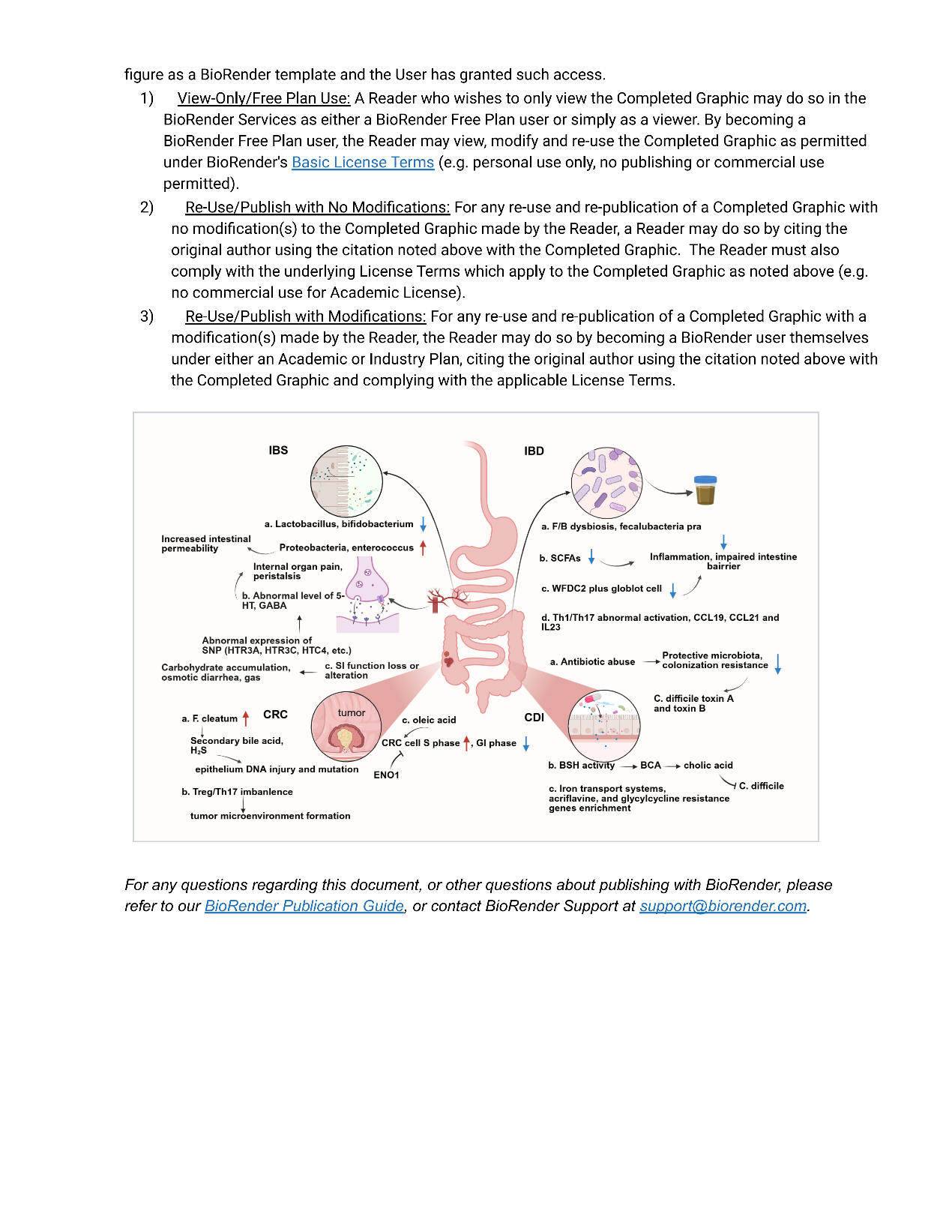

Supplement: Multimedia component 1 [file mmc1.docx]
